# Harnessing Molecular and Bioactivity Network Analysis to Prioritize Antibacterial Compound Isolation From Ant‐Associated Fungi

**DOI:** 10.1002/pca.3513

**Published:** 2025-01-30

**Authors:** Ángel S. Aguilar‐Colorado, Jesús Morales‐Jiménez, José Rivera‐Chávez

**Affiliations:** ^1^ Departamento de Productos Naturales, Instituto de Química, Universidad Nacional Autónoma de México Ciudad Universitaria, Circuito Exterior Ciudad de México Mexico; ^2^ Departamento El Hombre y su Ambiente Universidad Autónoma Metropolitana Ciudad de México Mexico

**Keywords:** ant, anthill, antibacterial compound, *Atta*, bioactivity network, cloud forest fungi, molecular network, *Solenopsis*

## Abstract

**Introduction:**

Antimicrobial resistance is a global public health problem that requires the development of new bioactive compounds. In this context, metabolomic analyses can expedite the research of fungal metabolites as a valuable resource.

**Objectives:**

To investigate the metabolic profiles and isolate antibacterial compounds from micromycetes associated with Mexican cloud forest ants by utilizing network analysis of their chemical and bioactivity data.

**Material and Methods:**

248 fungal strains isolated from six ant's species, soil of their anthills, and soil of the surroundings were evaluated for their in vitro inhibition growth of extensively drug‐resistant *Acinetobacter baumannii* and hypervirulent *Klebsiella pneumoniae* ; subsequently, their metabolites were dereplicated and analyzed by molecular networking and compound activity mapping from spectrometric data. Prioritization of some fungi for isolation of their major constituents was performed, and their structures were established by spectroscopic and spectrometric analysis and their bioactivity determined.

**Results:**

From the fungal collection, 15 secondary metabolites (**1**–**15**) were dereplicated, and 10 compounds (**16**–**25**), including the new (*E*)‐tridec‐7‐ene‐3,5,6,10‐tetraol (**25**), were isolated from *Ascomycetes* of *Trichoderma*, *Cladosporium*, and *Clonostachys* genera. Compounds **16**–**18** stood out for being bioactive. This study is the first report of antibacterial activity against *A. baumannii* for the tricyclic pyridin‐2‐ones deoxy‐PF1140 (**16**) and PF1140 (**17**), with minimum inhibitory concentration of 50 μg/mL.

**Conclusion:**

Network analysis and dereplication proved effective in bioprospecting for antibacterial compounds, offering valuable insights into the chemical diversity of cloud forest soil fungi and their potential applications. Moreover, this study broadens the knowledge of fungal secondary metabolites linked to leafcutter, fire, and warrior ants.

## 1 Introduction

Estimates of the number of species on Earth place the fungi kingdom among the richest [1]. However, despite their biological diversity and cosmopolitan presence, the chemical constituents of most fungal species remain unexplored, and, consequently, the exploitation of their chemical diversity is limited [2]. Natural products possess an incomparable variety of chemical structures with useful biological activities in medicine and agronomy [3, 4]. In particular, fungal secondary metabolites are a promising source of drugs or structural prototypes, particularly in the development of antimicrobial, immunosuppressive, antidiabetic, and anticancer treatments, among other applications [3, 5]. Their metabolites are advantageous because of their drug‐like physicochemical properties, high structural diversity, and unique scaffolds [6, 7]. Furthermore, fungi are a remarkable source of antibiotics given the defense or communication molecules they synthesize to survive in their environments [8, 9].

Today, the development of resistance to antibiotics by pathogenic bacteria is one of the greatest threats to global public health due to the limited availability of treatments. The most concerning multiresistant bacteria are those causing the highest number and more severe infections, for example, *Enterococcus faecium* , *Staphylococcus aureus* , *Klebsiella pneumoniae* , *Acinetobacter baumannii* , *Pseudomonas aeruginosa* , and *Enterobacter* spp., collectively referred to as the ESKAPE group, and are included in the priority attention list of the World Health Organization (WHO) [10, 11]. In this context, fungi associated with less explored environments can be exploited through bioprospecting studies to discover antibacterial compounds as has been done, for example, with cenotes or the deep seabed [12, 13]. Even the soil, hosting strains of *Penicillium*, *Cephalosporin*, and *Aspergillus*—renowned for their ability to synthesize antibiotics—remains a largely unexplored resource with untapped potential awaiting to be discovered [14]. The diversity of fungi in soil has been well documented, and it is known to be dependent on climatic factors, except for mycorrhizae, which are linked to plant richness [15]. In this context, ants and their nests represent an understudied soil environment, rich in fungal and chemical diversity, that gaps investigation for new antibiotic discoveries [16, 17]. Among the ~14,000 ant species known worldwide, in terms of fungal diversity the most studied belong to the *Attini* tribe. The isolated filamentous and yeast‐like fungi are summarized in 360 species, from 173 genera, being *Trichoderma*, *Penicillium*, *Aspergillus*, *Candida*, and *Cryptococcus* the most isolated genera, for which chemical information available is limited, but highlights the ability of these microorganisms to synthesize complex structures whose ecological role is unknown [18, 19].

In this research, filamentous fungi isolated from ants' bodies (entomogenous) and soil of their nests within a Mexican cloud forest (tropical montane cloud forest) were fermented and tested against multidrug‐resistant *A. baumannii* and one hypervirulent strain of *K. pneumoniae* , aiming to prioritize the most promising fungal strains for isolation of antibacterial molecules [20, 21, 22]. These pathogens are of particular interest for their presence in clinical areas, food, and animals, both domestic and livestock [11, 23, 24].

The prioritization process was guided by molecular network analysis and compound activity mapping, which facilitated the identification of structural similarities within the chemical compositions and the presumptive identification of bioactive molecules [25, 26]. This approach led to the identification of 15 molecules and the isolation of 10 compounds from six ascomycetes. Among the isolates, a new chemical entity is reported, alongside several metabolites exhibiting antimicrobial, phytotoxic, cytotoxic against human cancer lines, α‐glucosidase inhibitory activity, and others. Notably, a pair of tricyclic pyridin‐2‐ones showed promising antibacterial activity against a multiresistant *A. baumannii* .

Furthermore, network analysis provided a comparative framework for understanding the chemical diversity within fungal communities in ant nests, their associated entomogenous fungi, and nearby soil. Compound activity mapping revealed the potential diversity of bioactive compounds in the collection, underscoring the value of this integrated approach in discovering novel antimicrobial agents.

## 2 Materials and Methods

### 2.1 Instruments

High‐resolution mass spectrometry (HRMS) by direct analysis in real time (DART) in positive mode were acquired on an JEOL AccuTOF JMS‐T100LC (Akishima, Japan) equipment with time‐of‐flight analyzer (TOF); samples were dissolved in methanol. One‐ and two‐dimensional nuclear magnetic resonance (NMR) spectra ^1^H, ^13^C, COSY, HSQC, HMBC, and NOESY were acquired at room temperature on Bruker Avance III 400‐MHz equipment or Bruker Avance 700‐MHz (Billerica, Massachusetts, USA) equipment. Experiments were recorded in the deuterated solvents chloroform, methanol, acetone, or dimethylsulfoxide (DMSO). RMN spectra were referenced to the residual solvent signal. Optical rotation of the samples dissolved in methanol was determined in a PerkinElmer 343 polarimeter (Waltham, Massachusetts, USA).

Metabolomic analyses were performed in a high‐performance liquid chromatography (HPLC) system coupled to a HRMS^2^ with electrospray ionization (ESI) and quadrupole‐TOF (QTOF) analyzers, as well as an ultraviolet (UV) and visible light absorption detector by photodiode array (PDA), Agilent model G6530BA (Santa Clara, California, USA). Flash chromatography (FC) was performed in a BÜCHI instrument model Pure C‐810 with PDA detector (Flawil, Sweden). Preparative or semipreparative liquid chromatography was carried out in a HPLC Waters (Milford, Massachusetts, USA), which consists of an autosampler, a PDA detector (model 2998), and an evaporative light scattering detector (2424; ELS), as well as a quaternary gradient pump (2535); the control of the equipment, the acquisition, and management of data were carried out with Empower 3.0.

For antibacterial assays, a New Brunswick Scientific Excella E24 shaker (Fisher Scientific, Waltham, Massachusetts, USA), a BioTek Cytation 5 microplate reader (Agilent, Santa Clara, California, USA), and a MULTISKAN SkyHigh (Thermo Fisher Scientific, Waltham, Massachusetts, USA) microplate reader were used.

### 2.2 Solvents and Reagents

The solvents used in the macerations and liquid/liquid separations were of industrial grade, properly distilled. The solvents for chromatographic separations were HPLC grade, acetonitrile purchased from Supelco (Bellefonte, Pennsylvania, USA), methanol from Sigma‐Aldrich (Burlington, Massachusetts, USA), and formic acid from Fermont (Monterrey, Nuevo León, México). For spectrometric analysis, acetonitrile of such grade from Honeywell (Charlotte, North Carolina, USA) and water Milli‐Q grade were employed. Of reactive grade, DMSO (Fermont), gentamicin sulfate (GOLDBIO; St Louis, Missouri, USA), and colistin sulfate (Sigma‐Aldrich) were used. The potato dextrose (PD) agar and broth were purchased from MCD LAB (San Jacinto Amilpas, Oaxaca, México) and the Muller Hinton (MH) broth from Condalab (Madrid, Spain); all growth media were prepared with distilled water. The cereal for solid cultures was Cheerios honey from Nestle (Vevey, Switzerland). Deuterated solvents were purchased from Sigma‐Aldrich.

### 2.3 Study Area and Collection of Soil and Ants

Ant nests were selected from cloud forest reserve and certain disturbed areas located in the municipalities of Xalapa de Enriquez, Coatepec, and Teocelo, Veracruz, Mexico (Table S1). The collection was open to any ant and their nest found at ground level along roads or paths at the surveyed locations. From each active anthill, two 40‐mL soil samples were collected, one from the area near the entrance, transit area, or mound and the other 5 m from it, where ant activity was not observed, without considering the leaf litter and from the surface to a depth of 10 cm. The interior of two nests was also sampled, in which case soil was collected from the walls of a tunnel that was found by digging horizontally from a trench next to a mound. The soil was kept in airtight containers, protected from light, and refrigerated at 0°C–5°C, during fieldwork until cultivation. The shovels and spatulas used were washed with water and sterilized by rinsing with absolute ethanol before each harvesting [27, 28]. Additionally, ants of indistinct caste were collected from each nest and maintained in absolute ethanol for taxonomic determination. A second sample of ants was kept alive in an airtight container, protected from light, and refrigerated for less than 12 h until processing. The insects were treated according to the capture and conservation techniques described in the literature [29].

The ants were identified to species level according to morphological keys, and the specimens were deposited in the Entomological Collection IEXA, in Xalapa, Veracruz, Mexico, with the accession identifier IEXA‐2024‐IM12.

### 2.4 Fungal Strains Isolation and Identification

For soil samples, a dilution technique limited to the isolation of the filamentous fungal population capable of growing on PD agar medium was applied, as designed based on previously reported methods [27, 28]. A suspension 1:10 w/v in sterile distilled water was prepared from each sample and serially diluted 1:10 v/v four times. Then, 350 μL of the three least concentrated suspensions were plated with a replicate in 10‐cm‐diameter Petri dishes with PD agar supplemented with gentamicin at 150 μg/mL. The cultures were incubated for several weeks at room temperature under normal light–dark cycles, and axenic morphotypes were obtained for each sample by consecutive transfers. As adapted from a published procedure, entomogenous fungi from ants required sterilization of their exoskeleton by immersion in absolute ethanol for 120–150 s and rinsing with sterile distilled water [30]. Dried specimens were kept refrigerated. For culture, 9–90 ants from each nest were crushed in 350 or 500 μL of sterile distilled water. Subsequently, the suspension was plated on PD agar supplemented with gentamicin. Axenic morphotypes were obtained as previously stated. In this research, morphotypes refer to a classification based on macroscopic characteristics of the fungus strains such as size, elevation, edge, color, mycelial type, presence of exudates, and shape of their colonies [31, 32]. All morphotypes were assigned an alphanumeric key from IQ‐751 to 1538 and deposited in a fungal collection of the research group (Tables S2 and S3).

Taxonomic determination of selected fungi was performed by molecular sequencing of the internal transcribed spacer region (ITS) of each morphotype. Sequences were deposited in GenBank database (Table S11). DNA extraction, amplification, sequencing, and alignment analysis for identification was performed as described by Martínez‐Aldino et al. [33].

### 2.5 Fungal Cultivation and Extraction

For screening purposes, all cultures were performed on a small scale (Table S4). First, a liquid culture of each morphotype was prepared in 5 mL of PD broth in hermetically sealed plastic tubes, inoculated with a 1 cm^2^ piece of agar with mycelium, and incubated for ~11 days under orbital shaking at 100 rpm, with normal light–dark periods at room temperature. Subsequently, the liquid culture was transferred to a solid media (3 g of cereal Cheerios) contained in plastic tubes or 25‐mL Erlenmeyer flasks and incubated for ~25 days under static conditions. After fermentation process, all samples were extracted by maceration with shaking at 100 rpm for 24 h with a 50:25:25 mixture of ethyl acetate–methanol–dichloromethane. The extraction solvent was filtered through degreased cotton and brought to dryness to generate the extract [34, 35]. Additionally, control culture extracts were obtained from the growth mediums. For chemical studies, the culture process was scaled up to medium size. It was carried out following the procedure described for the small version but adjusting the necessary to a variable number of cultures with 9 g of cereal in 250‐mL Erlenmeyer flasks (Table S12). The extraction process was performed adjusting the solvent volume.

### 2.6 Untargeted Metabolomic Studies

The extracts were dissolved in DMSO at a concentration of 2 mg/mL and analyzed by HPLC‐QTOF‐HRMS^2^. Chromatographic separations were performed on a Gemini phase NX‐C_18_ column (2 × 75 mm, particle size 3 μm) Phenomenex (Torrance, California, USA) at 30°C. The mobile phase, at a flow rate of 0.4 mL/min, consisted of a mixture of acetonitrile and 0.1% water with formic acid using a gradient that started with a linear increase from 15% to 100% acetonitrile in 8 min and was maintained at 100% acetonitrile for 1.5 min before returning to initial conditions. The electrospray ionization (ESI^+^) source was operated in positive mode. Nitrogen at 275°C and 300°C, respectively, was used as auxiliary and envelope gas. The capillary voltage was 4.5 kV, and the nozzle voltage was 1 kV. Spectra were acquired with the analyzer in full scan mode over a range *m*/*z* 100 to 2500, and for tandem analysis, the two most abundant precursors per cycle were selected and fragmented by collision‐activated dissociation at a normalized energy of 30 eV [33, 35].

Classical molecular networks were obtained with the Global Natural Products Social Molecular Networking (GNPS), and their construction is based on the similarity of the fragmentation patterns of precursor ions along the total ion chromatograms of the extract collection [36]. Molecular networks are graphical representations of chemical diversity detected by HRMS^2^, and in the classical version, spectrometric data are grouped between samples using an algorithm called MSCluster [37]. Likewise, the nodes of the networks were dereplicated by comparing their consensus fragmentation patterns against information in the platform database; dereplication refers to the putative identification of known compounds [36]. The tandem mass spectra were processed with the ProteoWizard MSConvert Version 3 program to annotate the peaks using the Vendor algorithm and transform the files to the extension mzML [38]. 248 spectra of filamentous fungi were uploaded to the GNPS server, and classical networks were constructed with the basic parameters of 0.02 Da as the mass tolerance of the precursor ion and fragment ions, 0.7 as the minimum cosine score, a minimum of six fragment ions to connect two consensus spectra, 1999 Da as the maximum difference of the precursor ion between two neighboring nodes, a maximum of 10 connections per node, two as the minimum cluster size, and 100 as the maximum number of nodes in a connected network. The cosine score, or cosine similarity, is a metric used to quantify the similarity between two mass spectra. It ranges from 0 to 1, where 0 indicates no similarity (completely different spectra) and 1 indicates identical spectra. For the library search, three was initially selected as the minimum number of tandem mass spectrometry (MS^2^) fragments to share with the reference spectrum, as well as a minimum cosine similarity of 0.7. Before constructing the network, signals from the growth medium extracts and DMSO were subtracted to eliminate background noise and ensure the specificity of the detected compounds. The networks were mapped according to the origin of the morphotypes. Visualization of networks was performed in Cytoscape 3.10.3 [39].

The GNPS dereplication of the consensus nodes was manually verified for each spectrometric acquisition, and the spectrometric data of identified molecules were also searched in MassBank [40]. For identification of a compound, the exact mass of the precursor ion had to match with a variance of less than 5.1 ppm, a mass difference of less than 0.003; at least 5 fragments were shared with the reference spectrum; and the cosine similarity among spectra had to be greater than 0.7. In addition, the libraries were limited to Class 1 records, performed with ESI^+^ and from authentic samples indexed; the spectrometric information of the Class 1 records comes from samples that have been peer‐reviewed for integration into the platform.

Compound activity mapping was performed using the NP Analyst platform. This analysis uses scores based on Pearson correlation coefficients and arithmetic means to correlate HRMS^2^ and bioactivity data from a group of samples. It facilitates the putative identification of *m/z* ratios associated with biological activity. The resulting graphical output is a bioactivity network, showing the presumed bioactive *m/z* ratios and the extracts in which they were identified [25]. Only the results of bacterial growth inhibition of *A. baumannii* were considered in this analysis. The inhibition percentages were normalized between 0 and 1 for each replicate. Spectrometric data consisted of the classical molecular network generated by GPNS. The cutoff value of the activity and clustering score was 0.25 and 0.1, respectively, for network visualization. The network was edited and visualized in Cytoscape 3.10.3, as well as on the NP Analyst website.

### 2.7 Compound Isolation

Primary fractionation was performed by liquid–liquid extraction. Each extract was dissolved in the minimum necessary volume (120‐300 mL) of a 1:1 mixture of dichloromethane and 8:2 water–methanol. The water–methanol phase was then extracted at least 4 additional times with an equivalent volume of dichloromethane. The phases were filtered through defatted cotton, and the dichloromethane phase was additionally pass through anhydrous sodium sulfate; all were brought to dryness *in vacuo*. In a subsequent stage, the dichloromethane phase was dissolved in the minimum necessary volume (< 40 mL) of 1:1 acetonitrile–methanol and defatted with a similar volume of hexane at least 5 times. These phases were also dried, and their yields were determined.

Secondary fractionation was carried out by FC. C_18_ modified silica gel with a particle size of 25 μm Biotage SNAP Ultra (Uppsala, Sweden), packed in cartridges and columns of variable size, was used as a stationary phase. Samples were fractionated in solution or absorbed on variable mixture of Celite 545 and silica gel 60. Elution proceeded with gradients of 0.1% formic acid in water and either methanol or acetonitrile. This was followed by rinses with methanol or an 8:2 mixture of methanol–dichloromethane. The PDA detector acquired at 254, 360, 450, 580, and in scanning mode from 200 to 800 nm. Eluates were automatically collected in volumes ≤ 50 mL, based on changes in the UV absorption maximum. Subsequent analysis of the chromatogram facilitated grouping of the eluates, culminating in the final fractions.

The major constituents of the extracts were isolated by preparative or semipreparative HPLC. Modified silica gel NX‐C_18_ of 110 Å particle size packed in a Phenomenex Gemini column of 250 mm length and 21.6 mm diameter and Phenomenex Gemini column of 250 mm length and 10 mm diameter were used as stationary phase for preparative and semipreparative chromatography, respectively. Various gradients at a flow rate of 13–15 mL/min for preparative chromatography and 4.6 mL/min for semipreparative chromatography composed of binary mixtures of 0.1% formic acid in water, methanol, and acetonitrile were used as mobile phase. Samples were dissolved at a minimum volume of 1:1 dioxane–methanol mixture to get a 30–120 mg/mL concentration for preparative chromatography and a maximum volume of 500 μL was injected manually by elution. The PDA detector acquired at 254 nm and in scanning mode from 200 to 800 nm. A more detailed description of the isolation of each compound can be found in Section S14. Spectrometric (HRMS) and spectroscopic information used for compounds elucidation (RMN spectra) can be consulted in Sections S15–S20.

(*E*)‐Tridec‐7‐ene‐3,5,6,10‐tetraol (**25**): yellow liquid; [α]^25^_D_ + 11.7 (*c* 0.6, methanol); ^1^H NMR (deuterated chloroform; 700 MHz) δ 0.94 (3H, t, *J* = 7.42 Hz, H‐1), 1.56 (1H, ddd, *J* = 13.66, 7.48, 6.4 Hz, H‐2a), 1.71 (1H, ddd, *J* = 13.87, 7.48, 6.56 Hz, H‐2b), 4.00 (1H, dq, *J* = 7.85, 6.67 Hz, H‐3), 1.63 (1H, ddd, *J* = 12.69, 7.74, 6.45 Hz, H‐4a), 2.35 (1H, dt, *J* = 12.58, 6.78 Hz, H‐4b), 4.11 (1H, td, *J* = 6.56, 5.27 Hz, H‐5), 4.15 (1H, t, *J* = 6.13 Hz, H‐6), 5.55 (1H, ddt, *J* = 15.27, 6.96, 1.34 Hz, H‐7), 5.78 (1H, dddd, *J* = 15.49, 7.74, 6.82, 1.09 Hz, H‐8), 2.28 (1H, m, H‐9a), 2.16 (1H, dt, *J* = 15.38 7.58 Hz, H‐9b), 3.67 (1H, m, H‐10), 1.45 (2H, overlapped, H‐11), 1.36 (1H, q, *J* = 7.53 Hz, H‐12a), 1.45 (1H, overlapped, H‐12b), 0.93 (3H, t, *J* = 7.10 Hz, H‐13); ^13^C NMR (deuterated chloroform; 175 MHz) δ 10.3 (CH_3_, C‐1), 29.7 (CH_2_, C‐2), 79.0 (CH, C‐3), 39.8 (CH_2_, C‐4), 76.8 (CH, C‐5), 85.3 (CH, C‐6), 131.9 (CH, C‐7), 130.1 (CH, C‐8), 40.5 (CH_2_, C‐9), 70.8 (CH, C‐10),39.1 (CH_2_, C‐11), 19.0 (CH_2_, C‐12), 14.2 (CH_3_, C‐13); positive ion HRMS‐ESI‐QTOF *m/z* 229.1812 (calcd for C_13_H_25_O_3_ [M+H‐H_2_O]^+^, 229.1803, Δ = +3.9 ppm). Spectra available in Section S19.

### 2.8 Antibacterial Assay

*A. baumannii* strain A564 and *K. pneumoniae* strain serotype K2 are Gram‐negative bacteria of intrahospital origin resistant to conventional drugs; A564 strain is multiresistant except for colistin, and strain K2 strain is hypervirulent [41, 42]. Bacterial cultures were cryogenically maintained at −80°C in a 4:1 mixture of MH broth and glycerol. Growth inhibition assays were performed based on the Clinical and Laboratory Standards Institute (CLSI) guidelines for broth microdilution assays [43]. Bacterial cultures were grown in MH broth incubated for 16–24 h with shaking at 37°C. From the liquid culture, 1:20 bacterial dilution in MH broth was prepared from a suspension of 0.5 units of the McFarland scale by adjustment to an optical density at 600 nm of 0.08–0.13 (equivalent to 1.5 × 10^8^ colony‐forming unit or CFU/mL *Escherichia coli* ). All assays were performed on a 96‐well flat bottom plate. DMSO was used as a vehicle, and the positive control was gentamicin and colistin. The extracts were evaluated at a final concentration of 250 ppm and isolated compound at 100 ppm. To evaluate the extracts and the positive control, 2 μL of the sample, 88 μL of MH broth, and 10 μL of diluted bacterial suspension were deposited in each well. To evaluate the growth control, the aliquot of the sample was replaced by vehicle, and in the case of the sterility control, also bacterial suspension was replaced by MH broth. The optical density of the treatments was measured in a microplate reader at 600 nm with continuous shaking for 5–10 s at the beginning of the incubation period and after 16–24 h of growth at 37°C. The inhibition percentage was calculated for each well with respect to the average of the growth control using Equation (1):

(1) $$\begin{array}{c} \text{Percentage of growth inhibition}=\left(1-\frac{{\text{Absorbance}}_{\text{sample}}}{{\text{Absorbance}}_{\text{negative control}}}\right)\left(100\right), \end{array}$$

The results are reported as the average of at least three replicates ± its standard deviation. The minimum inhibitory concentration (MIC) was defined as the smallest concentration with 100% growth inhibition by determining the effect of six concentrations of the compound from 0.01 to 200 μg/mL [43]. The minimum bactericidal concentration (MBC) was determined by counting CFU of the concentrations that showed 100% inhibition in the bacterial growth test; the inoculum was seeded on MH agar and incubated under the conditions described for the previous experiment, adapted from the guidelines of American Society for Microbiology [44]. MBC is defined as no more than 0.1% of starting CFU's growth in negative control wells.

### 2.9 Statistical Analysis

Analysis performed with IBM SPSS Statistics Version 30 (IBM, Armonk, New York, USA). The Shapiro–Wilk and Kolmogorov–Smirnov tests were used to test for normality. Levene's test was used to determine the equality of variances. For statistical significance, a value of *p* < 0.05 was considered. Plots were generated in GraphPad Prism 8 (GraphPad Software, Boston, Massachusetts, USA). The Sankey diagram was generated with SankeyMATIC web (https://sankeymatic.com). Venn diagrams were built with Bioinformatics and Evolutionary Genomics web (https://bioinformatics.psb.ugent.be/webtools/Venn/).

## 3 Results and Discussion

This research outlines a strategy to explore the biotechnological potential of ant‐associated fungi. Given the exploratory nature of the study, the focus was placed on incorporating fungi from various ant species and their nests, rather than conducting a representative sampling of the studied area. Molecular networking and compound activity mapping were the primary metabolomics techniques employed. These methods were chosen for their effectiveness in unraveling chemical complexity and guiding the isolation of bioactive compounds. In summary, these findings underscore the importance of further bioprospecting the mycobiota associated with ants while emphasizing caution in extrapolating the biological activity and chemical diversity of fungi from other habitats. These points are discussed in detail in the following sections.

### 3.1 Fungal Diversity of Soil, Ants, and Their Nests

**FIGURE 1:**
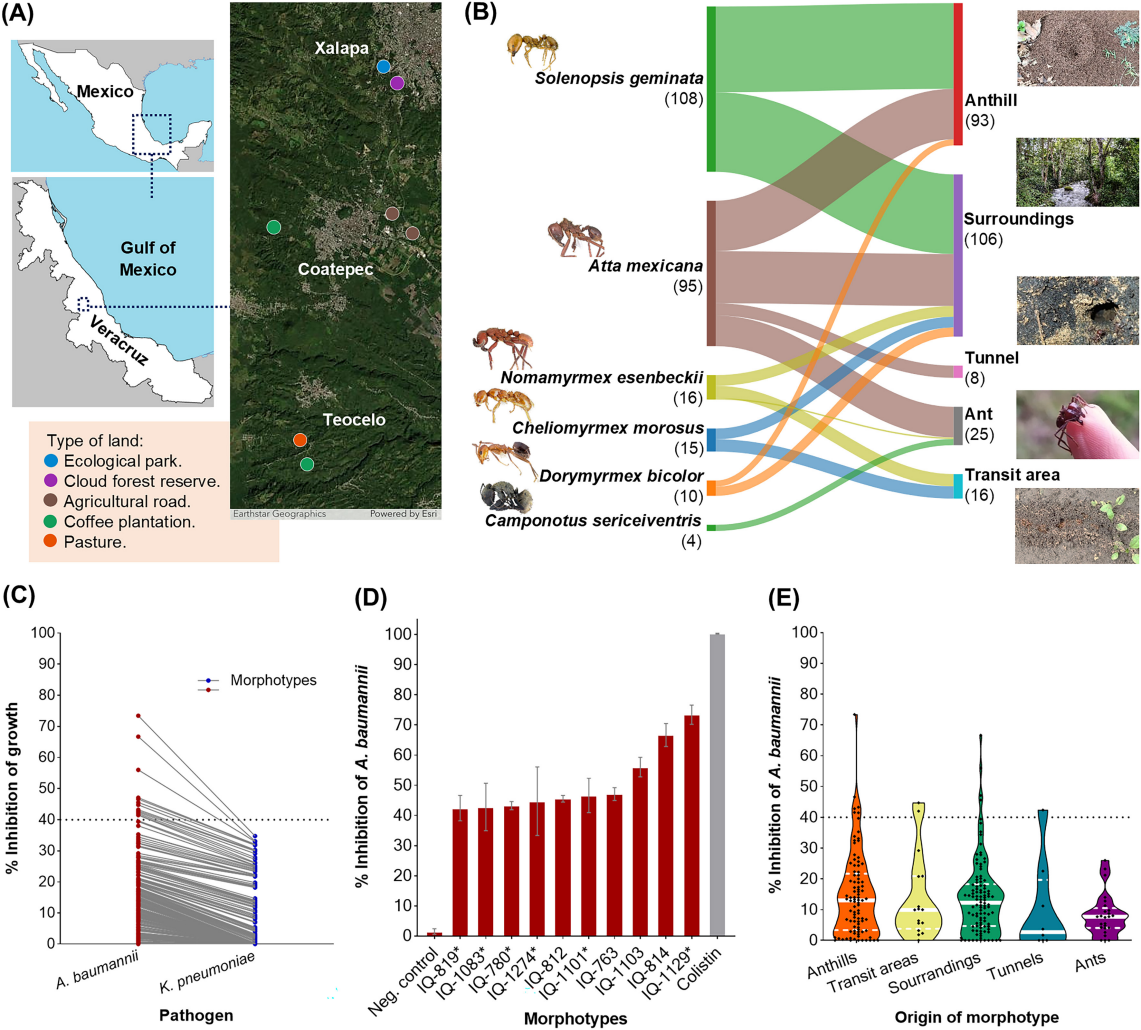
Location of the collection points (A). Distribution of bioprospected morphotypes according to ant species and isolation point. Satellite image powered by Esri (https://www.arcgis.com/home/item.html?id=10df2279f9684e4a9f6a7f08febac2a9) Ants' images were taken and modified from AntWeb v8.106.1 (https://www.antweb.org) (B). Comparison of the antibacterial effect of morphotypes, one line connects the effect of the same morphotype (C). Top 10 values against *Acinetobacter baumannii* in a bar chart (positive control of colistin in water at 20 μg/mL). Asterisks mark morphotypes associated with ants (D). Violin plot of *A. baumannii* inhibition according to morphotype origin. The median is drawn with a continuous line and the quartiles with dotted lines. The medians per group do not differ statistically (Kruskal–Wallis test, *p* = 0.13; E).

A collection composed of 694 filamentous fungi were isolated from a variety of soil and ant samples collected in the cloud forest of Veracruz, Mexico, and several areas whose original vegetation has been modified: pastures, coffee plantations, ecological parks (suburban area), and agricultural roads (rural area; Figure 1A; Table S1) with several level of disturbance along its borders. This variability is reflected in the location of the anthills collected [45]. Six species of ants were found: *Solenopsis geminata* (fire ant) and *Atta mexicana* (leafcutter ant) from the subfamily Myrmicinae; *Nomamyrmex esenbeckii* and *Cheliomyrmex morosus* of Ecitoninae, trivially called army ants; *Dorymyrmex bicolor* from Dolichoderinae; and *Camponotus sericeiventris* from Formicinae, an arboreal ant. It should be noted that the ant species were not intended to be representative of the surveyed areas; however, more than half of the sampled nests correspond to *S. geminata* and *A. mexicana* [46, 47]. Due to the variation in nest structures and the diverse lifestyles of ants, fungal isolates were obtained from different sources: 17 from soil near the nests, 15 from soil within mounds, two from tunnels, two from transit areas of army ants (subfamily Ecitoninae), and entomogenous fungi from four ant species (Table S2). The isolation method allowed obtaining 666 fungal isolates, 17 in average per sampling site. For entomogenous fungi, a total of 28 morphotypes were isolated from all species, except *S. geminata* and *D. bicolor* . An additional variety of morphotypes did not survive laboratory cultivation (59) or subsequent transfer attempts (35). These morphotypes present technical challenges, as their growth may depend on specific conditions, such as the presence of symbionts [48, 49].

Taxonomic identification of fungi was reserved for those of chemobiological interest. Previous studies have shown that the surveyed area exhibits high richness and diversity of microscopic fungi, with *Penicillium* and *Trichoderma* being the most abundant genera [50, 51, 52, 53]. Notably, the mycobiota of disturbed sites undergo modifications. For the areas considered in this research, coffee plantations are the most effective at preserving fungal and ant species richness [54, 55, 56]. Complementarily, studies have explored entomogenous and nest‐associated fungi from *Atta* species to identify entomopathogenic fungi for biological control and to characterize the symbiotic relationships of fungus‐growing ants [19, 57, 58, 59]. Furthermore, studies on the fire ant *Solenopsis invicta* have documented both its entomopathogens and the fungal diversity within its mounds [19, 60, 61, 62, 63, 64, 65].

### 3.2 Screening for Antibacterial Activity

Organic extracts for a random selection of 248 fungi (retaining the proportion of categories from the full collection) were prepared (Table S3). Almost all entomogenous were considered (Figure 1B). The extract yield ranged from 2.9 to 158.3 mg of extract/per gram of substrate; this wide range could be explained in terms of biomass production among morphotypes, for example, to different growth rates or coverage of nutrient requirements by the culture medium [66]. Chemical investigation of the lower yielding fungi was profiled as unlikely (Table S4). Next, the extracts' inhibitory activity against multidrug‐resistant pathogens *A. baumannii* and *K. pneumoniae* was determined (Table S5). Some fungal extracts were able to inhibit the growth of *A. baumannii* by more than 50%, but in the case of *K. pneumoniae* , the maximum inhibition was 34.7% (Figure 1C and Figure S6A). The inhibitory growth effect observed at 250 μg/mL was particularly promising for isolating *A. baumannii* inhibitors, with notable activity demonstrated by morphotypes IQ‐1129 (73.4 ± 3.2%), and IQ‐814 (66.71 ± 3.8%), (Welch’s analysis of variance, *p* < 0.05; *post hoc* Games‐Howell test at 95% confidence level; Figure 1D). The influence of the sampling site in morphotypes bioactivity was statistically evaluated. Nevertheless, no statistical differences were observed (Figure 1E and Figure S6B). Particularly, when the comparison was limited to the best represented ant species ( *S. geminata* and *A. Mexicana* ), no significant differences were observed (Figure S6C). According to the analysis, the bioactive morphotypes are evenly distributed in nearby soil samples, nests, and ants. Conversely, previous studies using next‐generation sequencing and microbial isolation have demonstrated that fungal communities within nests differ significantly from those in the surrounding areas [19, 27, 67]. Notably, the cuticle of *Atta* species yielded few bioactive fungi, perhaps due to the presence of antifungal‐producing actinobacteria on the ants' bodies or the association of *Acinetobacter* spp. and *Klebsiella* spp. with their cuticle. This hypothesis requires further research [16, 68, 69, 70]. Importantly, it is the first study examining the antibacterial activity of mycobiota in collected ant species.

### 3.3 Untargeted Metabolomic Analysis

**FIGURE 2:**
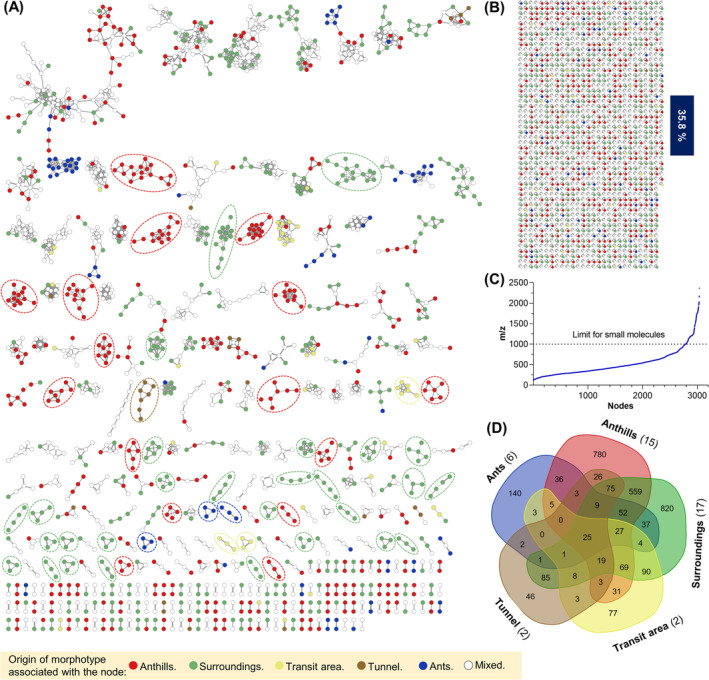
Molecular networks of bioprospected morphotypes. Exclusive nodes according to the isolation point of their morphotype are colored. Networks with three or more nodes and a single color are enclosed with a dotted line (A). Solitary nodes represent 35.8% of the chemical diversity under study (B). The point chart shows *m/z* ratio of the precursor ions of all nodes in ascending order (C). Venn diagram of 3036 nodes forming the molecular networks, by morphotype origin and in parentheses the number of sampling points (D).

The chemical diversity of the 248 fungal extracts selected was analyzed by molecular networking with the nodes classified according to the morphotypes origin (Figure 2A,B); alternative visualizations can be found in Figure S7. Different analysis parameters to build the networks can lead to different topologies; however, for a first approximation, the networks presented are based on the recommendations by GNPS [37]. In a molecular network analysis, the chemical composition is represented as nodes that are connected by edges, and each node is a consensus mass spectrum created from the alignment of those found in HRMS^2^ experiments of the extracts and represents a different compound. Solitary nodes correspond to consensus spectra (or cluster), with no fragmentation similarities with the rest, interpreted as coming from unusual chemical structures. Furthermore, networks are groups of interconnected nodes, which correspond to families of consensus spectra, understandable as structural derivatives [71]. Visualizing the chemical diversity of the screened morphotypes reveal the metabolic potential of the bioprospected mycobiota. In this analysis, solitary nodes represent up to one third of the total and the rest are organized in networks; certain networks composed entirely of nodes with a common origin can be identified. More than 90% of the nodes represent small molecules (*m/z* of precursor ion < 1000 Da; Figure 2C) [72].

Moreover, molecular network analysis allowed for the comparison of chemical diversity across various fungal sources in the collection, revealing that some compounds were exclusively accessible through the isolation of fungi associated with ant nests. The chemical diversity of morphotypes isolated from ants, their nest tunnels, and their transit areas are underrepresented. Chemical diversity between morphotypes of anthills and surroundings was different but was almost equally diverse (Figure 2A,B). Comparing the number of networks whose origin is related to anthills (60) and to their nearby area (57) revealed that the composition is balanced, which also occurs with solitary nodes, 387 and 389, respectively, and even the number of edges per node (Figure S8). In addition, the Venn diagram in Figure 2D reflects the same weight in terms of exclusive nodes. For these reasons, the selective study of fungi associated with anthills may be a valuable criterion for other investigations.

**FIGURE 3:**
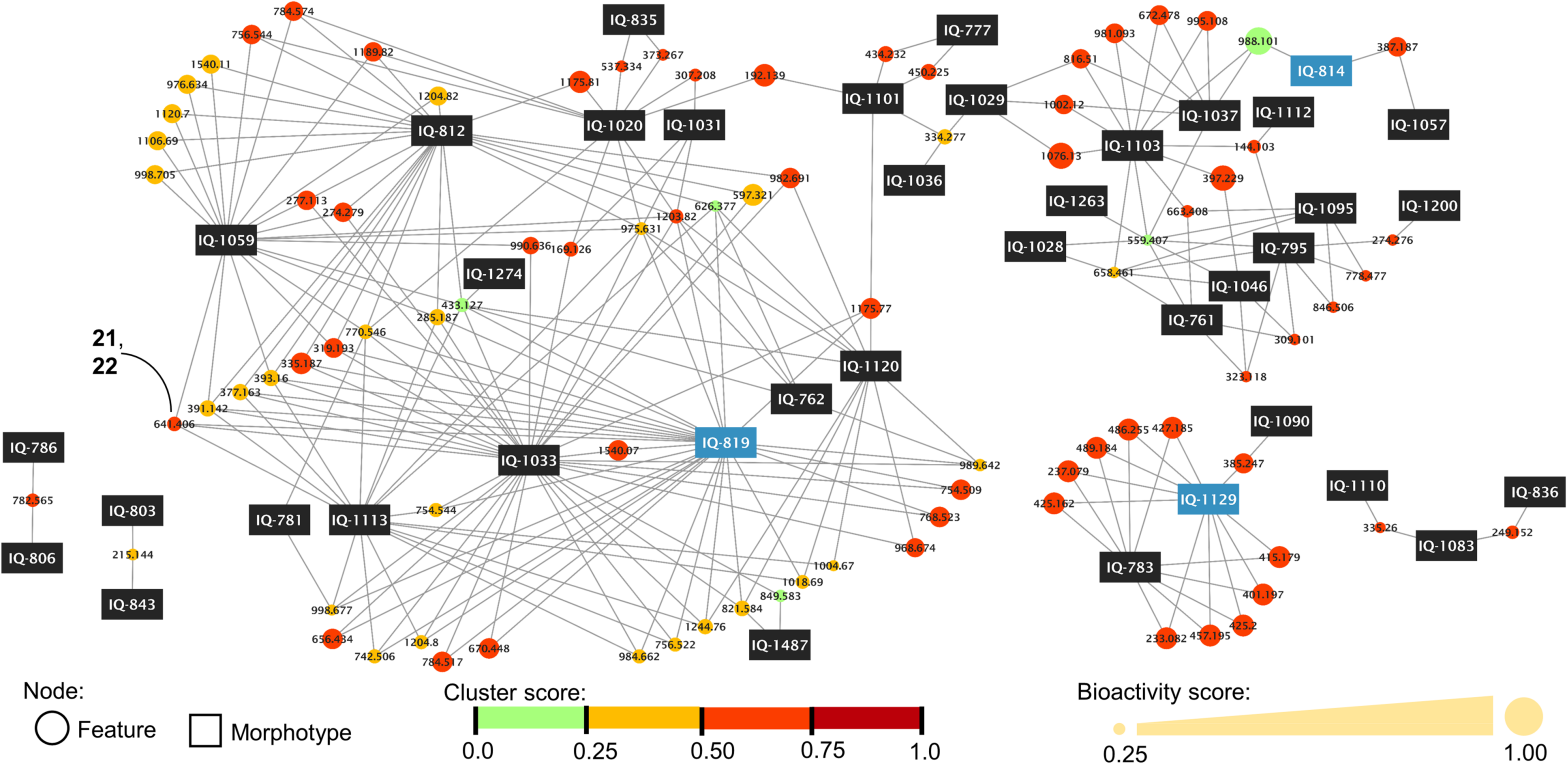
Compound activity mapping against *Acinetobacter baumannii* . This network has a clustering score > 0.1 (available value: −1 to 1) and an activity score > 0.25 (available value: 0–3). The blue square modes correspond to the morphotypes of interest. Isolated compounds were marked (ion detected [2M+H]^+^).

The molecular networks were unified with biological data through compound activity maps using NP Analyst platform (Figure 3) [25]. The analysis allowed to identify precursor ions of putative bioactive molecules. In the network, morphotypes are represented by square nodes and the precursor ions by circular nodes, joined together by edges. Furthermore, predictions are accompanied by two scores encoded in the size and color of the circular nodes; the size increases with the activity score, which measures the intensity of the predicted biological activity. The color of the nodes becomes darker as the clustering score increases; it measures the similarity of biological behavior between extracts.

Compound activity mapping was employed to identify potential candidates against *A. baumannii* . The generated network is composed of square nodes representing the morphotypes and circular nodes representing the presumed bioactive *m/z* ratios. In this instance, encompasses 90 *m*/*z* ratios, ranging from 121 to 1804 Da, distributed among 50 morphotypes (Figure 3). The predicted bioactivity, with a maximum clustering value of 0.73, is relatively low compared to the expected value of up to 3 for the dataset (Figure S21A). However, nodes with clustering values near zero exhibit a higher bioactivity score (~1.73; Figure S21B). This suggests that the most promising bioactive compounds within this mycobiota are not widely distributed but rather more specific to certain fungi. This insight helps predict the potential distribution of bioactivity among the constituents of the selected extracts.

**FIGURE 4:**
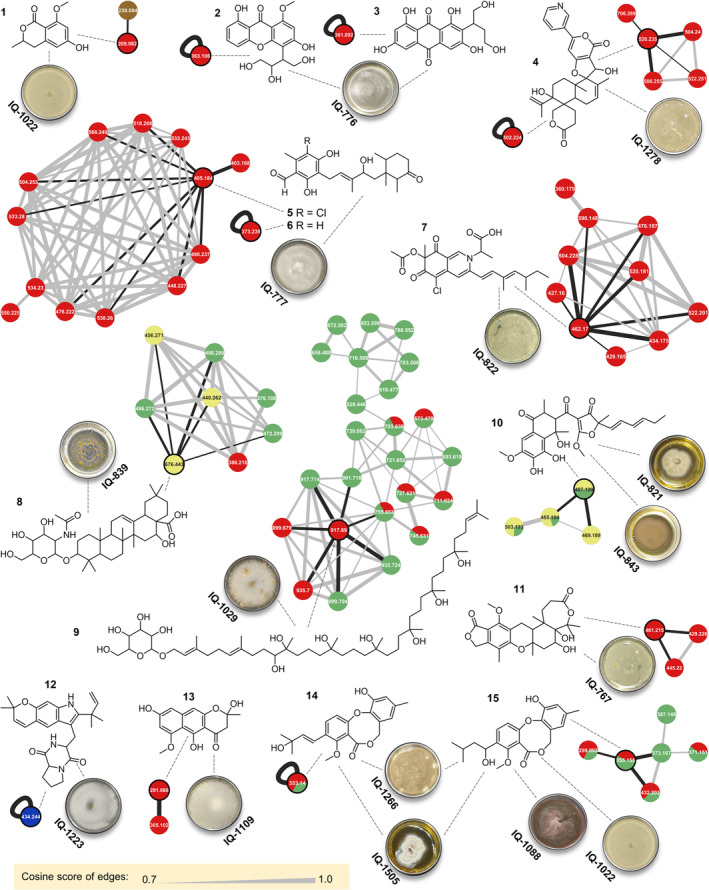
Dereplicated compounds and their associated nodes and molecular networks. The color of the nodes retains the code of Figure 2, each one contains the *m/z* ratio of the precursor ion, and the width of edges encodes the cosine of similarity. Dereplicated nodes are joined to the directly related ones by means of black edges. The image of the morphotypes corresponds to PD agar.

Dereplication analysis of the fungal extract collection identified 15 secondary metabolites from 16 fungi, mostly polyketides, alkaloids, and peptides whose structural core includes meroterpenes, xanthones, anthraquinones, naphthalenes, steroids, polyprenols, azaphilones, dipeptides, and coumarins, to mention a few examples. Pyrenocin A (**1**), secosterigmatocystin (**2**), versiconol (**3**), oxalicine B (**4**), deacetylchloronectrin (**5**), LL‐Z 1272 ε (**6**), azaphilone **7**, oleanane triterpenoid **8**, SCH 60059 (**9**), cyclic polyketide **10**, austalide **11**, asperversiamide F (**12**), fonsecin (**13**), 1′,2′‐dehydropenicillide (**14**), and vermixocin A (**15**) were detected, which makes their corresponding morphotypes candidates to find structural derivatives. However, these isolates were discarded because they are a promising source of known compounds (Figure 4 and Table S9). Interestingly, sometimes related structures are not part of the same network, such as **2** and **3** or **14** and **15**; this is due to defects in the network analysis caused by the GNPS algorithm, although to a greater extent it is explained by the application of strict construction parameters or poor spectrometric acquisitions, in terms of fragmentation; given the structural diversity in a large network, these drawbacks can be solved to a limited extent.

### 3.4 Chemical Study

**FIGURE 5:**
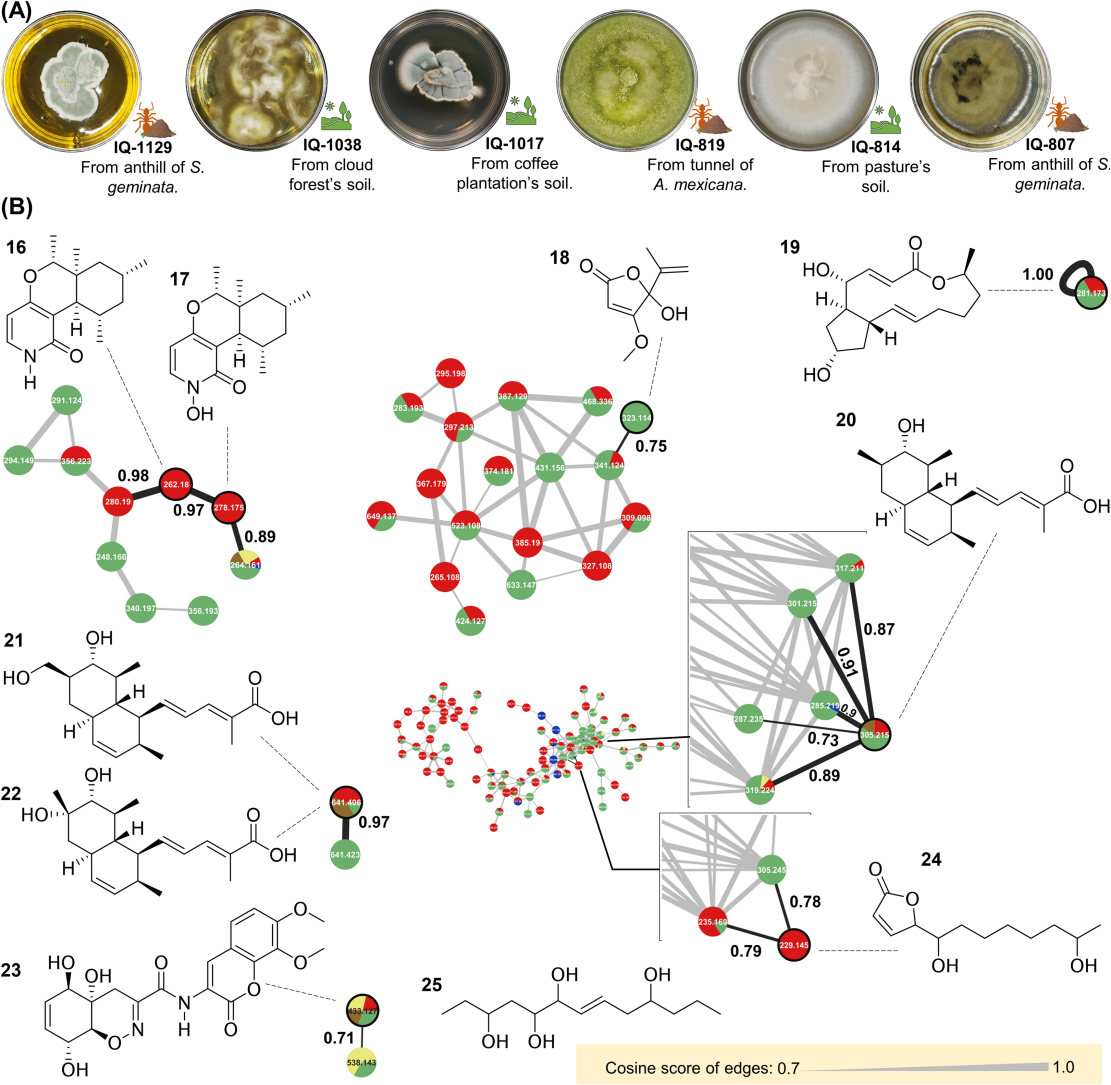
Morphotypes under chemical study and their isolation point. The morphotypes were grown on PD agar (A). Isolated compounds and their associated node in molecular networks. The color of the nodes retains the code of Figure 2, each node contains the *m/z* ratio of the precursor ion, and the edges vary in width according to the cosine similarity. In certain cases, the cosine value is indicated (B).

Certain bioactive fungi were prioritized according to the level of bioactivity against *A. baumannii* , the complexity of their chemical composition, the potentially bioactive features, and the feasibility of obtaining their extracts. Three of the selected morphotypes came from nest samples and three from soil (Figure 5A and Table S10). To reduce the chemical complexity of the extracts (Table S12) and focus on the isolation of bioactive compounds in a range of polarity, the growth inhibitory activity of the total extracts and their primary fractions towards *A. baumannii* was determined (Table S13). Variations in the effect of the extracts and their fractions were observed; however, in all cases, the isolation of majority constituents was achieved from primary fractions of medium polarity (Section S14). Surprisingly, IQ‐1038 extract lost the expected bioactivity when scaled up, but it was recovered after consecutive fractionations; bioactivity loss by extracts may result, for example, from antagonistic interactions among constituents [73]. This procedure led to the isolation of deoxy‐PF1140 (**16**) and PF1140 (**17**) from *Ascomycete* IQ‐1129 (*Eurotiomycetes*, *Aspergillaceae*); penicillic acid (**18**) from *Ascomycete* IQ‐1038; brefeldin A (**19**) from *Ascomycete* IQ‐1017; trichodermic acid (**20**), trichodermic acid A (**21**) and C (**22**) as well as trichodermamide A (**23**) from *Trichoderma* sp. IQ‐819 (*Sordariomycetes*, *Hypocreaceae*); *iso*‐cladospolide B (**24**) from *Clonostachys* sp. IQ‐814 (*Sordariomycetes*, *Bionectriaceae*); and finally (*E*)‐tridec‐7‐ene‐3,5,6,10‐tetraol (**25**) from *Cladosporium* sp. IQ‐807 (*Dothideomycetes*, *Cladosporiaceae*; Figure 5B and Sections S15–S20).

Compound **17** was originally isolated from a *Eupenicillium* species and patented following its broad antifungal effect towards a variety of *Candida* species and other fungi [74]. It was also isolated from a *Penicillium* species, and it was bioactive against ash dieback ( *Hymenoscyphus fraxineus* ) but phytotoxic [75, 76]. It was reported to inhibit the development of *Mycoplasma genitalium* and that its mechanism of action involves oxidative stress of an intracellular complex of **17** with iron (Fe^3+^) [77]. Its dehydroxylated derivative **16** was reported from a *Penicillium* of marine origin [78]. Compound **18** is a widely known mycotoxin along with other small lactones. It is an isolated constituent of many fungal species, commonly *Penicillium*, its cytotoxic, and antimicrobial activity is known, but it is an undesirable metabolite given its toxicity against mammals [79, 80, 81]. Lactone **19** was originally isolated from the soil fungus *Penicillium decumbens* in 1958; it has antifungal, antiviral, phytotoxic, and cytotoxic activity [82, 83]. Compounds **20**–**22** are commonly isolated from *Trichoderma* species. The first mention of **20** was made in 2012; since then, a variety of octahydronaphthalenes have been isolated [84, 85, 86, 87]. **20** has been reported as a weak cytotoxic agent against several cancer cell lines [86, 87] and has stood out for its significant inhibition of the phytopathogen *Botrytis cinera* [85]. Cyclic dipeptide **23** was described along with other structural derivatives from *Penicillium janthinellum* . Structurally, trichodermamides have a rare oxazadecalin backbone [88]. Lactone **24** was isolated from the tissue of a marine sponge and a few years later from the fungus *Cladosporium tenuissimum* [89, 90]. Its inhibitory activity against the alveolar adenocarcinoma cell line A564 was described; also, its α‐glucosidase inhibitory activity [91, 92, 93, 94].

**FIGURE 6:**
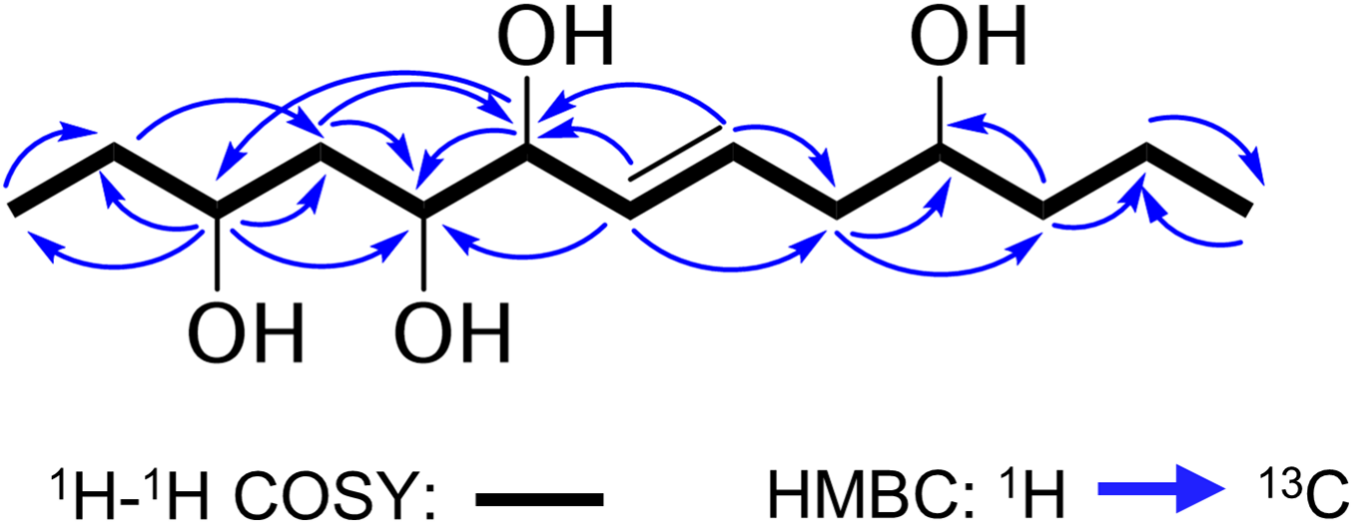
Key 2D NMR correlations of compound **25**. Spectra available in Section S19.

Compound **25** is a linear unsaturated polyol first reported in this work, which was isolated from a fungus *Clonostachys*, a genus characterized by synthesize polyketides and terpenoids [95]. This compound would give rise to a new chemical group in the genus, as the related polyols are glycitols or polyterpenoids. There are no records on the biological activity of structurally similar compounds [95]. To elucidate compound **25**, a molecular formula C_13_H_26_O_4_, with a degree of unsaturation, was determined from the relation *m/z* of adduct ion [M + H‐H_2_O]^+^ in HRMS spectra. Analysis of the ^13^C and ^1^H NMR and HSQC spectra revealed the presence of two methyl groups, five methylenes, and six methines. According to their chemical shifts, two of the methine groups are linked to a sp^2^ carbons of a double bond in *trans* position (*δ*_C_/*δ*_H_ 131.9/5.5 and 130.1/5.7, *J* = 15.3 Hz), whereas the rest of resonances belong to four oxymethines (δ_C_ 79.0, 76.8, 85.3, and 70.8). Because the molecule has only one spin system, its connectivity was established by analysis of the COSY and HMBC spectra (Figure 6). The relative configuration was established based on NOESY correlations and analysis of the coupling constants. Spectra available in Section S19.

In the molecular networks, the nodes corresponding with the isolated compounds (Figure 5B) support the structural similarity of **16** and **17**, but the values of the cosine of similarity (> 0.7) with their neighboring nodes reveal two close structural derivatives. Likewise, the nodes of **18**, **20**, and **24**, from which structurally close molecules, can also be expected to be isolated from other fungi. However, the rest of the compounds are mostly part of small networks and, consequently, are exclusive structures of the corresponding fungi. In the case of **25**, which does not appear in the network, it is thought that the analysis conditions made its ionization difficult, and the signal was filtered as noise in the analysis process.

The node closest to **16** with a ratio *m/z* 280.19 and a cosine of similarity of 0.98 corresponds to the ion [M + H]^+^ of a derivative with the molecular formula C_16_H_26_NO_3_ (3.6 ppm of error), and the signals of both compounds have a different retention times. This derivative is hypothesized to be deoxyakantomycin, whose degradation from **16** was described by De Silva et al. [78]. Furthermore, the ion with a ratio of *m/z* 264.161 close to **17** is present in many more morphotypes, but it is not an adduct generated in the spectrometric analysis and presents a loss of 14,014 Da, attributable to the loss of a methylene group, and the [M + H]^+^ ion of the derivative would have a molecular formula C_15_H_24_NO_3_ (4.9 ppm error for IQ‐1039 fungi). However, the literature search did not find matches for reported molecules classifiable as pyridones. This node and others that make up the network are a possible opportunity for the description of additional chemicals in IQ‐1129.

### 3.5 Antibacterial Activity of the Compounds

From the evaluation of the isolated compounds, it was determined that **16**, **17**, and **18** inhibit the growth of *A. baumannii* more than 50% at a concentration of 100 μg/mL (Table 1). Two bioactive compounds were isolated from IQ‐1129 and one from IQ‐1038; however, other compounds could contribute to the effect exhibited by the extract; the first fungus was isolated from a *S. geminata* nest and the second from its surroundings. Once the identity of the compounds was established, **18** was excluded from further determinations, as it is a known cytotoxic agent [80].

**TABLE 1:** In vitro inhibition of *Acinetobacter baumannii* growth by isolated compound.

Compounds by morphotype	Inhibition percentage	Concentration (mM)
*Ascomycete* IQ‐1129		
Deoxy‐PF1140 (**16**)	99.92 ± 0.66	0.38
PF1140 (**17**)	101.23 ± 1.71	0.36
*Ascomycete* IQ‐1038		
Penicillic acid (**18**)	67.75 ± 4.91	0.59
*Ascomycete* IQ‐1017		
Brefeldin A (**19**)	9.89 ± 3.37	0.36
*Trichoderma* sp. IQ‐819		
Trichodermic acid (**20**)	0	0.33
Trichodermic acid A (**21**)	5.36 ± 3.93	0.31
Trichodermic acid C (**22**)	0	0.31
Trichodermamide A (**23**)	0	0.23
*Cladosporium* sp. IQ‐807		
*iso*‐Cladospolide B (**24**)	6.68 ± 4.96	0.44
*Clonostachys* sp. IQ‐814		
(*E*)‐Tridec‐7‐ene‐3,5,6,10‐tetraol (**25**)	0	0.44
Colistin in water^a^	99.50 ± 0.83	0.02
Gentamicin in water^b^	29.97 ± 4.05	0.11
Negative control	0	
Sterility control	99.93 ± 0.11	

*Note:* Experiments carried out at least in quintuplicate, the average, and its standard deviation are presented. Samples were dissolved in dimethylsulfoxide and evaluated at a concentration of 100 μg/mL unless otherwise indicated.

^a^ Positive control evaluated at 20 μg/mL.

^b^ Positive control evaluated at 64 μg/mL.

**FIGURE 7:**
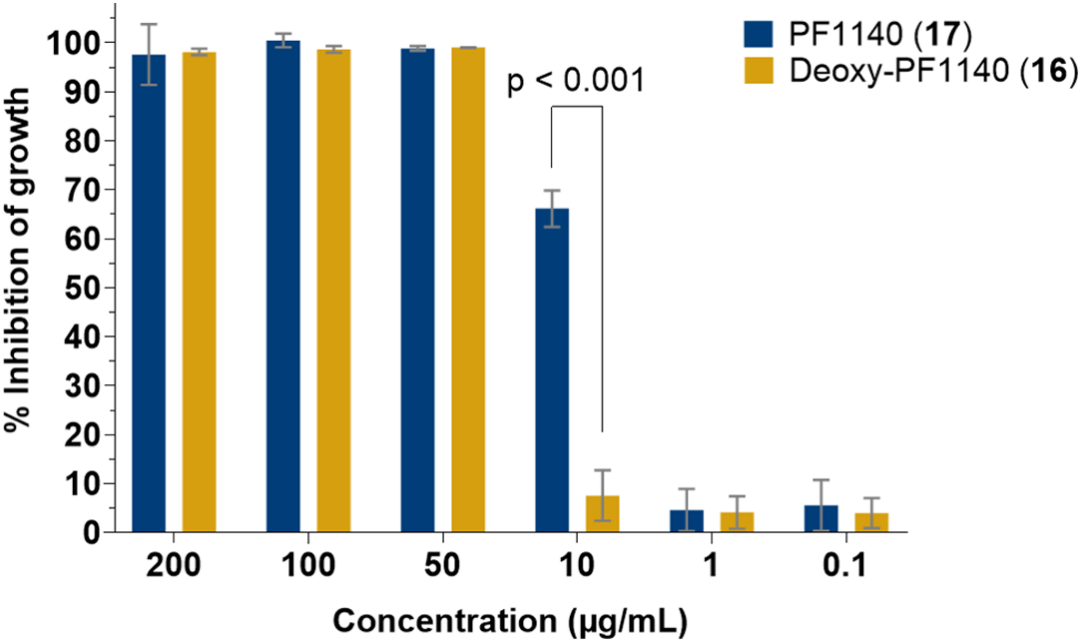
Inhibition of *Acinetobacter baumannii* growth by **16** and **17** at different concentrations. Compounds were dissolved in DMSO. Positive control of colistin in water at 20 μg/mL inhibited growth by 99.57 ± 0.50%. Statistically significant differences are indicated (Student's *t*‐test, *p* < 0.05).

The MIC of compounds **17** and **16** towards *A. baumannii* was determined to be 50 μg/mL (191.30 and 180.27  μM, respectively; Figure 7). Additionally, as the MBC for both compounds was > 200 μg/mL, a bacteriostatic effect is inferred at the inhibitory concentrations. At a concentration of 10 μg/mL, **17** showed a significantly inhibitory effect (66.17 ± 3.72%) compared to **16**. The different susceptibility of the pathogen to the compounds may be related to the capacity of **17** to form hydrogen bonds through its hydroxy group, assuming that the chelating capacity of compound is involved in the mechanism of action, as has been published for *M. genitalium* ; chelation is an important part of the mode of action of several antibacterial agents [77, 96]. This report is the first mention of their antibacterial effect against multidrug‐resistant bacteria like *A. baumannii* strain A564. The bioactivity associated with other structurally related molecules to **16** and **17** highlight the importance of studying tricyclic *N*‐hydroxy‐, *N*‐alkoxy, and *N*‐methoxy pyridin‐2‐ones [97, 98, 99, 100, 101]. In this sense, continuing the search for pyridin‐2‐ones in IQ‐1129, exploring the mechanism of action and establishing their toxicity are perspectives that extend to the future.

Compound activity mapping (Figure 7 and Figure S21) predicted the effect of the *m/z* ratios of **16** and **19**–**25**, but the remaining ones (**17** and **18**) were false negatives; it is worth mentioning that the range of the predicted effect does not align with the minimum and maximum possible value for the bioactivity score. It has been described that including fractions, bioactivity in the analysis improves the result, but that was not necessary [25, 26]. For the purposes of this project, its usefulness was to facilitate the prioritization of bioactive fungi and proved to be a viable strategy when trying to identify the candidates with the highest possible bioactivity; it is important to mention that its application requires careful manual review of the considered nodes.

## Conflicts of Interest

The authors declare no conflicts of interest.

## Supporting information


**Data S1** Supporting Information.

## Data Availability

The data are available free of charge upon request, including molecular networks and compound activity mapping. HPLC‐QTOF‐HRMS2 data of metabolomics analysis are available at MassIVE (accession number MSV000094490).
