# Supplementary material for: Harnessing Molecular and Bioactivity Network Analysis to Prioritize Antibacterial Compound Isolation From Ant‐Associated Fungi
Source: Phytochem Anal. 2025 Jan 30;36(5):1351–66. doi: 10.1002/pca.3513 (PMC12212023; doi:10.1002/pca.3513)
Supplement: Supplementary file 1 — Data S1 Supporting Information. [file PCA-36-1351-s001.pdf]

SUPPORTING INFORMATION

Harnessing Molecular and Bioactivity Network Analysis to Prioritize Antibacterial Compound Isolation from Ant-Associated Fungi

Ángel S. Aguilar-Colorad,[1] *Jesús Morales-Jiménez*[2] *and José A. Rivera-Chávez* [1]*

[1]Departamento de Productos Naturales, Instituto de Química, Universidad Nacional Autónoma de México, Ciudad Universitaria, Circuito Exterior s/n, 04510 Ciudad de México, México.

[2]Departamento El Hombre y su Ambiente, Universidad Autónoma Metropolitana, Calzada del Hueso 1100, 04690 Ciudad de México, México.

CORRESPONDING AUTHOR

*Phone: +52 55 5622-4450; Email: jrivera@iquimica.unam.mx

ORCID

Ángel Sahid Aguilar-Colorado: orcid.org/0000-0001-8411-503X

Jesús Morales-Jiménez: orcid.org/0000-0002-9010-6268

José Rivera-Chávez: orcid.org/0000-0002-9225-6200

CONTENTS

S1. Location of sampled nests. 1
S2. Origin of viable fungal morphotypes in the collection. 2
S3. Information about screened morphotypes. 4
S4. About the extracts of the screened morphotypes 11
S5. Bioactivity of screened morphotypes. 18
S6. Antimicrobial activity of the collection by isolation source. 25
S7. Molecular network of screened morphotypes. 27
S8. Distribution of edges by nodes in the global molecular network 30
S9. Dereplicated compounds. 31
S10. The morphology of the prioritized fungi. 34
S11. Accession numbers in GenBank for the prioritized fungi. 35
S12. Extracts of prioritized morphotypes. 36
S13. Bioactivity of prioritized morphotypes. 37
S14. Purification of compounds 39
IQ-1129 39
IQ-1038 39
IQ-1017 39
IQ-819 39
IQ-814 40
IQ-807 40
S15. Spectroscopic and spectrometric data of compounds isolated from IQ-1129 42
Deoxy-PF1140 (**16**) 42
PF1140 (**17**) 44
S16. Spectroscopic and spectrometric data of compounds isolated from IQ-1038 46
Penicillic acid (**18**) 46
S17. Spectroscopic and spectrometric data of compounds isolated from IQ-1017 48
Brefeldin A (**19**) 48
S18. Spectroscopic and spectrometric data of compounds isolated from IQ-819 50
Trichodermic acid (**20**) 50
Trichodermic acid A (**21**) 52
Trichodermic acid C (**22**) 54
Trichodermamide A (**23**) 56
S19. Spectroscopic and spectrometric data of compounds isolated from IQ-814 58
(*E*)-tridec-7-ene-3,5,6,10-tetraol (**25**) 58
S20. Spectroscopic and spectrometric data of compounds isolated from IQ-807 63
iso-Cladospolide B (**24**) 63
S21. Scatter plot of the bioactive features in NPAnalyst analysis 66
S22. References 67

S1. Location of sampled nests.

Location of sampled nests/ants and associated type of land

Nest/ Ant	Date of collection[*]	Latitude	Longitude	Altitude[⁑]	Locality and County[⁂]	Type of land	Ant species
1	June 9	19.3659342	-96.9787431	1101.13	El Águila Ranch, Teocelo	Pasture	*Solenopsis geminata*
2	June 9	19.3657226	-96.9789610	1101.44	El Águila Ranch, Teocelo	Pasture	*S. geminata*
3	June 9	19.3649590	-96.9793366	1113.51	El Águila Ranch, Teocelo	Pasture	*Dorymyrmex bicolor*
4	June 9	19.3649749	-96.9801952	1116.42	El Águila Ranch, Teocelo	Pasture	*S. geminata*
5	June 9	19.3548544	-96.9791197	1146.00	Los Magueyes Plantation, Teocelo	Coffee plantation	*S. geminata*
6	June 9	19.3545986	-96.9785548	1155.93	Los Magueyes Plantation, Teocelo	Coffee plantation	*S. geminata*
7	June 10	19.5129456	-96.9379752	1308.49	Santuario del Bosque de Niebla, Xalapa	Cloud forest reserve	*S. geminata*
8[†]	June 10	19.5123786	-96.9354510	1330.57	Santuario del Bosque de Niebla, Xalapa	Cloud forest reserve	*Nomamyrmex esenbeckii*
9	June 11	19.4590705	-96.9398001	1190.86	La Orduña, Coatepec	Rural	*Atta mexicana*
10	June 11	19.4551456	-96.9391602	1191.69	La Orduña, Coatepec	Rural	*A. mexicana*
11	June 11	19.4512825	-96.9304366	1153.25	La Orduña, Coatepec	Rural	*A. mexicana*
12[†]	June 12	19.4540761	-96.9906414	1233.82	El Trianón Farm, Coatepec	Coffee plantation	*Cheliomyrmex morosus*
13	June 12	19.4540652	-96.9904835	1236.74	El Trianón Farm, Coatepec	Coffee plantation	*S. geminata*
14	June 12	19.4531033	-96.9921013	1266.64	El Trianón Farm, Coatepec	Coffee plantation	*A. mexicana*
15	June 12	19.4545780	-96.9936207	1275.64	El Trianón Farm, Coatepec	Coffee plantation	*A. mexicana*
16	June 13	19.5190472	-96.9417664	1399.23	El Haya Ecological Park, Xalapa	Suburban	*S. geminata*
17	June 14	19.4545534	-96.9935866	1295.57	El Trianón Farm, Coatepec	Coffee plantation	*A. mexicana*
18[‡]	June 11	19.4518047	-96.9320550	1134.07	La Orduña, Coatepec	Rural	*Camponotus sericeiventris*

* Year 2021.
⁑ Meters above sea level.
⁂ In Veracruz, México.
† This species is an army ant; soil was collected from the transit area.
‡ This species is a carpenter ant, so only specimens were collected.

S2. Origin of viable fungal morphotypes in the collection.

Different viable filamentous morphotypes isolated from ground's samples

Ant species	Type of land	Locality	Nest	Origin of the soil		
				Anthill or transit area	Tunnel	Surroundings
S. geminata	Pasture	El Águila Ranch	2	20		18
			4	19		17
			1	19		23
	Coffee plantation	Los Magueyes Plantation	5	32		34
			6	30		34
		El Trianón Farm	13	23		20
	Cloud forest reserve	Santuario del Bosque de Niebla	7	13		14
	Ecological park	El Haya Ecological Park	16	21		21
				Subtotal: 177		Subtotal 181
A. mexicana	Agricultural road	La Orduña	9	23		15
			10	17		15
			11	14		13
	Coffee plantation	El Trianón Farm	14	8	7	19
			15	16		11
			17	12	20	12
				Subtotal: 90	Subtotal: 27	Subtotal: 85
D. bicolor	Pasture	El Águila Ranch	3	12		21
N. esenbeckii	Cloud forest reserve	Santuario del Bosque de Niebla	8	20		18
C. morosus	Coffee plantation	El Trianón Farm	12	20		15

A total of 666 morphotypes were recorded: 279 from an anthill, 40 from transit area, 27 from the tunnels and 320 from surroundings. Additionally, 35 morphotypes could not be isolated by reseeding and 57 lost viability under growth conditions.

Different viable filamentous morphotypes isolated from ants' samples

Ant species	Type of land	Locality	Ant	Morphotypes
A. mexicana	Agricultural road	La Orduña	14	4
			17	4
	Coffee plantation	El Trianón Farm	9	2
			10	12
				Subtotal: 22
C. sericeiventris	Agricultural road	La Orduña	18	4
N. esenbeckii	Santuario del Bosque de Niebla	Santuario del Bosque de Niebla	8	1
C. morosus	Coffee plantation	El Trianón Farm	12	1

A total of 28 morphotypes were recorded. Additionally, 2 lost viability under growth conditions.

S3. Information about screened morphotypes.

Morphotypes randomly selected from the collection

Count	Morphotype (IQ-)	Nest/ant	Ant species	Type of land	Origin of the sample
1	751	3	*D. bicolor*	Pasture	Anthill
2	752	14	*A. mexicana*	Coffee plantation	Tunnel
3	753	3	*D. bicolor*	Pasture	Surrounding
4	754	5	*S. geminata*	Coffee plantation	Anthill
5	755	2	*S. geminata*	Pasture	Surrounding
6	756	9	*A. mexicana*	Rural	Anthill
7	757	17	*A. mexicana*	Coffee plantation	Surrounding
8	758	15	*A. mexicana*	Coffee plantation	Anthill
9	759	9	*A. mexicana*	Rural	Surrounding
10	760	10	*A. mexicana*	Rural	Surrounding
11	761	4	*S. geminata*	Pasture	Surrounding
12	762	17	*A. mexicana*	Coffee plantation	Tunnel
13	763	5	*S. geminata*	Coffee plantation	Surrounding
14	764	13	*S. geminata*	Coffee plantation	Surrounding
15	766	8	*N. esenbeckii*	Cloud forest reserve	Transit area
16	767	4	*S. geminata*	Pasture	Anthill
17	768	1	*S. geminata*	Pasture	Anthill
18	769	7	*S. geminata*	Cloud forest reserve	Anthill
19	772	1	*S. geminata*	Pasture	Surrounding
20	773	14	*A. mexicana*	Coffee plantation	Surrounding
21	774	6	*S. geminata*	Coffee plantation	Anthill
22	776	13	*S. geminata*	Coffee plantation	Anthill
23	777	11	*A. mexicana*	Rural	Anthill
24	778	15	*A. mexicana*	Coffee plantation	Surrounding
25	779	16	*S. geminata*	Suburban	Anthill
26	780	17	*A. mexicana*	Coffee plantation	Anthill
27	781	10	*A. mexicana*	Rural	Anthill
28	782	6	*S. geminata*	Coffee plantation	Surrounding
29	783	12	*C. morosus*	Coffee plantation	Surrounding
30	784	7	*S. geminata*	Cloud forest reserve	Surrounding
31	785	3	*D. bicolor*	Pasture	Anthill
32	786	3	*D. bicolor*	Pasture	Surrounding
33	787	5	*S. geminata*	Coffee plantation	Anthill
34	788	9	*A. mexicana*	Rural	Anthill
35	789	17	*A. mexicana*	Coffee plantation	Surrounding
36	790	15	*A. mexicana*	Coffee plantation	Anthill
37	793	17	*A. mexicana*	Coffee plantation	Tunnel

38	794	5	*S. geminata*	Coffee plantation	Surrounding
39	795	13	*S. geminata*	Coffee plantation	Surrounding
40	796	8	*N. esenbeckii*	Cloud forest reserve	Transit area
41	797	4	*S. geminata*	Pasture	Anthill
42	799	12	*C. morosus*	Coffee plantation	Transit area
43	800	11	*A. mexicana*	Rural	Surrounding
44	801	1	*S. geminata*	Pasture	Surrounding
45	802	14	*A. mexicana*	Coffee plantation	Surrounding
46	803	6	*S. geminata*	Coffee plantation	Anthill
47	806	11	*A. mexicana*	Rural	Anthill
48	807	16	*S. geminata*	Suburban	Anthill
49	808	17	*A. mexicana*	Coffee plantation	Anthill
50	809	10	*A. mexicana*	Rural	Anthill
51	810	6	*S. geminata*	Coffee plantation	Surrounding
52	811	12	*C. morosus*	Coffee plantation	Surrounding
53	812	7	*S. geminata*	Cloud forest reserve	Surrounding
54	814	3	*D. bicolor*	Pasture	Surrounding
55	816	9	*A. mexicana*	Rural	Anthill
56	817	17	*A. mexicana*	Coffee plantation	Surrounding
57	819	17	*A. mexicana*	Coffee plantation	Tunnel
58	820	13	*S. geminata*	Coffee plantation	Surrounding
59	821	8	*N. esenbeckii*	Cloud forest reserve	Transit area
60	822	4	*S. geminata*	Pasture	Anthill
61	823	7	*S. geminata*	Cloud forest reserve	Anthill
62	824	12	*C. morosus*	Coffee plantation	Transit area
63	825	14	*A. mexicana*	Coffee plantation	Surrounding
64	826	6	*S. geminata*	Coffee plantation	Anthill
65	827	13	*S. geminata*	Coffee plantation	Anthill
66	829	6	*S. geminata*	Coffee plantation	Surrounding
67	830	12	*C. morosus*	Coffee plantation	Surrounding
68	832	3	*D. bicolor*	Pasture	Surrounding
69	835	10	*A. mexicana*	Rural	Surrounding
70	836	17	*A. mexicana*	Coffee plantation	Tunnel
71	837	13	*S. geminata*	Coffee plantation	Surrounding
72	838	8	*N. esenbeckii*	Cloud forest reserve	Transit area
73	839	12	*C. morosus*	Coffee plantation	Transit area
74	840	6	*S. geminata*	Coffee plantation	Anthill
75	841	13	*S. geminata*	Coffee plantation	Anthill
76	842	11	*A. mexicana*	Rural	Anthill
77	843	6	*S. geminata*	Coffee plantation	Surrounding
78	844	3	*D. bicolor*	Pasture	Anthill

79	846	9	*A. mexicana*	Rural	Anthill
80	847	10	*A. mexicana*	Rural	Surrounding
81	917	15	*A. mexicana*	Coffee plantation	Surrounding
82	1014	10	*A. mexicana*	Rural	Surrounding
83	1015	4	*S. geminata*	Pasture	Surrounding
84	1016	5	*S. geminata*	Coffee plantation	Surrounding
85	1017	13	*S. geminata*	Coffee plantation	Surrounding
86	1018	8	*N. esenbeckii*	Cloud forest reserve	Surrounding
87	1019	14	*A. mexicana*	Coffee plantation	Anthill
88	1020	8	*N. esenbeckii*	Cloud forest reserve	Transit area
89	1021	4	*S. geminata*	Pasture	Anthill
90	1022	1	*S. geminata*	Pasture	Anthill
91	1023	7	*S. geminata*	Cloud forest reserve	Anthill
92	1024	12	*C. morosus*	Coffee plantation	Transit area
93	1025	11	*A. mexicana*	Rural	Surrounding
94	1026	1	*S. geminata*	Pasture	Surrounding
95	1027	14	*A. mexicana*	Coffee plantation	Surrounding
96	1028	6	*S. geminata*	Coffee plantation	Anthill
97	1029	2	*S. geminata*	Pasture	Anthill
98	1030	13	*S. geminata*	Coffee plantation	Anthill
99	1031	11	*A. mexicana*	Rural	Anthill
100	1033	16	*S. geminata*	Suburban	Anthill
101	1035	10	*A. mexicana*	Rural	Anthill
102	1036	6	*S. geminata*	Coffee plantation	Surrounding
103	1037	12	*C. morosus*	Coffee plantation	Surrounding
104	1038	7	*S. geminata*	Cloud forest reserve	Surrounding
105	1039	5	*S. geminata*	Coffee plantation	Anthill
106	1041	9	*A. mexicana*	Rural	Anthill
107	1042	16	*S. geminata*	Suburban	Surrounding
108	1043	15	*A. mexicana*	Coffee plantation	Anthill
109	1044	9	*A. mexicana*	Rural	Surrounding
110	1045	10	*A. mexicana*	Rural	Surrounding
111	1046	4	*S. geminata*	Pasture	Surrounding
112	1047	5	*S. geminata*	Coffee plantation	Surrounding
113	1049	8	*N. esenbeckii*	Cloud forest reserve	Surrounding
114	1050	8	*N. esenbeckii*	Cloud forest reserve	Transit area
115	1052	1	*S. geminata*	Pasture	Anthill
116	1053	7	*S. geminata*	Cloud forest reserve	Anthill
117	1054	12	*C. morosus*	Coffee plantation	Transit area
118	1055	11	*A. mexicana*	Rural	Surrounding
119	1057	14	*A. mexicana*	Coffee plantation	Surrounding

120	1058	6	*S. geminata*	Coffee plantation	Anthill
121	1059	2	*S. geminata*	Pasture	Anthill
122	1060	13	*S. geminata*	Coffee plantation	Anthill
123	1061	11	*A. mexicana*	Rural	Anthill
124	1062	15	*A. mexicana*	Coffee plantation	Surrounding
125	1063	16	*S. geminata*	Suburban	Anthill
126	1064	10	*A. mexicana*	Rural	Anthill
127	1065	6	*S. geminata*	Coffee plantation	Surrounding
128	1066	12	*C. morosus*	Coffee plantation	Surrounding
129	1067	5	*S. geminata*	Coffee plantation	Anthill
130	1068	2	*S. geminata*	Pasture	Surrounding
131	1069	9	*A. mexicana*	Rural	Anthill
132	1072	10	*A. mexicana*	Rural	Surrounding
133	1073	4	*S. geminata*	Pasture	Surrounding
134	1074	5	*S. geminata*	Coffee plantation	Surrounding
135	1075	13	*S. geminata*	Coffee plantation	Surrounding
136	1076	8	*N. esenbeckii*	Cloud forest reserve	Surrounding
137	1077	8	*N. esenbeckii*	Cloud forest reserve	Transit area
138	1078	4	*S. geminata*	Pasture	Anthill
139	1079	1	*S. geminata*	Pasture	Anthill
140	1080	12	*C. morosus*	Coffee plantation	Transit area
141	1081	1	*S. geminata*	Pasture	Surrounding
142	1082	14	*A. mexicana*	Coffee plantation	Surrounding
143	1083	6	*S. geminata*	Coffee plantation	Anthill
144	1084	2	*S. geminata*	Pasture	Anthill
145	1085	13	*S. geminata*	Coffee plantation	Anthill
146	1086	16	*S. geminata*	Suburban	Anthill
147	1087	10	*A. mexicana*	Rural	Anthill
148	1088	6	*S. geminata*	Coffee plantation	Surrounding
149	1090	5	*S. geminata*	Coffee plantation	Anthill
150	1091	2	*S. geminata*	Pasture	Surrounding
151	1095	10	*A. mexicana*	Rural	Surrounding
152	1097	5	*S. geminata*	Coffee plantation	Surrounding
153	1098	13	*S. geminata*	Coffee plantation	Surrounding
154	1099	8	*N. esenbeckii*	Cloud forest reserve	Surrounding
155	1101	4	*S. geminata*	Pasture	Anthill
156	1102	12	*C. morosus*	Coffee plantation	Transit area
157	1103	1	*S. geminata*	Pasture	Surrounding
158	1104	14	*A. mexicana*	Coffee plantation	Surrounding
159	1106	2	*S. geminata*	Pasture	Anthill
160	1108	16	*S. geminata*	Suburban	Anthill

161	1109	10	*A. mexicana*	Rural	Anthill
162	1110	6	*S. geminata*	Coffee plantation	Surrounding
163	1111	12	*C. morosus*	Coffee plantation	Surrounding
164	1112	5	*S. geminata*	Coffee plantation	Anthill
165	1113	2	*S. geminata*	Pasture	Surrounding
166	1114	9	*A. mexicana*	Rural	Anthill
167	1115	16	*S. geminata*	Suburban	Surrounding
168	1117	4	*S. geminata*	Pasture	Surrounding
169	1118	5	*S. geminata*	Coffee plantation	Surrounding
170	1119	13	*S. geminata*	Coffee plantation	Surrounding
171	1120	8	*N. esenbeckii*	Cloud forest reserve	Surrounding
172	1121	8	*N. esenbeckii*	Cloud forest reserve	Transit area
173	1124	1	*S. geminata*	Pasture	Surrounding
174	1127	2	*S. geminata*	Pasture	Anthill
175	1128	13	*S. geminata*	Coffee plantation	Anthill
176	1129	16	*S. geminata*	Suburban	Anthill
177	1130	10	*A. mexicana*	Rural	Anthill
178	1131	6	*S. geminata*	Coffee plantation	Surrounding
179	1132	12	*C. morosus*	Coffee plantation	Surrounding
180	1133	5	*S. geminata*	Coffee plantation	Anthill
181	1180	6	*S. geminata*	Coffee plantation	Surrounding
182	1197	14	*A. mexicana*	Coffee plantation	Ant
183	1198	17	*A. mexicana*	Coffee plantation	Ant
184	1199	17	*A. mexicana*	Coffee plantation	Ant
185	1200	10	*A. mexicana*	Rural	Ant
186	1202	18	*C. sericeiventris*	Rural	Ant
187	1203	10	*A. mexicana*	Rural	Ant
188	1204	9	*A. mexicana*	Rural	Ant
189	1205	9	*A. mexicana*	Rural	Ant
190	1206	14	*A. mexicana*	Coffee plantation	Ant
191	1208	8	*N. esenbeckii*	Cloud forest reserve	Ant
192	1210	10	*A. mexicana*	Rural	Ant
193	1211	10	*A. mexicana*	Rural	Ant
194	1213	10	*A. mexicana*	Rural	Ant
195	1215	10	*A. mexicana*	Rural	Ant
196	1216	18	*C. sericeiventris*	Rural	Ant
197	1217	17	*A. mexicana*	Coffee plantation	Ant
198	1218	14	*A. mexicana*	Coffee plantation	Ant
199	1219	10	*A. mexicana*	Rural	Ant
200	1220	14	*A. mexicana*	Coffee plantation	Ant
201	1221	10	*A. mexicana*	Rural	Ant

202	1222	10	*A. mexicana*	Rural	Ant
203	1223	18	*C. sericeiventris*	Rural	Ant
204	1224	18	*C. sericeiventris*	Rural	Ant
205	1253	3	*D. bicolor*	Pasture	Anthill
206	1254	14	*A. mexicana*	Coffee plantation	Tunnel
207	1255	3	*D. bicolor*	Pasture	Surrounding
208	1256	5	*S. geminata*	Coffee plantation	Anthill
209	1258	9	*A. mexicana*	Rural	Anthill
210	1259	16	*S. geminata*	Suburban	Surrounding
211	1260	17	*A. mexicana*	Coffee plantation	Surrounding
212	1261	15	*A. mexicana*	Coffee plantation	Anthill
213	1262	9	*A. mexicana*	Rural	Surrounding
214	1263	10	*A. mexicana*	Rural	Surrounding
215	1265	17	*A. mexicana*	Coffee plantation	Tunnel
216	1266	5	*S. geminata*	Coffee plantation	Surrounding
217	1268	8	*N. esenbeckii*	Cloud forest reserve	Surrounding
218	1269	14	*A. mexicana*	Coffee plantation	Anthill
219	1271	4	*S. geminata*	Pasture	Anthill
220	1272	1	*S. geminata*	Pasture	Anthill
221	1273	7	*S. geminata*	Cloud forest reserve	Anthill
222	1274	12	*C. morosus*	Coffee plantation	Transit area
223	1275	11	*A. mexicana*	Rural	Surrounding
224	1276	1	*S. geminata*	Pasture	Surrounding
225	1277	14	*A. mexicana*	Coffee plantation	Surrounding
226	1278	6	*S. geminata*	Coffee plantation	Anthill
227	1279	2	*S. geminata*	Pasture	Anthill
228	1280	13	*S. geminata*	Coffee plantation	Anthill
229	1282	15	*A. mexicana*	Coffee plantation	Surrounding
230	1284	17	*A. mexicana*	Coffee plantation	Anthill
231	1290	3	*D. bicolor*	Pasture	Surrounding
232	1292	2	*S. geminata*	Pasture	Surrounding
233	1296	9	*A. mexicana*	Rural	Surrounding
234	1299	17	*A. mexicana*	Coffee plantation	Tunnel
235	1303	14	*A. mexicana*	Coffee plantation	Anthill
236	1309	11	*A. mexicana*	Rural	Surrounding
237	1322	7	*S. geminata*	Cloud forest reserve	Surrounding
238	1426	17	*A. mexicana*	Coffee plantation	Anthill
239	1435	5	*S. geminata*	Coffee plantation	Surrounding
240	1436	8	*N. esenbeckii*	Cloud forest reserve	Surrounding
241	1438	1	*S. geminata*	Pasture	Anthill
242	1453	7	*S. geminata*	Cloud forest reserve	Surrounding

243	1456	15	*A. mexicana*	Coffee plantation	Anthill
244	1464	15	*A. mexicana*	Coffee plantation	Anthill
245	1479	17	*A. mexicana*	Coffee plantation	Ant
246	1480	10	*A. mexicana*	Rural	Ant
247	1487	9	*A. mexicana*	Rural	Surrounding
248	1505	5	*S. geminata*	Coffee plantation	Anthill

22 additional morphotypes were considered but did not grow on the substrate used.

S4. About the extracts of the screened morphotypes

Small-scale cultivation of the screened morphotypes

Count	Morphotype (IQ-)	Incubation days in broth	Incubation days in cereal	Yield (mg extract/g cereal)
1	751	13	24	27.33
2	752	13	24	59.00
3	753	13	24	64.23
4	754	13	24	21.50
5	755	13	24	77.57
6	756	13	24	30.23
7	757	13	24	33.93
8	758	13	24	17.60
9	759	13	24	47.93
10	760	13	24	15.27
11	761	13	24	23.07
12	762	13	24	17.87
13	763	13	24	24.87
14	764	13	24	37.07
15	766	13	24	28.93
16	767	13	24	82.67
17	768	13	24	62.00
18	769	13	24	19.63
19	772	13	24	106.07
20	773	13	24	19.83
21	774	13	24	42.27
22	776	13	25	32.87
23	777	13	25	31.10
24	778	13	25	43.70
25	779	13	25	44.17
26	780	13	24	55.67
27	781	13	25	63.63
28	782	13	25	59.87
29	783	13	25	19.70
30	784	13	25	18.70
31	785	13	25	19.23
32	786	13	25	123.50
33	787	13	25	38.10
34	788	13	25	19.27
35	789	13	25	22.07
36	790	13	25	31.87
37	793	13	25	60.07

38	794	13	25	21.37
39	795	13	25	37.47
40	796	13	25	17.03
41	797	13	25	17.27
42	799	13	27	21.30
43	800	13	27	14.97
44	801	14	26	49.43
45	802	13	27	9.03
46	803	13	27	17.77
47	806	13	27	25.93
48	807	14	26	15.37
49	808	11	33	17.30
50	809	11	33	45.33
51	810	13	27	23.70
52	811	13	27	34.90
53	812	14	26	34.20
54	814	13	27	39.13
55	816	14	26	26.37
56	817	13	27	51.27
57	819	14	26	28.50
58	820	14	26	36.57
59	821	14	26	37.67
60	822	13	27	54.87
61	823	14	26	124.60
62	824	13	27	13.30
63	825	13	27	14.97
64	826	13	27	62.50
65	827	13	27	34.17
66	829	13	27	25.23
67	830	13	27	102.27
68	832	13	27	41.97
69	835	13	27	41.93
70	836	13	27	31.80
71	837	13	27	32.63
72	838	13	27	34.00
73	839	13	27	59.63
74	840	13	27	44.13
75	841	13	27	35.00
76	842	13	27	29.30
77	843	13	27	28.80
78	844	13	27	60.13

79	846	13	27	33.30
80	847	13	27	10.90
81	917	11	30	44.68
82	1014	10	29	102.00
83	1015	10	29	64.07
84	1016	10	29	35.17
85	1017	10	29	27.47
86	1018	10	29	14.17
87	1019	10	29	22.13
88	1020	10	29	20.80
89	1021	10	29	25.10
90	1022	10	29	32.50
91	1023	10	29	45.17
92	1024	10	29	158.33
93	1025	10	29	41.57
94	1026	10	29	156.90
95	1027	10	29	35.23
96	1028	10	29	34.23
97	1029	10	29	69.17
98	1030	10	29	20.53
99	1031	10	29	44.77
100	1033	10	29	36.60
101	1035	10	23	72.60
102	1036	10	23	55.03
103	1037	10	23	42.23
104	1038	10	23	37.23
105	1039	10	23	60.83
106	1041	10	23	16.03
107	1042	10	23	56.47
108	1043	10	23	9.57
109	1044	10	23	105.17
110	1045	10	23	23.33
111	1046	10	23	18.33
112	1047	10	23	21.20
113	1049	10	23	15.73
114	1050	10	23	21.17
115	1052	10	23	12.73
116	1053	10	23	34.97
117	1054	10	23	74.07
118	1055	10	23	15.07
119	1057	10	23	25.30

120	1058	10	23	15.40
121	1059	10	23	37.20
122	1060	6	23	31.53
123	1061	6	23	19.27
124	1062	6	23	22.27
125	1063	6	23	46.03
126	1064	6	23	82.60
127	1065	6	23	27.13
128	1066	6	23	13.37
129	1067	6	23	23.37
130	1068	6	23	20.80
131	1069	6	23	21.70
132	1072	6	23	33.20
133	1073	6	23	26.43
134	1074	6	23	32.70
135	1075	6	23	21.07
136	1076	6	23	17.57
137	1077	6	23	14.40
138	1078	6	23	37.77
139	1079	6	23	28.87
140	1080	6	23	21.50
141	1081	6	23	51.80
142	1082	6	23	72.73
143	1083	6	23	21.00
144	1084	6	23	37.70
145	1085	13	33	38.00
146	1086	13	33	29.47
147	1087	13	33	85.63
148	1088	13	33	39.33
149	1090	13	33	12.40
150	1091	13	33	34.17
151	1095	13	33	34.83
152	1097	13	33	18.30
153	1098	13	33	66.83
154	1099	13	33	10.40
155	1101	13	33	23.17
156	1102	13	33	51.93
157	1103	13	33	46.63
158	1104	13	33	18.00
159	1106	13	33	24.20
160	1108	13	33	19.83

161	1109	13	33	22.40
162	1110	13	33	12.63
163	1111	13	33	11.53
164	1112	13	33	13.09
165	1113	13	33	24.33
166	1114	13	33	19.93
167	1115	13	33	60.27
168	1117	13	33	40.73
169	1118	13	33	53.27
170	1119	13	33	12.90
171	1120	11	33	48.93
172	1121	11	19	47.33
173	1124	13	33	36.37
174	1127	13	33	42.83
175	1128	13	33	28.07
176	1129	13	33	25.47
177	1130	13	33	22.80
178	1131	13	33	16.40
179	1132	13	33	5.00
180	1133	13	33	5.23
181	1180	11	33	52.30
182	1197	10	27	29.33
183	1198	10	27	21.07
184	1199	10	27	36.00
185	1200	10	27	25.23
186	1202	10	27	37.53
187	1203	9	33	82.33
188	1204	10	27	23.43
189	1205	10	27	21.50
190	1206	11	33	35.10
191	1208	10	27	17.50
192	1210	10	27	33.10
193	1211	11	33	61.30
194	1213	11	33	15.73
195	1215	10	27	37.03
196	1216	10	27	23.50
197	1217	10	27	60.53
198	1218	10	27	33.47
199	1219	10	27	36.17
200	1220	10	27	19.37
201	1221	10	27	59.33

202	1222	10	27	52.97
203	1223	10	27	70.07
204	1224	10	27	70.63
205	1253	12	34	8.93
206	1254	12	34	38.33
207	1255	12	34	37.43
208	1256	12	34	9.83
209	1258	12	34	13.77
210	1259	12	34	23.77
211	1260	12	34	22.13
212	1261	12	34	10.40
213	1262	12	34	16.30
214	1263	12	34	52.03
215	1265	12	34	8.67
216	1266	12	34	17.80
217	1268	12	34	32.23
218	1269	12	34	12.47
219	1271	12	34	29.43
220	1272	12	34	22.77
221	1273	12	34	15.67
222	1274	12	34	22.83
223	1275	12	34	14.03
224	1276	12	34	16.73
225	1277	12	34	19.17
226	1278	12	34	19.80
227	1279	12	34	20.33
228	1280	12	34	49.10
229	1282	12	34	13.83
230	1284	11	33	20.77
231	1290	11	33	20.27
232	1292	11	33	27.87
233	1296	11	33	41.97
234	1299	11	25	2.90
235	1303	11	33	16.73
236	1309	11	33	43.87
237	1322	11	33	33.70
238	1426	11	33	6.37
239	1435	11	25	27.90
240	1436	11	33	72.63
241	1438	11	33	17.20
242	1453	11	33	22.40

243	1456	11	33	19.77
244	1464	11	33	36.37
245	1479	11	33	84.20
246	1480	11	33	41.80
247	1487	11	33	37.97
248	1505	11	25	37.33
Growth medium control*		10.92 ± 2.23	28.42 ± 4.36	46.83 ± 35.28

Morphotypes without growth in cereal are not included in this table.
Yield calculated from a 3 g of Nestlé Honey Cheerios® in a 50 mL Erlenmeyer flask or plastic tube inoculated with the morphotype grown in 5 mL potato-dextrose broth, without replicates.
* Extract of potato-dextrose broth and Nestlé Honey Cheerios®, its result corresponds to the average ± standard deviation of 12 independent culture medium.

S5. Bioactivity of screened morphotypes.

In vitro inhibition of bacterial growth by the selected morphotypes

Count	Morphotype (IQ-)	*Acinetobacter baumannii* A564	SD	*Klebsiella pneumoniae* K2	SD
1	751	19.01	15.99	4.42	4.33
2	752	0	0	13.99	5.25
3	753	8.73	6.35	0	0
4	754	16.11	4.61	0	0
5	755	3.12	2.81	13.99	12.13
6	756	17.6	2.83	0	0
7	757	14.18	4.42	0	0
8	758	0.94	0.82	19.19	6.74
9	759	10.93	5.6	0	0
10	760	6.26	8.03	1.33	2.3
11	761	14.32	6.48	0	0
12	762	3.7	3.2	25.31	0.27
13	763	47.1	2.14	0	0
14	764	0	0	1.74	3.02
15	766	6.41	2.89	0	0
16	767	17.34	2.73	0	0
17	768	31.65	2.72	0.19	0.33
18	769	2.34	2.49	0.73	1.27
19	772	1.88	3.26	0	0
20	773	11.49	1.75	0	0
21	774	13.11	1.33	0	0
22	776	7.51	7.21	0	0
23	777	25.56	6.86	0	0
24	778	5.66	4.9	0	0
25	779	12.94	3.47	0	0
26	780	43.25	1.34	3.29	5.64
27	781	15.84	4.82	30.14	9.48
28	782	14.2	7.15	0	0
29	783	12.43	9.97	0	0
30	784	24.81	9.65	0	0
31	785	11.51	1.2	0	0
32	786	20.56	4.18	12.53	8.27
33	787	0.68	0.71	6.17	5.77
34	788	5.28	5.22	0	0
35	789	0.78	0.99	0	0
36	790	13.4	11.62	0	0
37	793	11.24	9.77	0	0

38	794	26.61	3.71	14.67	7.85
39	795	35.29	8.8	0	0
40	796	5.56	8.5	0	0
41	797	32.52	5.39	0	0
42	799	10.04	2.7	0	0
43	800	0	0	25.15	3.48
44	801	3.61	1.95	2.69	4.55
45	802	4.12	7.14	0	0
46	803	32.35	7.38	0	0
47	806	33.76	8.43	0	0
48	807	25.12	3.49	0	0
49	808	12.64	7.61	0	0
50	809	13.91	4.3	4.73	3.36
51	810	2.15	2.78	0.44	0.77
52	811	16.9	1.97	0	0
53	812	45.56	1.11	0	0
54	814	66.71	3.8	0	0
55	816	16.49	1.5	0	0
56	817	8.34	6.01	0	0
57	819	42.43	4.22	0	0
58	820	7.33	8.76	2.86	2.93
59	821	20.79	8.33	0	0
60	822	0.46	0.46	0	0
61	823	24.41	1.38	18.38	15.34
62	824	9.86	3.87	1.31	2.28
63	825	2.7	2.44	26.03	7.81
64	826	1.99	2.33	0	0
65	827	9.72	1.67	3.29	5.18
66	829	25.01	3.37	0	0
67	830	8.68	5.75	7.1	12.3
68	832	20.05	6.93	0	0
69	835	18.78	1.57	12.71	2.08
70	836	22.45	2.61	8.42	2.32
71	837	23.23	1.7	0	0
72	838	2.32	3.79	24.9	2.5
73	839	10.19	5.9	0	0
74	840	6.22	2.67	0	0
75	841	6.25	10.83	0	0
76	842	19.34	5.27	0	0
77	843	28	9.14	0	0
78	844	3.23	5.59	26.23	0.41

79	846	21.19	6.2	1.7	2.94
80	847	34.42	5.53	0	0
81	917	16.73	3.75	0	0
82	1014	0	0	0	0
83	1015	0	0	1.03	1.78
84	1016	17.81	2.34	0	0
85	1017	11.55	0.43	21.81	2.32
86	1018	28.47	0.6	0	0
87	1019	24.31	3.2	0	0
88	1020	42.02	3.02	6.92	7.15
89	1021	6.76	5.88	22.22	5.14
90	1022	14.54	2.23	0	0
91	1023	1.33	1.72	0	0
92	1024	3.2	4.28	1.16	1.06
93	1025	11.2	6.49	0	0
94	1026	0	0	0	0
95	1027	14.32	2.99	0	0
96	1028	12.22	1.75	0	0
97	1029	39.69	7.56	0	0
98	1030	26.93	21.38	2.82	4.89
99	1031	22.15	3.67	18.21	16.78
100	1033	41.58	1.02	27.26	1.62
101	1035	13.92	4.11	8.02	8.44
102	1036	19.68	7.67	1.31	1.95
103	1037	25.48	10.18	0	0
104	1038	27.82	11.07	5.37	9.3
105	1039	13.54	4.7	0	0
106	1041	6.79	8.57	0.36	0.63
107	1042	15.7	9.56	0	0
108	1043	13.29	8.32	0	0
109	1044	16.23	2.3	23.84	3.74
110	1045	14.58	1.63	2.16	2.86
111	1046	38.06	6.35	0	0
112	1047	16.26	2.71	5.22	5.11
113	1049	4.53	5.8	1.43	2.48
114	1050	29.19	2.5	6.43	11.14
115	1052	9.56	1.83	0	0
116	1053	0	0	0	0
117	1054	20.88	8.97	3.65	3.29
118	1055	12.11	2.46	0.26	0.45
119	1057	14.68	2.99	0	0

120	1058	26.33	22.12	0	0
121	1059	35.33	0.94	0	0
122	1060	27.82	4.11	7.24	6.89
123	1061	33.1	2.48	0	0
124	1062	14.23	1.68	3.14	3.42
125	1063	21.23	4.87	0	0
126	1064	0.59	1.02	0	0
127	1065	10.66	7.31	0	0
128	1066	8.48	7.4	0.44	0.77
129	1067	19.95	7.98	0	0
130	1068	9.83	10.31	0	0
131	1069	0.31	0.53	0	0
132	1072	4.53	7.78	0	0
133	1073	11.39	8.27	0	0
134	1074	17.49	3.98	0	0
135	1075	4.65	4.9	0	0
136	1076	19.49	4.53	0	0
137	1077	1.77	3.06	0	0
138	1078	24.88	7.66	3.83	3.39
139	1079	41.5	6.26	18.4	10.42
140	1080	0	0	18.57	2.28
141	1081	14.62	12.8	0	0
142	1082	13.28	1.94	0	0
143	1083	42.81	7.85	2.78	1.33
144	1084	2.92	5.06	5.66	9.8
145	1085	18.96	1.14	8.1	5.11
146	1086	0	0	0	0
147	1087	8.39	4.67	3.99	6.04
148	1088	13.17	3.1	0	0
149	1090	9.23	8.5	22	5.47
150	1091	7.29	6.55	15.34	13.96
151	1095	24.01	5.08	29.09	8.76
152	1097	16.02	3.92	18.75	5.09
153	1098	7.86	10.91	13.56	8.42
154	1099	0.69	1.2	10.32	2.92
155	1101	46.63	5.7	0	0
156	1102	10.98	1.36	0	0
157	1103	56.03	3.25	0	0
158	1104	17.83	7.49	23.5	2.27
159	1106	14	5.4	10.82	4.24
160	1108	3.45	3.17	32.95	2.37

161	1109	10.96	2.89	2.85	4.93
162	1110	15.14	3.03	22.3	4.31
163	1111	5.64	4.15	1.04	1.51
164	1112	7.06	4.53	19.79	6.12
165	1113	19.13	3.5	32.01	4.29
166	1114	7.06	4.61	32.11	1.11
167	1115	4.97	4.74	4.16	7.2
168	1117	6.64	3.22	0	0
169	1118	0	0	4.84	4.3
170	1119	2.91	4.26	11.74	2.56
171	1120	39.41	2.52	3.51	3.43
172	1121	5.71	5.54	3.38	3.19
173	1124	31.33	1.02	0	0
174	1127	18.76	2.99	0.23	0.39
175	1128	15.85	2.79	8.09	7.2
176	1129	73.44	3.2	31.79	6.05
177	1130	0	0	5.01	4.39
178	1131	15.6	0.44	29.92	4.27
179	1132	0	0	0	0
180	1133	12.98	10.77	21.94	2.23
181	1180	18.06	4.49	1.9	2.86
182	1197	5.48	4.36	0	0
183	1198	4.01	5.66	0	0
184	1199	7.98	3.97	0	0
185	1200	23.16	5.76	0	0
186	1202	0	0	0	0
187	1203	1.45	2.52	4.56	6.47
188	1204	8.69	1.31	0	0
189	1205	9.73	4.83	0	0
190	1206	21.15	7.22	1.25	2.17
191	1208	26.03	10.17	0	0
192	1210	7.73	3.04	0	0
193	1211	0.03	0.05	33.11	23.14
194	1213	5.27	5.41	13.79	8.64
195	1215	12.78	1.26	0	0
196	1216	13.99	7.45	0.42	0.72
197	1217	7.45	2.04	0	0
198	1218	7.94	6.6	0	0
199	1219	2.63	3.3	0.02	0.03
200	1220	7.74	4.26	0	0
201	1221	4.8	1.98	1.03	1.33

202	1222	4.19	1.57	0	0
203	1223	0	0	0	0
204	1224	11.34	1.81	4.61	7.99
205	1253	2.78	4.82	0	0
206	1254	0	0	0	0
207	1255	4.56	3.32	0.12	0.2
208	1256	22.26	5.99	9.46	8.62
209	1258	6.28	6.42	30.29	1.1
210	1259	13.92	0.57	7.82	6.93
211	1260	0	0	8.38	3.08
212	1261	0	0	5.41	5.38
213	1262	3.04	3.86	12.93	3.36
214	1263	26.28	0.7	9.36	2.48
215	1265	0	0	0	0
216	1266	13.05	5.69	10.42	1.75
217	1268	15.22	5.87	0	0
218	1269	14.85	2.97	2.65	2.75
219	1271	4.37	2.74	0.23	0.39
220	1272	0	0	14.1	7.99
221	1273	0	0	13.52	10.75
222	1274	44.74	11.4	34.73	5.73
223	1275	0	0	27.89	5.8
224	1276	15.69	9.7	2.56	4.44
225	1277	8.96	3.47	21.72	1.68
226	1278	0.92	1.59	4.08	1.48
227	1279	4.18	4.21	24.18	0.95
228	1280	0	0	8.83	7.9
229	1282	5.3	2.96	23.39	4.87
230	1284	17.24	6.25	9.64	9.99
231	1290	1.51	2.61	0.48	0.83
232	1292	8.76	2.33	0	0
233	1296	3.15	3.07	0	0
234	1299	1.73	2.99	2.45	2.32
235	1303	3.05	2.65	0.52	0.9
236	1309	0	0	22.3	3.41
237	1322	10.89	9.43	24.6	2.43
238	1426	8.82	12.24	8.89	3.08
239	1435	8.94	2.2	7.41	8.89
240	1436	10.47	3.03	1.13	1.3
241	1438	6.08	5.78	1.28	2.22
242	1453	0	0	0	0

243	1456	0	0	3.51	3.15
244	1464	0.42	0.73	2.63	4.56
245	1479	4.11	7.11	0	0
246	1480	9.36	4.32	1.77	2.91
247	1487	20.86	7.87	0	0
248	1505	2.78	4.82	12.02	10.49
Gentamicin in DMSO*		-	-	99.17	1.23
Gentamicin in water*		22.67	1.55	-	-
Colistin in water⁑		98.01	0.40	-	-
Growth medium control⁂		3.27	2.53	4.82	6.60
Negative control		1.52	0.91	2.13	1.78
Sterility control		99.99	0.39	100.02	0.05

Experiment performed minimum in triplicate, the average, and its standard deviation (SD) are presented. Samples were dissolved in DMSO unless otherwise indicated and evaluated at a concentration of 250 µg/mL.

* Positive control, evaluated at a concentration of 64 and 8 µg/mL against *A. baumannii* and *K. pneumoniae*, respectively.

⁑ Positive control, evaluated at a concentration of 20 µg/mL

⁂ Extract of potato-dextrose broth and Nestlé Honey Cheerios®, its result corresponds to the average and standard deviation of 12 independent culture medium evaluated in triplicate against each bacteria.

S6. Antimicrobial activity of the collection by isolation source.

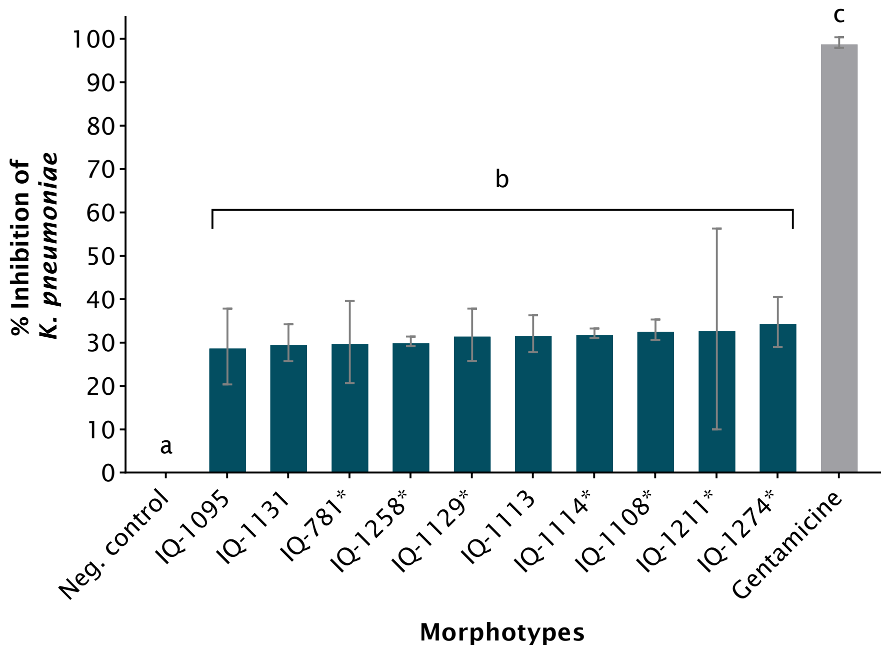

(A)

Top ten values against *K. pneumoniae* on bar charts. Morphotypes marked with an asterisk were isolated from a sample associated with ants or their nests; inhibition percentages with different lettering differ statistically (Welch's analysis of variance, $p < 0.05$; *post hoc* Games-Howell test at 95% confidence level; gentamicin evaluated at 8 µg/mL in DMSO).

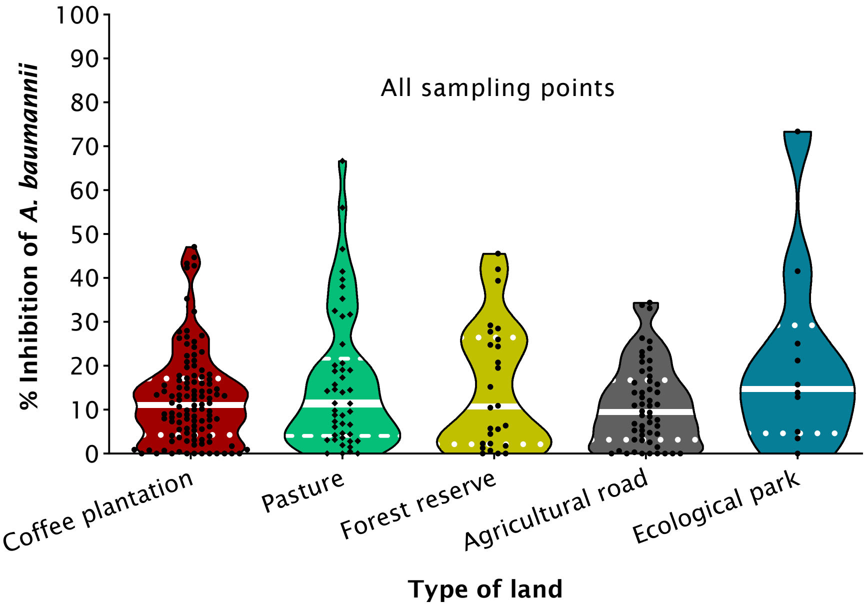

(B)

The violin plot of inhibition percentages of morphotypes by type of land against *A. baumannii*. The median is drawn with a solid line and quartiles with dotted lines. Medians by group do not differ statistically (Kruskal-Wallis test, $p = 0.53$).

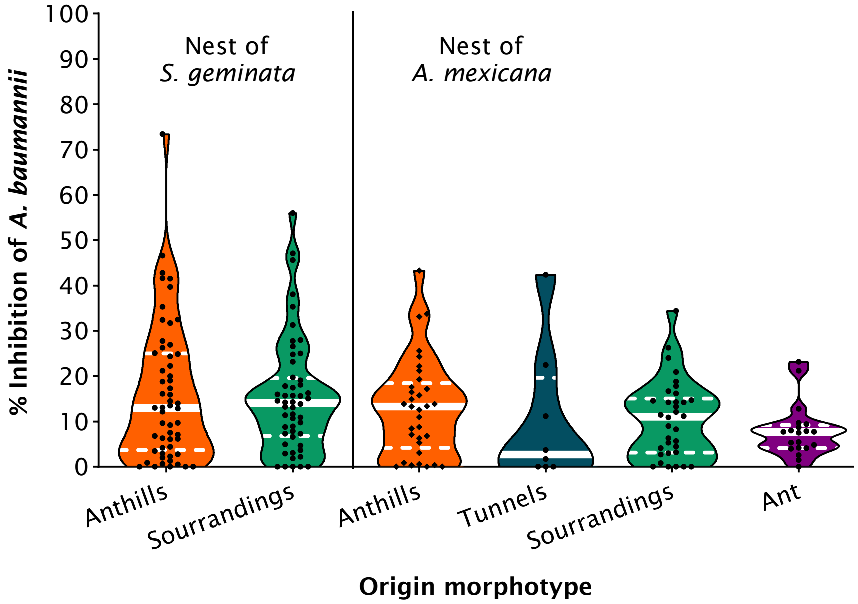

(C)

The violin plot of inhibition percentages of morphotypes by origin and the two species of ants best represented in the collection against *A. baumannii*. The median is drawn with a solid line and quartiles with dotted lines. Medians by group do not differ statistically (Kruskal-Wallis test, p = 0.09).

S7. Molecular network of screened morphotypes.

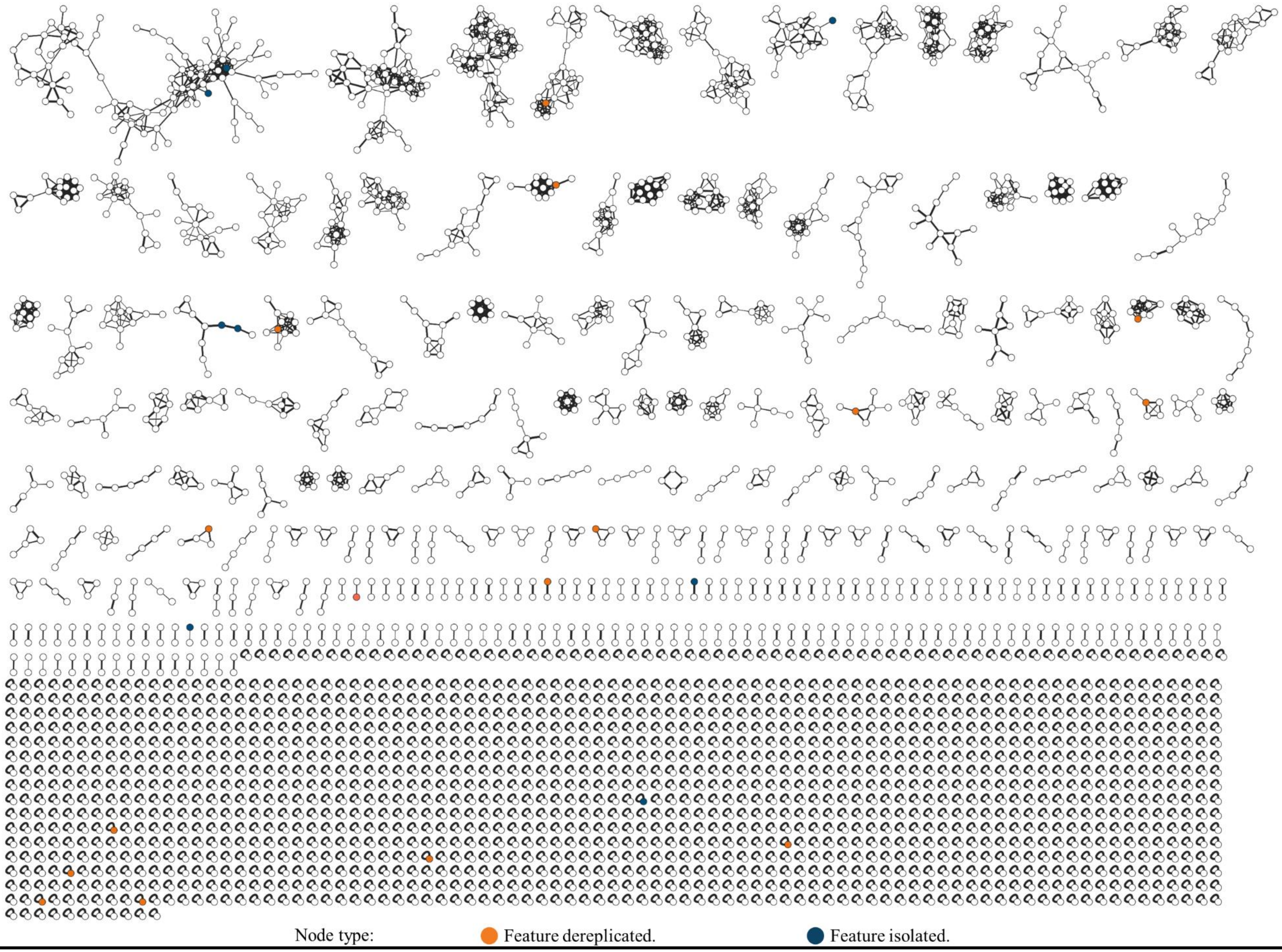

Dereplicated and isolated characteristics indicated in the global molecular network of bioprospected morphotype collection.

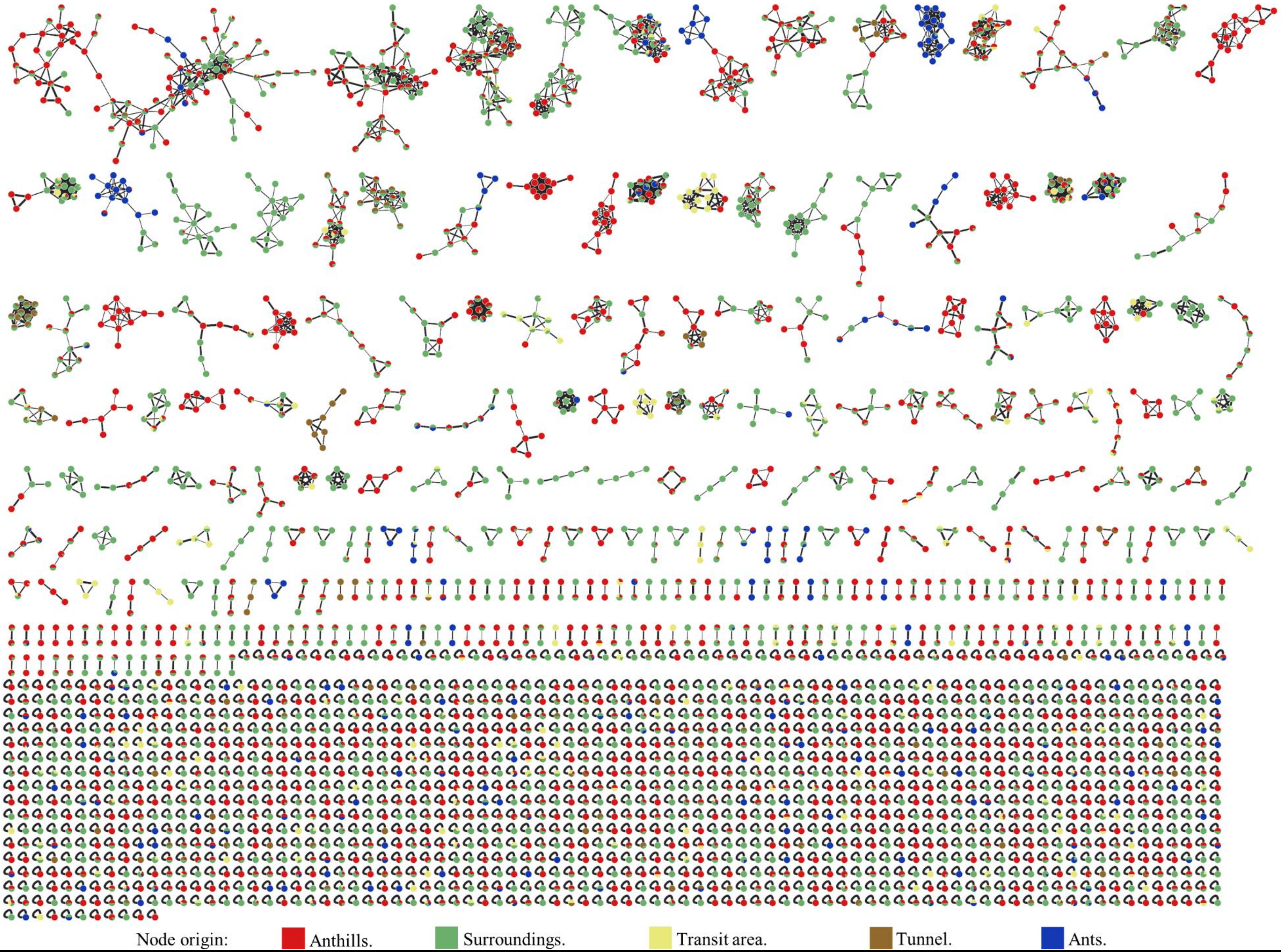

Global molecular network of the collection of bioprospected morphotypes according to isolate origin.

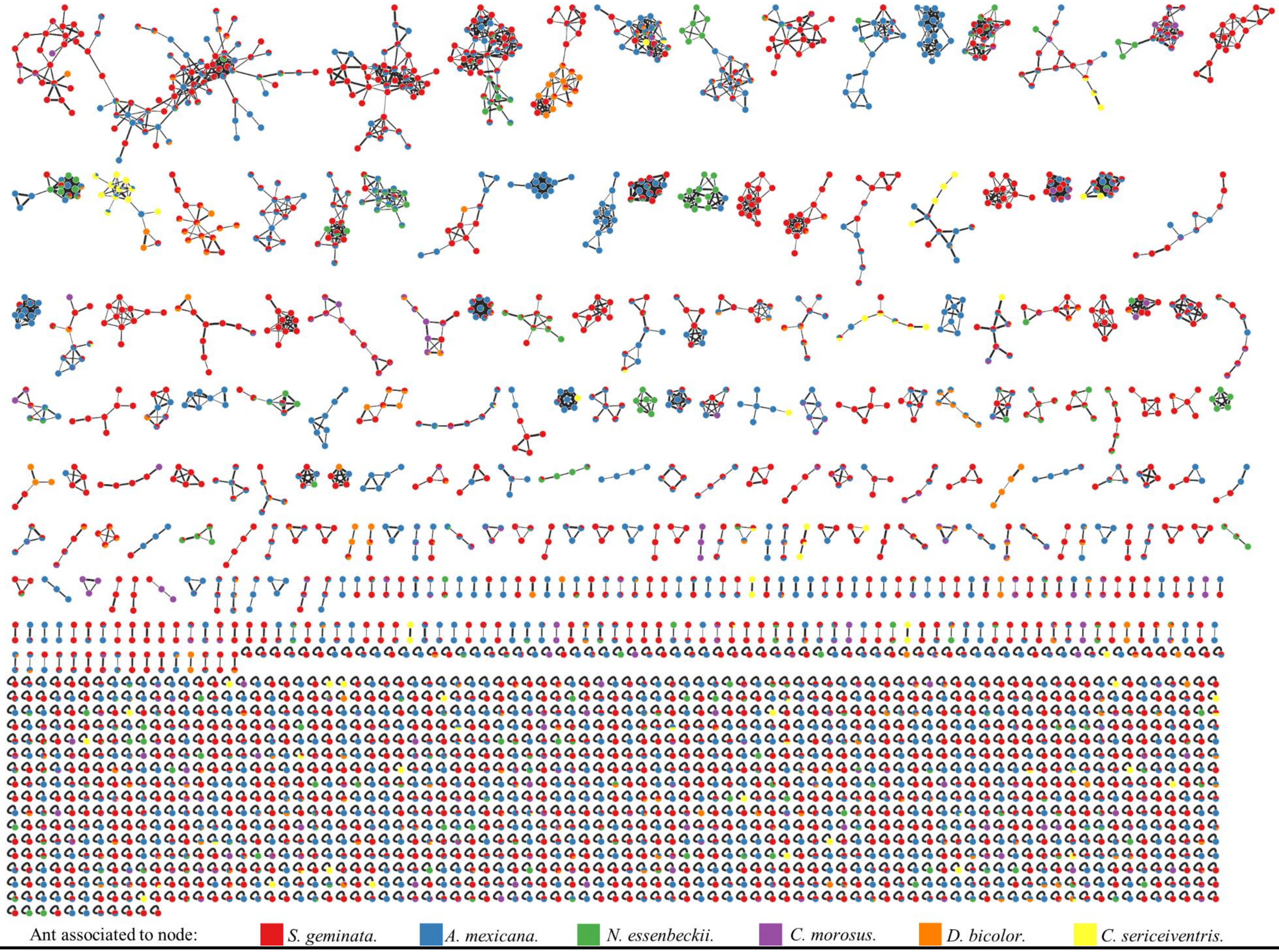

Global molecular network of the collection of bioprospected morphotypes according to the associated ant species

S8. Distribution of edges by nodes in the global molecular network

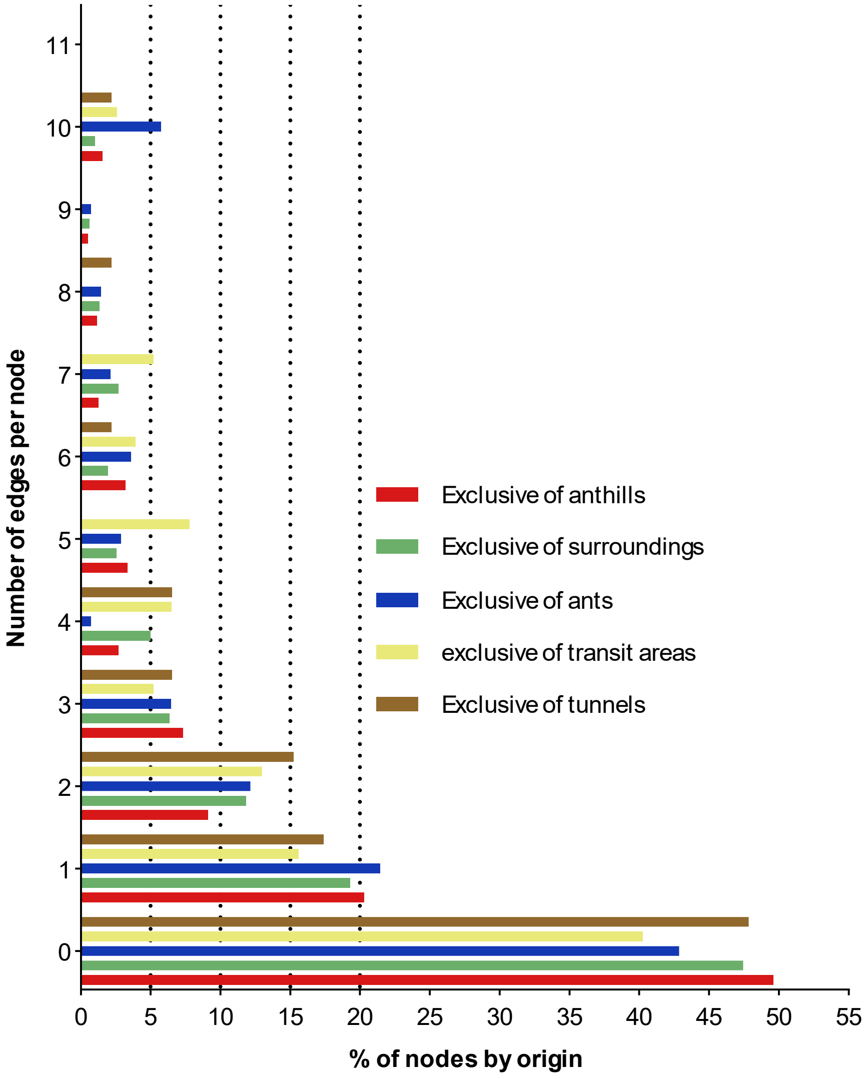

Considering the nodes exclusively related to a source in the global molecular network, the bar chart compares the percentage of nodes with the number of edges they have.

S9.Dereplicated compounds.

Information about dereplicated nodes

Node	Compound and molecular formula*	Precursor ion (*m/z*) *	Adduct*	Mass accuracy (ppm)*	Cosine similarity *	Matching peaks*	Associated morphotype*	Retention time (min)*	Exact mass theorical	Precursor ion (*m/z*)**	Mass accuracy (ppm)**	Difference of mass **
28151	Versiconol (**3**) $C_{18}H_{16}O_8$	361.092	$[M+H]^+$	0.0	0.8908	5	IQ-776	3.64	361.0923	361.0925	-0.6	-0.0002
28151							IQ-776	3.57	361.0923	361.0925	-0.6	-0.0002
29122	Asperversiamide F (**12**) $C_{26}H_{31}N_3O_3$	434.244	$[M+H]^+$	0.0	0.9665	11	IQ-1223	5.47	434.2443	434.2439	0.9	0.0004
29122							IQ-1223	5.39	434.2443	434.2439	0.9	0.0004
30927	Oleanane triterpenoid (**8**) $C_{38}H_{61}NO_9$	676.443	$[M+H]^+$	1.4	0.8909	8	IQ-839	4.87	676.4424	676.4431	-1.0	-0.0007
30927							IQ-839	4.79	676.4424	676.4431	-1.0	-0.0007
31807		917.690	$[M+H]^+$	2.2	0.7920	32	IQ-1029	4.59	917.6929	917.6896	3.6	0.0033
31807	SCH 60059 (**9**) $C_{51}H_{96}O_{13}$						IQ-1029	4.65	917.6929	917.6896	3.6	0.0033
31807							IQ-1029	4.72	917.6929	917.6896	3.6	0.0033
28170	Secosterigmatocystin (**2**) $C_{18}H_{18}O_8$	363.106	$[M+H]^+$	2.8	0.7738	7	IQ-776	2.77	363.1079	363.1061	5.0	0.0018
28170							IQ-776	2.84	363.1079	363.1061	5.0	0.0018
28170							IQ-776	2.90	363.1079	363.1061	5.0	0.0018
26677		291.086	$[M+H]^+$	3.5	0.8089	9	IQ-1109	3.44	291.0868	291.0860	2.7	0.0008
26677							IQ-1109	3.37	291.0868	291.0860	2.7	0.0008
26677	Fonsecin (**13**) $C_{15}H_{14}O_6$						IQ-1109	3.64	291.0868	291.0860	2.7	0.0008
26677							IQ-1109	3.58	291.0868	291.0860	2.7	0.0008
26677							IQ-1109	3.79	291.0868	291.0860	2.7	0.0008
26677							IQ-1109	3.51	291.0868	291.0860	2.7	0.0008
26677							IQ-1109	3.71	291.0868	291.0860	2.7	0.0008
29981	Oxalicine B (**4**) $C_{30}H_{33}NO_7$	520.235	$[M+H]^+$	3.9	0.8852	14	IQ-1278	3.86	520.2335	520.2355	-3.8	-0.002
29981							IQ-1278	3.71	520.2335	520.2355	-3.8	-0.002

29981							IQ-1278	3.78	520.2335	520.2355	-3.8	-0.002
29981							IQ-1278	3.64	520.2335	520.2355	-3.8	-0.002
29820	Oxalicine B (**4**) $C_{30}H_{33}NO_7$	502.224	$[M-H2O+H]^+$	4.0	0.9523	9	IQ-1278	3.73	502.2229	502.2243	-2.8	-0.0014
29820							IQ-1278	3.66	502.2229	502.2243	-2.8	-0.0014
29400	Derived from austalide J (**11**) $C_{25}H_{32}O_8$	461.215	$[M+H]^+$	4.4	0.7028	14	IQ-767	4.46	461.2175	461.2154	4.6	0.0021
29400							IQ-767	4.39	461.2175	461.2154	4.6	0.0021
29400							IQ-767	4.32	461.2175	461.2154	4.6	0.0021
29408	Azaphilone (**7**) $C_{24}H_{28}ClNO_6$	462.170	[M+H]+	4.4	0.8943	14	IQ-822	6.13	462.1683	462.1697	-3.0	-0.0014
29408							IQ-822	6.07	462.1683	462.1697	-3.0	-0.0014
24470	Pyrenocin A (**1**) $C_{11}H_{12}O_4$	209.082	$[M+H]^+$	4.8	0.7001	7	IQ-1022	2.43	209.0813	209.0816	-1.4	-0.0003
24470							IQ-1022	2.52	209.0813	209.0816	-1.4	-0.0003
24470							IQ-1022	2.38	209.0813	209.0816	-1.4	-0.0003
28790	Deacetylchloronectrin (**5**) $C_{23}H_{31}ClO_5$	405.184	$[M-H2O+H]^+$	4.9	0.9065	12	IQ-777	7.95	405.1832	405.1838	-1.5	-0.0006
28790							IQ-777	7.15	405.1832	405.1838	-1.5	-0.0006
28790							IQ-777	8.29	405.1832	405.1838	-1.5	-0.0006
28294	LL-Z 1272 ε (**6**) $C_{23}H_{32}O_4$	373.239	$[M+H]^+$	5.4	0.9821	10	IQ-777	6.93	373.2378	373.2387	-2.4	-0.0009
28294							IQ-777	7.00	373.2378	373.2387	-2.4	-0.0009
28039	Vermixocin A (**15**) $C_{21}H_{24}O_6$	355.155	$[M-H2O+H]^+$	5.6	0.9653	11	IQ-1505	6.59	355.1545	355.1563	-5.1	-0.0018
28039							IQ-1505	5.58	355.1545	355.1563	-5.1	-0.0018
28039							IQ-1505	6.54	355.1545	355.1563	-5.1	-0.0018
28039							IQ-1505	5.65	355.1545	355.1563	-5.1	-0.0018
28039							IQ-1505	5.52	355.1545	355.1563	-5.1	-0.0018
28039							IQ-1505	5.45	355.1545	355.1563	-5.1	-0.0018
28039							IQ-1022	5.54	355.1545	355.1542	0.8	0.0003
28039							IQ-1022	6.61	355.1545	355.1542	0.8	0.0003
28039							IQ-1266	5.46	355.1545	355.1561	-4.5	-0.0016

28039							IQ-1266	6.53	355.1545	355.1561	-4.5	-0.0016
28039							IQ-1266	5.59	355.1545	355.1561	-4.5	-0.0016
28039							IQ-1266	6.60	355.1545	355.1561	-4.5	-0.0016
28039							IQ-1266	5.53	355.1545	355.1561	-4.5	-0.0016
28039							IQ-1088	5.65	355.1545	355.1527	5.1	0.0018
28039							IQ-1088	5.92	355.1545	355.1527	5.1	0.0018
28039							IQ-1088	6.61	355.1545	355.1527	5.1	0.0018
28039							IQ-1088	5.59	355.1545	355.1527	5.1	0.0018
28039							IQ-1088	6.68	355.1545	355.1527	5.1	0.0018
28039							IQ-1088	5.52	355.1545	355.1527	5.1	0.0018
28014	1',2'-Dehydropenicillide (**14**) $C_{21}H_{22}O_6$	353.140	[M-H2O+H]$^+$	5.7	0.7758	8	IQ-1505	4.91	353.1389	353.1402	-3.7	-0.0013
28014							IQ-1505	4.85	353.1389	353.1402	-3.7	-0.0013
28014							IQ-1266	4.86	353.1389	353.1401	-3.4	-0.0012
29670	Cyclic polyketide (**10**) $C_{26}H_{30}O_9$	487.199	[M+H]$^+$	6.1	0.8874	12	IQ-843	4.80	487.1968	487.1985	-3.5	-0.0017
29670							IQ-843	5.25	487.1968	487.1985	-3.5	-0.0017
29670							IQ-821	4.80	487.1968	487.1991	-4.7	-0.0023
29670							IQ-821	5.26	487.1968	487.1991	-4.7	-0.0023

* According to the consensus spectrum comparison of the node with the GNPS library.
⁑ Data extracted from the total ion chromatogram of each extract.
⁂ Data calculated.
IUPAC name for some compounds:

8. 10-((3-acetamido-4,5-dihydroxy-6-(hydroxymethyl)tetrahydro-2H-pyran-2-yl)oxy)-5-hydroxy-2,2,6a,6b,9,9,12a-heptamethyl-1,3,4,5,6,6a,6b,7,8,8a,9,10,11,12,12a,12b,13,14b-octadecahydropicene-4a(2H)-carboxylic acid.

11. 5a,6-dihydroxy-13-methoxy-5,5,7a,9,14b-pentamethyl-1,2,5a,6,7,7a,10,14,14a,14b-decahydro-5H-furo[3,4-i]oxepino[4,3-a]xanthene-3,12-dione.

7. 2-(7-acetoxy-5-chloro-3-((1E,3E)-3,5-dimethylhepta-1,3-dien-1-yl)-7-methyl-6,8-dioxo-7,8-dihydroisoquinolin-2(6H)-yl)propanoic acid.

10. 2-((1E,3E)-hexa-1,3-dien-1-yl)-5-methoxy-2-methyl-4-(1,7,8-trihydroxy-6-methoxy-1,3-dimethyl-4-oxo-1,2,3,4-tetrahydronaphthalene-2-carbonyl)furan-3(2H)-one.

S10.The morphology of the prioritized fungi.

Photographs of prioritized morphotypes

On potato-dextrose agar*	On cereal⁑
**IQ-1129**	
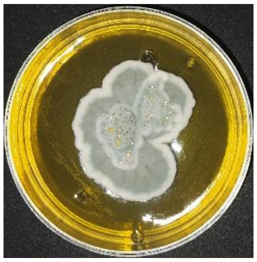	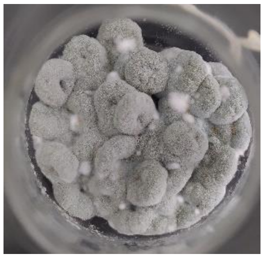
**IQ-1038**	
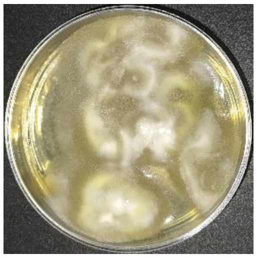	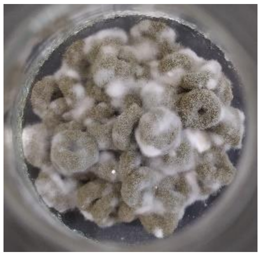
**IQ-819**	
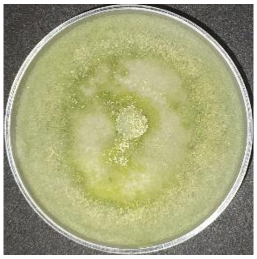	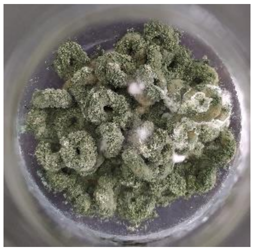
**IQ-814**	
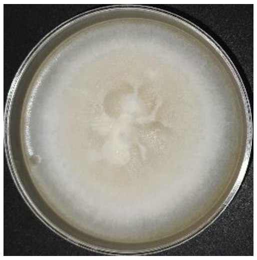	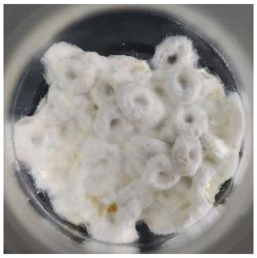
**IQ-807**	
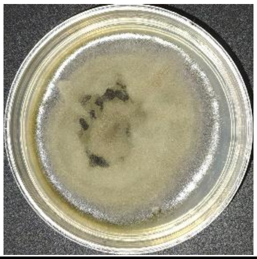	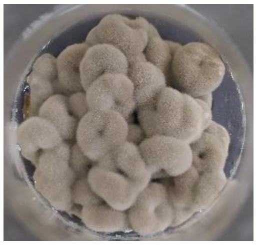

* Potato-dextrose agar in 5 cm diameter Petri dishes.
⁑ 9 grams of Nestlé Honey Cheerios® in a 150 ml Erlenmeyer flask inoculated with the morphotype grown in 15 mL potato-dextrose broth.

S11. Accession numbers in GenBank for the prioritized fungi.

Region ITS of the prioritized fungi

Fungi	GenBank accession number
IQ-807	
IQ-814	
IQ-819	

S12. Extracts of prioritized morphotypes.

Medium-scale cultivation of the prioritized morphotypes

Morphotype	Number of growth mediums*	Incubation days in broth	Incubation days in cereal	Amount of extract (g)
IQ-807	26	13	21	1.84
IQ-814	11	13	21	5.16
IQ-819	16	13	21	3.97
IQ-1038	13	13	21	3.92
IQ-1129	4	13	21	1.69
Growth medium control⁑	2	13	21	1.02

* Each growth medium consisted of 9 grams of Nestlé Honey Cheerios® in 150 mL Erlenmeyer flasks inoculated with the morphotype grown in 15 mL potato-dextrose broth.
⁑ Extract of potato-dextrose broth and Nestlé Honey Cheerios®.

S13. Bioactivity of prioritized morphotypes.

In vitro inhibition of *A. baumannii* A564 growth by medium-scaled culture extract

Sample	Percentage inhibition	SD
IQ-807	77.36	19.66
IQ-814	3.27	8.93
IQ-819	23.89	9.52
IQ-1038	0	-
IQ-1129	76.65	1.07
Gentamicin in water*	21.94	1.09
Growth medium control⁑	4.58	3.54
Negative control	0.00	2.19
Sterility control	99.94	0.10

Experiments carried out in triplicate, the average, and its standard deviation (SD) are presented. The extracts were dissolved in DMSO unless otherwise indicated and evaluated at a concentration of 250 µg/mL.
* Positive control evaluated at 64 µg/ mL.
⁑ Extract of potato-dextrose broth and Nestlé Honey Cheerios®.

In vitro inhibition of *A. baumannii* A564 growth by primary fractions

Sample	Primary fraction	Percentage inhibition	SD
IQ-807	Hexanic	0.0	-
IQ-807	Aqueous	0.0	-
IQ-807	Medium polarity	76.12	2.91
IQ-814	Hexanic	0.0	-
IQ-814	Aqueous	0.0	-
IQ-814	Medium polarity	37.00	3.86
IQ-819	Hexanic	5.44	15.04
IQ-819	Aqueous	0.0	-
IQ-819	Medium polarity	58.77	3.63
IQ-1038	Hexanic	0.0	-
IQ-1038	Aqueous	0.0	-
IQ-1038	Medium polarity	0.0	-
IQ-1129	Hexanic	115.11	0.40
IQ-1129	Aqueous	0.0	-
IQ-1129	Medium polarity	100.45	6.82
Gentamicin in water*		27.69	10.46
Growth medium control⁑	Hexanic	16.07	8.18
Growth medium control⁑	Aqueous	0.0	-
Growth medium control⁑	Medium polarity	12.68	9.5
Negative control		0.00	4.08
Sterility control		100.00	0.19

Experiments carried out in triplicate, the average, and its standard deviation (SD) are presented. The extracts were dissolved in DMSO unless otherwise indicated and evaluated at a concentration of 250 µg/mL.

* Positive control evaluated at 64 µg/ mL.

⁑ Extract of potato-dextrose broth and Nestlé Honey Cheerios®.

S14.Purification of compounds

IQ-1129

The medium scale extract (1.69 g) was fractionated by liquid-liquid partition separation, yielding the 8:2 water-MeOH (624 mg), 1:1 MeCN-MeOH (425 mg) and hexane (455 mg) phases. Next, the MeCN-MeOH phase (425 mg) was adsorbed on a mixture of celite (1.69 g) and silica gel (150 mg) to fractionate by reverse-phase CR using a gradient elution system of MeCN-acidic water 5:95→5:95 for 8.5 min, 5:95→100:0 for 10 min, and 100:0→100:0 for 16.5 min, followed by washing with MeCN-MeOH 5:95 for 20 min at a flow rate of 20 mL/min. Eight fractions were obtained. Secondary fraction VII (48.1 mg) was then fractionated by preparative liquid chromatography in two elusions; the sample was injected dissolved in 1:1 dioxane-methanol at 120.25 mg/mL concentration. The elution system consisted of MeOH-acidic water 70:30→85:15 for 23 min at a flow rate of 14 mL/min, followed by washing with MeOH. The eluates were pooled into thirteen fractions. The tertiary fraction VIII (13 mg) was identified as PF1140 (**17**). The tertiary fraction XII (16.8 mg) was fractionated by semi-preparative liquid chromatography. It was injected at a concentration of 56 mg/mL and was eluted with a system of MeCN-acidic water 75:25→85:15 for 30 min at a flow rate of 4.60 mL/min. Seven fractions were obtained. The quaternary fraction II (1.3 mg) was identified as deoxy-PF1140 (**16**).

IQ-1038

The medium scale extract (3.92 g) was fractionated by liquid-liquid partition separation, yielding the 8:2 water-MeOH (1.08 g), 1:1 MeCN-MeOH (1.52 g) and hexane (1.12 g) phases. Next, the MeCN-MeOH phase (1.47 g) was adsorbed on a mixture of celite (2.46 g) and silica gel (700 mg) to fractionate by reverse-phase CR using a gradient elution system of MeCN-acidic water 5:95→5:95 for 7 min, 5:95→100:0 for 12 min, and 100:0→100:0 for 17.8 min, followed by washing with methanol for 20 min at a flow rate of 20 mL/min. Nine fractions were obtained. Secondary fraction III (185.30 mg) was then fractionated by preparative liquid chromatography at the rate of four repeats; the sample was injected dissolved in 1:1 dioxane-methanol at 280.75 mg/mL concentration. The elution system consisted of MeOH-acidic water 30:70→57:43 for 20 min at a flow rate of 15 mL/min, followed by washing with MeOH. The eluates were pooled into six fractions. Within tertiary fraction III, crystals (112.80 mg) formed, were decanted, and corresponded to penicillic acid (**18**).

IQ-1017

In the small-scale 50:25:25 AcOEt-MeOH-DCM extract, tranparent crystals formed and were decanted and washed with hexane. The precipitate (58.9 mg) was identified as brefeldin A (**19**).

IQ-819

The medium scale extract (3.97 g) was fractionated by liquid-liquid partition separation, yielding the 8:2 water-MeOH (613.70 mg), 1:1 MeCN-MeOH (1.58 g) and hexane (1.60 g) phases. Next, the MeCN-MeOH phase (1.45 g) was adsorbed on a mixture of celite (22.01 g) and silica gel (4.61 g) to fractionate by reverse-phase CR using a gradient elution system of MeCN-acidic water 5:95→5:95 for 10 min, 5:95→61:39 for 8.4 min, 61:39→80:20 for 6.6 min, 80:20→88:12 for 1.3 min, 80:20→91:9 for 0.2 min, 91:9→91:9 for 5.7 min, and 91:9→100:0 for 0.8, followed by washing with MeCN-MeOH 5:95→5:95 for 0.5 min, 5:95→0:100 for 1.1 min at a flow rate of 10 mL/min. Ten fractions were obtained. Secondary fraction III (193.10 mg) was then fractionated by preparative liquid chromatography in three elusions; the sample was

injected dissolved in 1:1 dioxane-methanol at 203.26 mg/mL concentration. The elution system consisted of MeOH-acidic water 45:55→90:10 for 25 min at a flow rate of 14 mL/min, followed by washing with MeOH. The eluates were pooled into 16 fractions (A1-A16). The tertiary fraction A3 (18.70 mg) was fractionated by semi-preparative liquid chromatography. It was injected at a concentration of 53.42 mg/mL and was eluted with a system of MeCN-acidic water 45:55→62:38 for 15 min at a flow rate of 4.60 mL/min. Five fractions were obtained (B1-B5). The quaternary fraction B3 (2.70 mg) was identified as trichodermamide A (**23**). The tertiary fraction A11 (94.50 mg) was identified as trichodermic acid A (**21**). The tertiary fraction A12 (10.70 mg) was fractionated by semi-preparative liquid chromatography. It was injected at a concentration of 42.80 mg/mL and was eluted with a system of MeCN-acidic water 60:40→87:13 for 20 min at a flow rate of 4.60 mL/min. Ten fractions were obtained (C1-C10). The quaternary fraction C6 (1.50 mg) was identified as trichodermic acid C (**22**). In secondary fraction V (129 mg), tranparent crystals formed and were decanted, dissolved in methanol, and recrystallized. The precipitate (40.10 mg) was identified as trichodermic acid (**20**).

IQ-814

The medium scale extract (5.16 g) was fractionated by liquid-liquid partition separation, yielding the 8:2 water-MeOH (3.14 g), 1:1 MeCN-MeOH (472.50 mg) and hexane (699 mg) phases. Next, the MeCN-MeOH phase (450.80 mg) was adsorbed on a mixture of celite (2.02 g) and silica gel (6.70 g) to fractionate by reverse-phase CR using a gradient elution system of MeCN-acidic water 5:95→5:95 for 12 min, 5:95→54:46 for 10.1 min, 54:46→100:0 for 3.6 min, 100:0→100:0 for 28 min and MeCN-MeOH 100:0→100:0 for 2 min, 100:0→0:100 for 1 min, followed by washing with methanol for 42 min at a flow rate of 10 mL/min. Eight fractions were obtained. Secondary fraction III (86.30 mg) was then fractionated by preparative liquid chromatography in two elusions; the sample was injected dissolved in 1:1 dioxane-methanol at 123.28 mg/mL concentration. The elution system consisted of MeOH-acidic water 40:60→73:27 for 20 min at a flow rate of 15 mL/min, followed by washing with MeOH. The eluates were pooled into twelve fractions. The tertiary fraction VIII (9.20 mg) was fractionated by semi-preparative liquid chromatography. It was injected at a concentration of 61.33 mg/mL and was eluted with a system of MeCN-acidic water 45:55→70:30 for 30 min at a flow rate of 4.60 mL/min. Six fractions were obtained. The quaternary fraction IV (2.2 mg) was identified as (*E*)-tridec-7-ene-3,5,6,10-tetraol (**24**).

IQ-807

The medium scale extract (1.84 g) was fractionated by liquid-liquid partition separation, yielding the 8:2 water-MeOH (350.60 mg), 1:1 MeCN-MeOH (459.10 mg) and hexane (997.10 mg) phases. Next, MeCN-MeOH phase (433 mg) was adsorbed on a mixture of celite (2.01 g) and silica gel (3.18 g) to fractionate by reverse-phase CR using a gradient elution system of MeCN-acidic water 5:95→5:95 for 12 min, 5:95→32:68 for 4.8 min, 32:68→65:35 for 11.4 min, 65:35→65:35 for 11 min, 65:35→80:20 for 2.6 min, 80:20→80:20 for 11 min, 80:20→100:0 for 1.7 min, and 100:0→100:0 for 12 min, followed by washing with methanol for 20 min at a flow rate of 10 mL/min. Seven fractions were obtained. Secondary fraction I (50.2 mg) was then fractionated by preparative liquid chromatography; the sample was injected dissolved in 1:1 dioxane-methanol at 111.55 mg/mL concentration. The elution system consisted of MeOH-acidic water 20:80→76:24 for 26 min at a flow rate of 14 mL/min, followed by washing with MeOH. The eluates were pooled into twelve fractions. The tertiary fraction V (14.5 mg) was fractionated by semi-preparative liquid chromatography. It was injected at a concentration

of 45.31 mg/mL and was eluted with a system of MeCN-acidic water 45:55→68:32 for 20 min at a flow rate of 4.6 mL/min. Five fractions were obtained. The quaternary fraction II (11.7 mg) was identified as *iso*-Cladospolide B (**25**).

Growth medium control

The medium scale extract (1.02 g) was fractionated by liquid-liquid partition separation, yielding the 8:2 water-MeOH (762.50 mg), 1:1 MeCN-MeOH (26.10 mg) and hexane (203.90 mg) phases.

S15.Spectroscopic and spectrometric data of compounds isolated from IQ-1129

Deoxy-PF1140 (**16**)

Light yellow powder; HR-DART, *m/z* 262.1817 [M+H]$^+$ (calculated for $C_{16}H_{24}NO_2$, 262.1817, Δ 0.00 ppm); $[\alpha]^{25}_{D}$ -130.53 (*c* 0.0019, MeOH); ^{1}H NMR (700 MHz, $CDCl_3$) and ^{13}C NMR (175 MHz, $CDCl_3$) see below. Similar information to De Silva *et al.* (2009).[1]

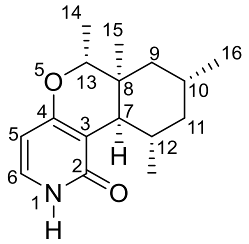

Spectroscopy data for **16** in $CDCl_3$

Position	Type	δ_C ppm	δ_H ppm	Multiplicity (*J* in Hz)
14	CH_3	14.8	1.26	d, *J* = 6.56.
16	CH_3	23.0	0.87	d, *J* = 6.45.
15	CH_3	21.7	0.71	s
17	CH_3	20.6	0.94	d, *J* = 6.56.
10	CH	27.1	1.56	m
8	C	33.9		
12	CH	38.0	1.62	m
11	CH_2	44.4	1.69	m
			0.90	m
9	CH_2	44.6	1.73	m
			1.04	t, *J* = 13.53.
7	CH	45.4	2.27	d, *J* = 10.86.
13	CH	74.5	4.64	q, *J* = 6.51.
5	CH	98.2	5.87	d, *J* = 7.64.
3	C	109.5		
6	CH	128.1	7.49	d, *J* = 7.64.
4	C	158.6		
2	C	160.0		
1	-NH			

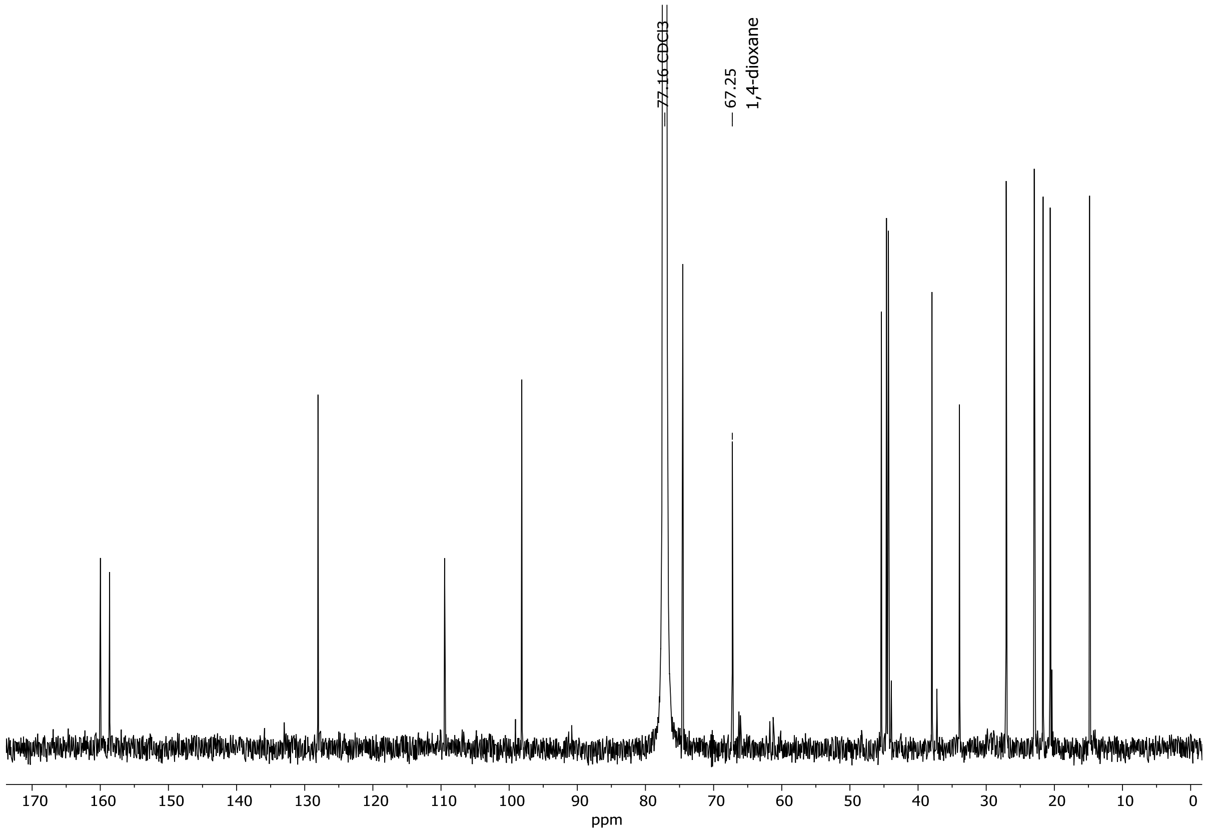

^{13}C NMR spectrum (175 MHz, $CDCl_3$) of **16**.

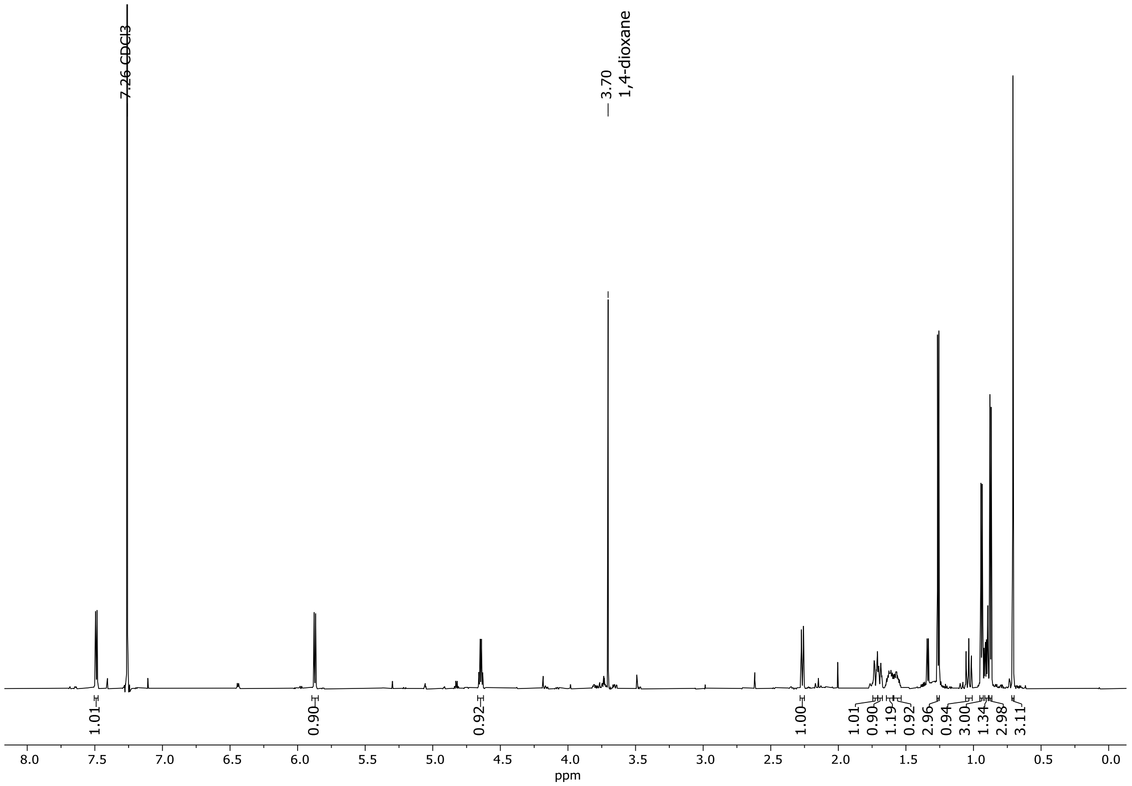

^{1}H NMR spectrum (700 MHz, $CDCl_3$) of **16**.

PF1140 (**17**)

Light yellow powder; HR-DART, *m/z* 278.1749 $[M+H]^+$ (calculated for $C_{16}H_{24}NO_3$, 278.1756, Δ 2.52 ppm); $[\alpha]^{25}_D$ -146.00 (*c* 0.0015, MeOH); ^{1}H NMR (400 MHz, $CDCl_3$) and ^{13}C NMR (100 MHz, $CDCl_3$) see below. Similar information to De Silva *et al.* (2009).[1]

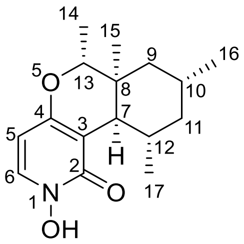

Spectroscopy data for **17** in $CDCl_3$

Position	Type	δ_C ppm	δ_H ppm	Multiplicity (J in Hz)
14	CH_3	14.8	1.25	d, J = 6.55.
16	CH_3	20.5	0.86	d, J = 6.38.
15	CH_3	21.6	0.70	s
17	CH_3	23.0	0.88	d, J = 6.55.
10	CH	27.0	1.54	m
8	C	33.9		
12	CH	37.9	1.60	m
11	CH_2	44.3	1.66	m
			0.89	m
9	CH_2	44.6	1.71	m
			1.02	dd, J = 14.06, 12.72.
7	CH	45.3	2.26	d, J = 10.83.
13	CH	74.6	4.63	q, J = 6.63.
5	CH	98.6	5.87	d, J =7.64.
3	C	109.8		
6	CH	129.5	7.48	d, J =7.64.
4	C	159.2		
2	C	160.2		
1	-OH			

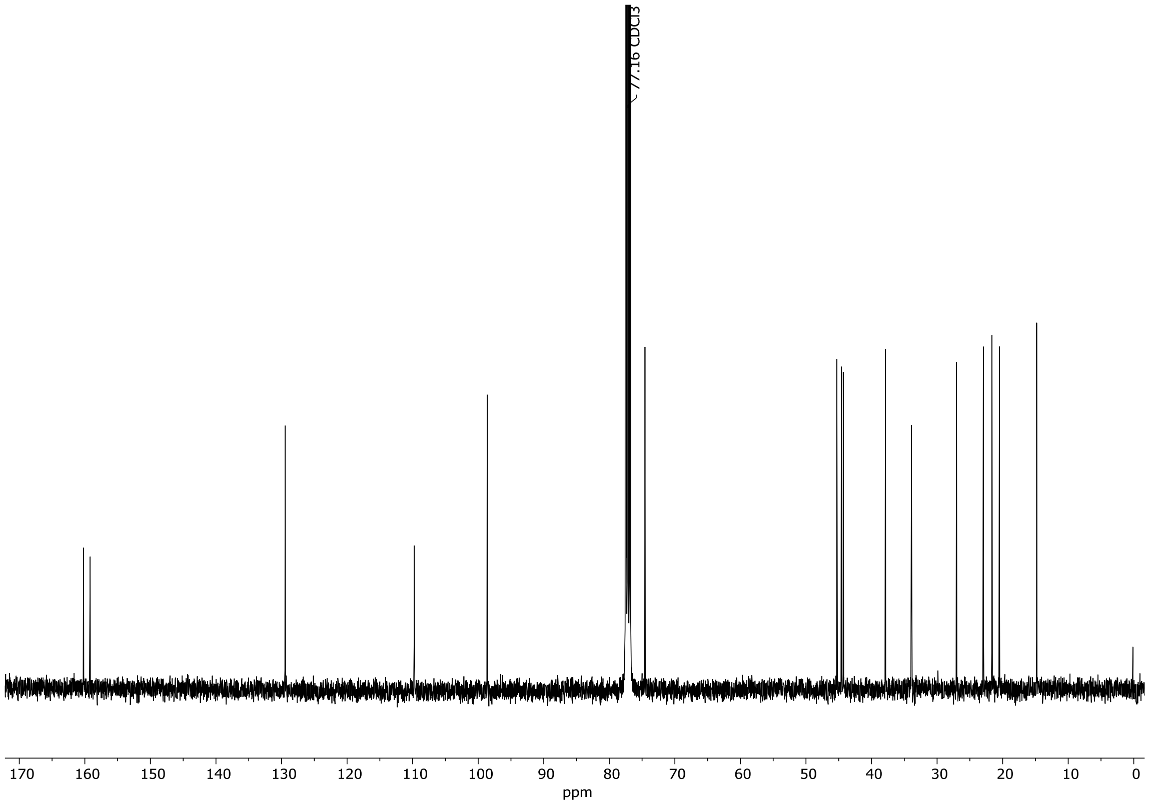

^{13}C NMR spectrum (100 MHz, $CDCl_3$) of **17**.

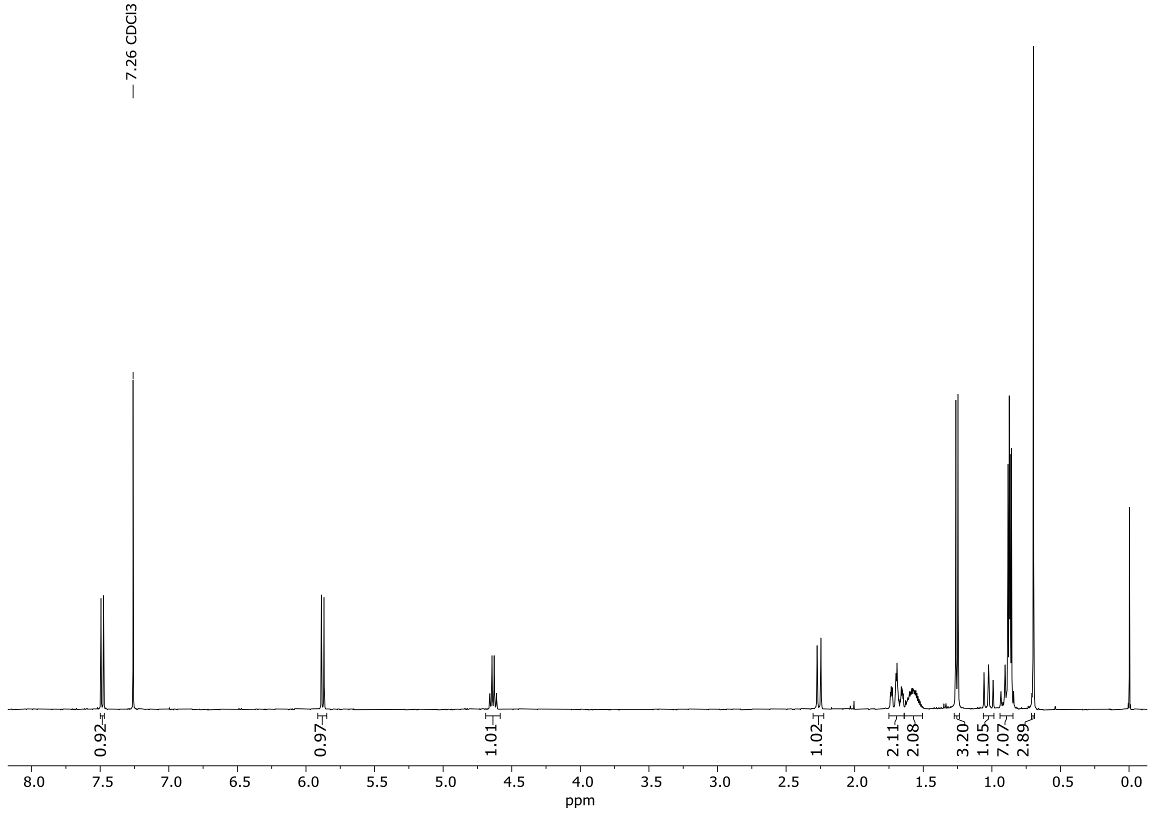

^{1}H NMR spectrum (400 MHz, $CDCl_3$) of **17**.

S16.Spectroscopic and spectrometric data of compounds isolated from IQ-1038

Penicillic acid (**18**)

White powder; HR-DART, *m*/*z* 323.1134 [2M+H-H_2O]$^+$ (calculated for $C_{16}H_{19}O_7$, 323.1130, Δ 1.24 ppm); $[\alpha]^{25}_D$ -0.16 (*c* 0.00245, MeOH); ^{1}H NMR (700 MHz, $CDCl_3$) and ^{13}C NMR (175 MHz, $CDCl_3$) see below. Similar information to Nonaka *et al*. (2015).[2]

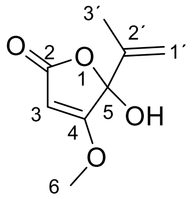

Spectroscopy data for **18** in $CDCl_3$

Position	Type	δ_C ppm	δ_H ppm	Multiplicity (*J* in Hz)
4	C	179.5		
2	C	171.6		
2´	C	139.6		
1´	CH_2	116.5	5.18	s
			5.46	s
5	C	103.2		
3	CH	89.4	5.12	s
6	CH_3	60.1	3.91	s
3´	CH_3	17.5	1.75	s

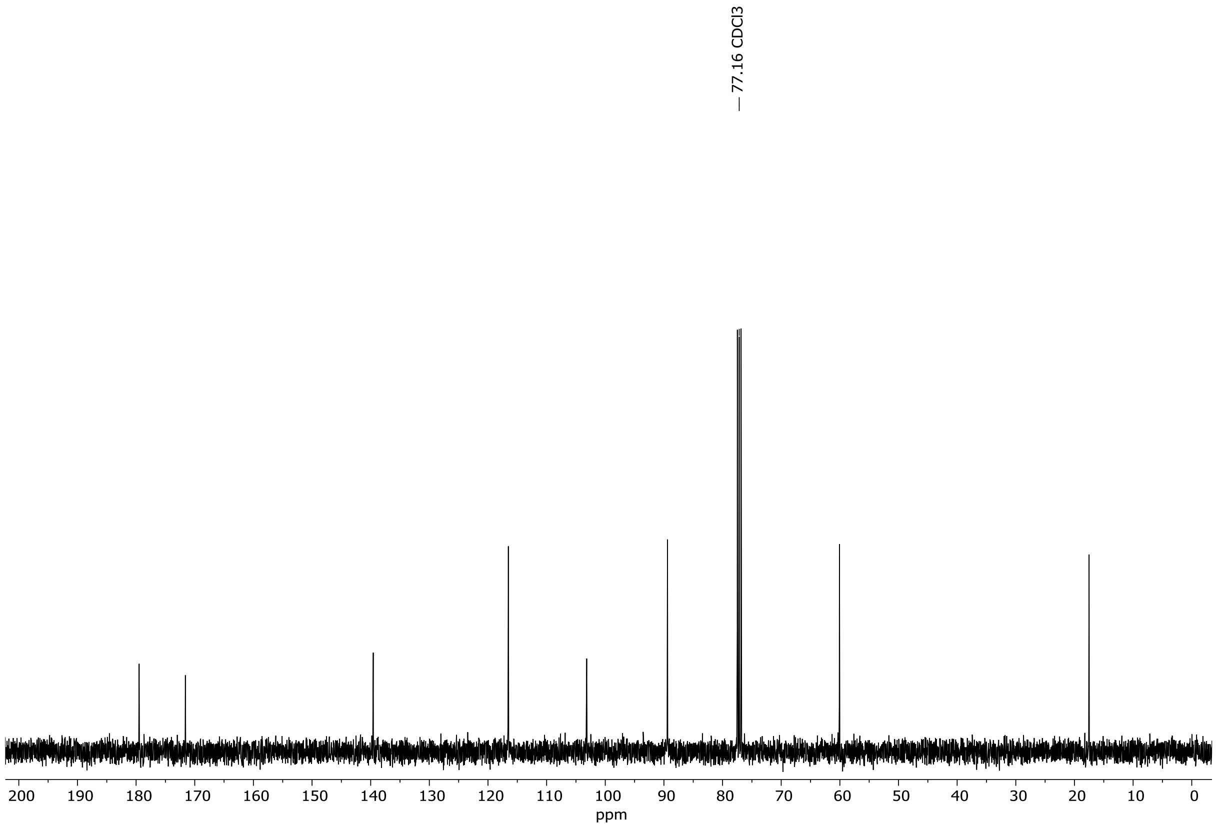

^{13}C NMR spectrum (100 MHz, $CDCl_3$) of **18**.

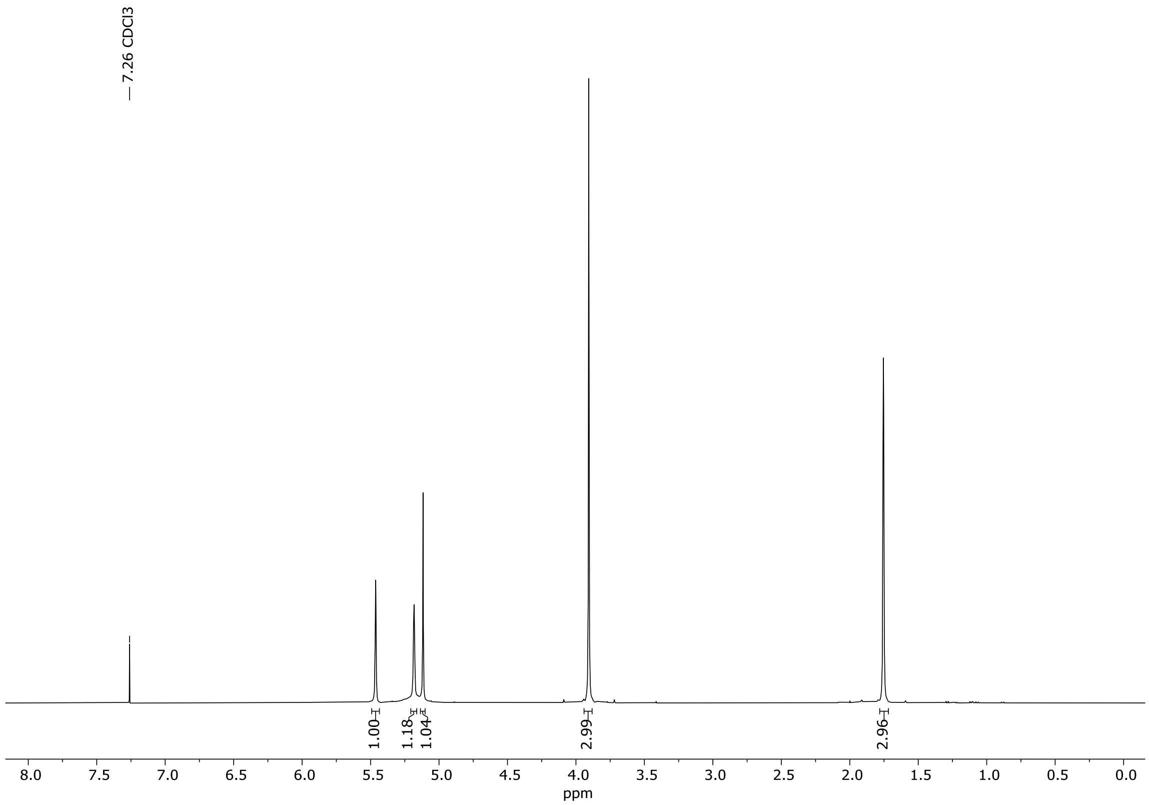

^{1}H NMR spectrum (400 MHz, $CDCl_3$) of **18**.

S17.Spectroscopic and spectrometric data of compounds isolated from IQ-1017

Brefeldin A (**19**)

Transparent crystalline solid; HR-DART, *m/z* 281.1763 [M+H]$^+$ (calculated for $C_{16}H_{25}O_4$, 281.1752, Δ 3.86 ppm); $[\alpha]^{25}_D$ +20.00 (*c* 0.0017, MeOH); ^{1}H NMR (700 MHz, DMSO-*d*6) and ^{13}C NMR (175 MHz, DMSO-*d*6) see below. Similar information to Glaser *et al*. (2000) and Vurro *et al*. (1998).[3,4]

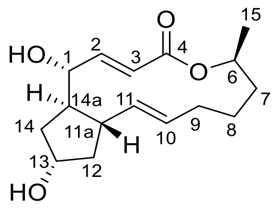

Spectroscopy data for **19** in DMSO-*d*6

Position	Type	δ_C ppm	δ_H ppm	Multiplicity (*J* in Hz)
15	CH_3	20.7	1.18	d, *J* = 6.21.
8	CH_2	26.4	0.75	m
			1.79	dd, *J* = 6.57, 3.31.
9	CH_2	31.4	1.92	tdd, *J* = 10.33, 4.81, 2.37.
			1.76	ddd, *J* = 4.3, 2.69, 1.29.
7	CH_2	33.4	1.48	m
			1.72	m
14	CH_2	40.9	1.65	m
			1.82	dddd, *J* = 13.14, 8.6, 4.7, 1.35.
12	CH_2	43.0	1.97	ddd, *J* = 11.62, 8.71, 5.06.
			1.29	dddd, *J* = 13.12, 7.96, 5.38, 1.29.
11a	CH	43.3	2.30	m
14a	CH	51.7	1.70	dd, *J* = 9.68, 8.02.
6	CH	70.5	4.04	p, *J* = 5.38.
13	CH	70.8	4.70	dqd, *J* = 12.69, 6.45, 1.72.
1	CH	74.3	3.92	dt, *J* = 9.9, 2.58.
3	CH	116.3	5.71	dd, *J* = 15.60, 2.01.
10	CH	129.2	5.66	ddd, *J* = 15.06, 10.33, 4.62.
11	CH	137.1	5.20	dd, *J* = 15.27, 9.68.
2	CH	154.4	7.34	dd, *J* = 15.49, 3.01.
4	-COO-	165.7	1.18	d, *J* = 6.21.

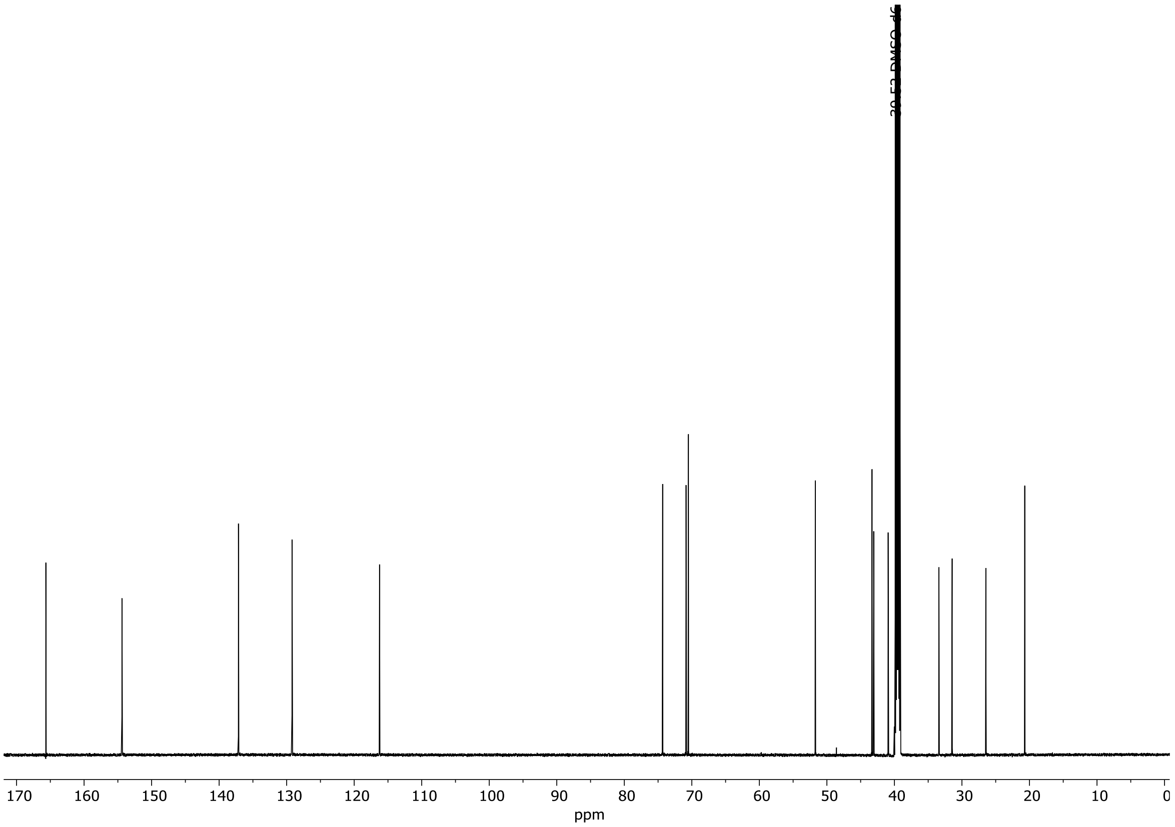

^{13}C NMR spectrum (175 MHz, DMSO-*d6*) of **19**.

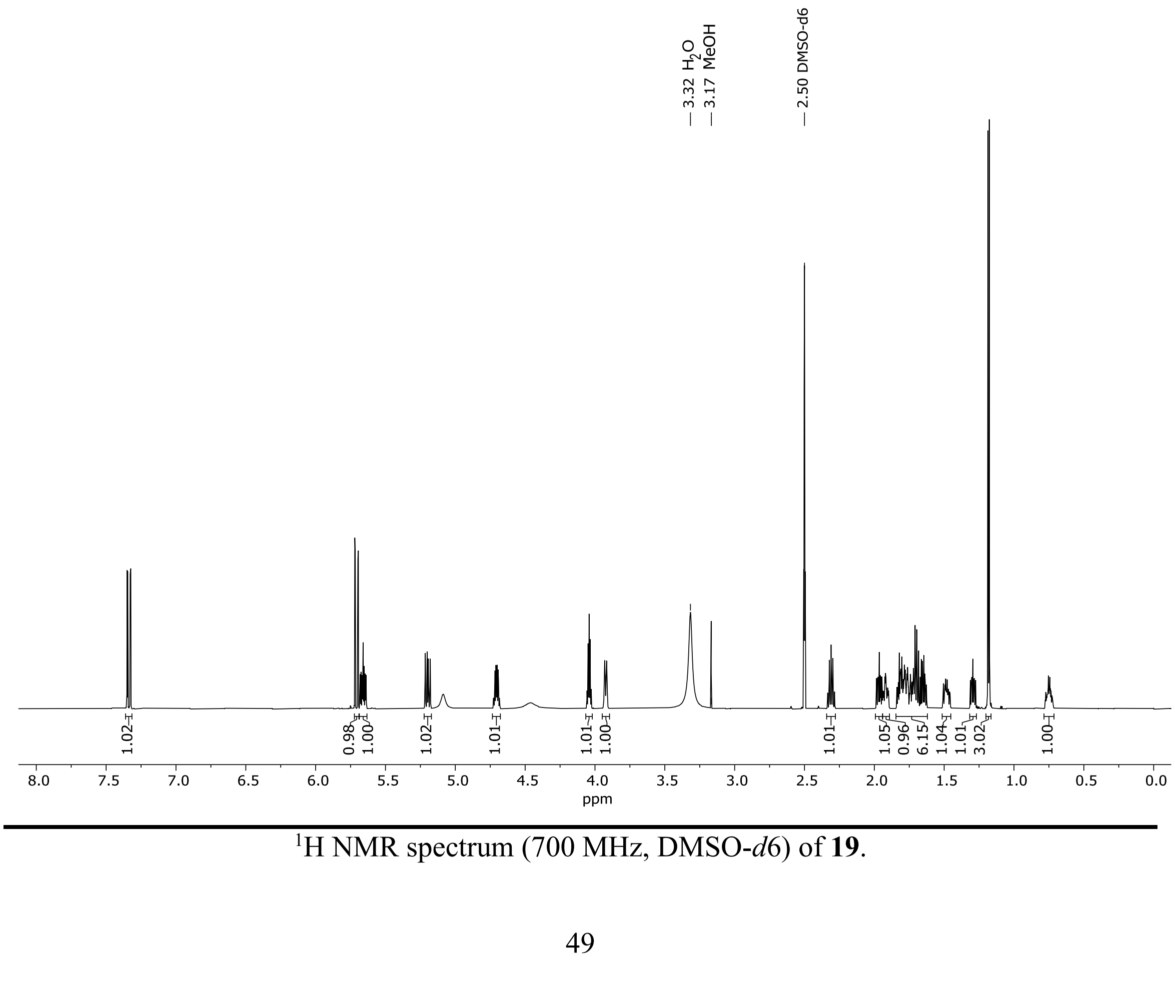

^{1}H NMR spectrum (700 MHz, DMSO-*d6*) of **19**.

S18.Spectroscopic and spectrometric data of compounds isolated from IQ-819

Trichodermic acid (**20**)

Transparent needles; HR-ESI+-QTOF, *m/z* 305.2130 $[M+H]^+$ (calculated for $C_{19}H_{29}O_3$, 305.2116, Δ 4.6 ppm); $[\alpha]^{25}_D$ +50.00 (*c* 0.0014, MeOH); ^1H NMR (700 MHz, $CDCl_3$) and ^{13}C NMR (175 MHz, $CDCl_3$) see below. Comparable data with Shiina *et al*. (2013).[5]

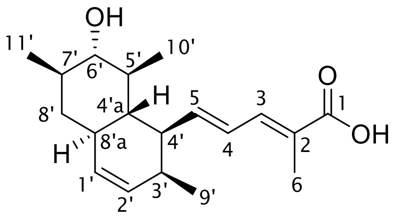

Spectroscopy data for **20** in $CDCl_3$

Position	Type	δ_C ppm	δ_H ppm	Multiplicity (*J* in Hz)
1	C	172.7		
2	C	124.2		
3	CH	140.9	7.29	dd, *J* = 11.02, 1.56.
4	CH	124.6	6.28	dd, *J* = 15.0, 11.13.
5	CH	149.5	6.20	dd, *J* = 15.0, 10.46.
6	CH3	12.5	1.94	d, *J* = 1.51.
1'	CH	131.6	5.46	dt, *J* = 9.46, 2.04.
2'	CH	132.9	5.59	ddd, *J* = 9.46, 4.36, 2.74.
3'	CH	36.7	2.22	m
4'	CH	50.0	2.50	ddd, *J* = 10.43, 8.98, 3.58.
4'a	CH	46.0	1.09	ovl
5'	CH	44.0	1.32	m
6'	CH	82.4	2.74	t, *J* = 9.63.
7'	CH	39.7	1.49	m
8'	CH2	39.4	1.76	dt, *J* = 13.25, 3.52.
			0.97	ovl
8'a	CH	42.0	1.88	ddt, *J* = 12.62, 10.09, 2.53
9'	CH3	16.7	0.96	d, *J* =7.10.
10'	CH3	18.2	1.04	d, *J* = 6.45.
11'	CH3	19.2	1.08	d, *J* = 6.35.

Ovl: overlapped with other signals.

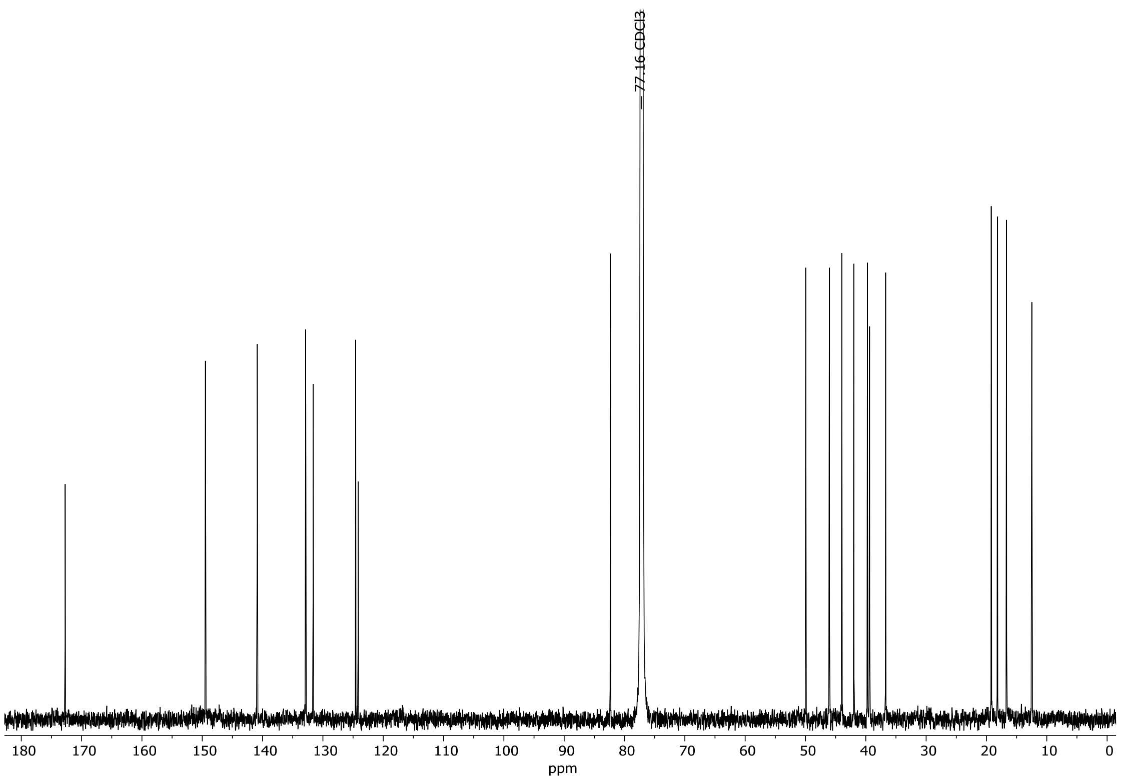

^{13}C NMR spectrum (175 MHz, $CDCl_3$) of **20**.

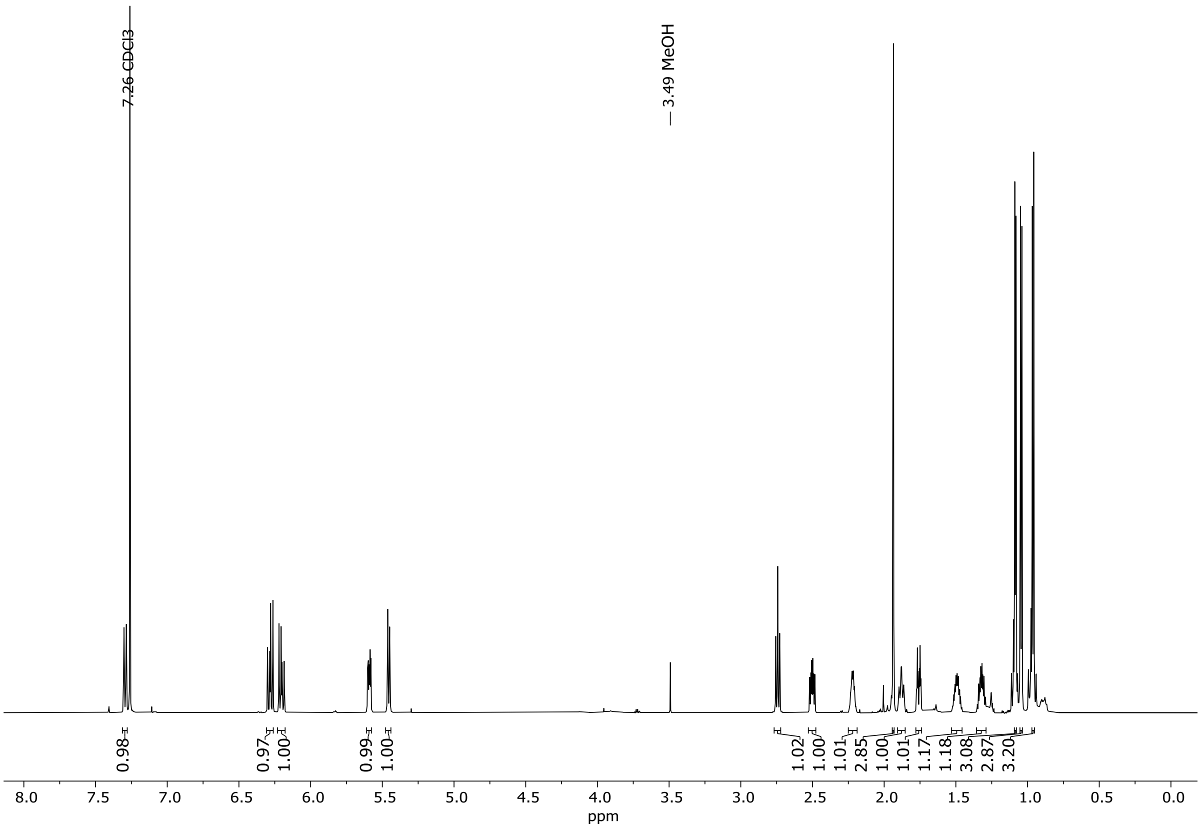

^{1}H NMR spectrum (700 MHz, $CDCl_3$) of **20**.

Trichodermic acid A (**21**)

Solid white; HR-DART, *m/z* 321.2067 [M+H]$^+$ (calculated for $C_{19}H_{29}O_4$, 321.2065, Δ 0.6 ppm); $[\alpha]^{25}_D$ +50.94 (*c* 0.00695, MeOH); ^{1}H NMR (700 MHz, $CDCl_3$) and ^{13}C NMR (175 MHz, $CDCl_3$) see below. Comparable data with Li *et al*. (2012).[6]

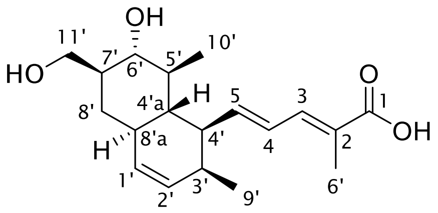

Spectroscopy data for **21** in $CDCl_3$

Position	Type	δ_C ppm	δ_H ppm	Multiplicity (*J* in Hz)
1	C	172.8		
2	C	124.4		
3	CH	140.7	7.29	d, *J* = 12.05.
4	CH	124.7	6.29	dd, *J* = 14.9, 11.24.
5	CH	149.1	6.19	dd, *J* = 14.05, 10.54.
6	CH3	12.5	1.94	d, *J* = 1.4.
1'	CH	131.1	5.45	dt, *J* = 9.47, 2.04.
2'	CH	133.2	5.61	ddd, *J* = 9.57, 4.36, 2.74.
3'	CH	36.7	2.22	m
4'	CH	49.7	2.51	ddd, *J* = 10.45, 9.08, 5.47.
4'a	CH	45.3	1.05	ovl
5'	CH	43.9	1.41	ddq, *J* = 10.57, 9.46, 6.28.
6'	CH	81.9	3.15	t, *J* = 9.63.
7'	CH	45.5	1.76	m
8'	CH2	33.1	1.67	dt, *J* =13.12, 3.5.
			0.91	q, *J* = 12.85.
8'a	CH	41.6	1.92	ovl
9'	CH3	16.6	0.96	d, *J* = 7.1.
10'	CH3	17.5	1.08	d, *J* =6.35.
11'	CH2	68.8	3.66	dd, *J* = 10.70, 8.66.
			3.75	dd, *J* = 10.65, 3.66.

Ovl: overlapped with other signals.

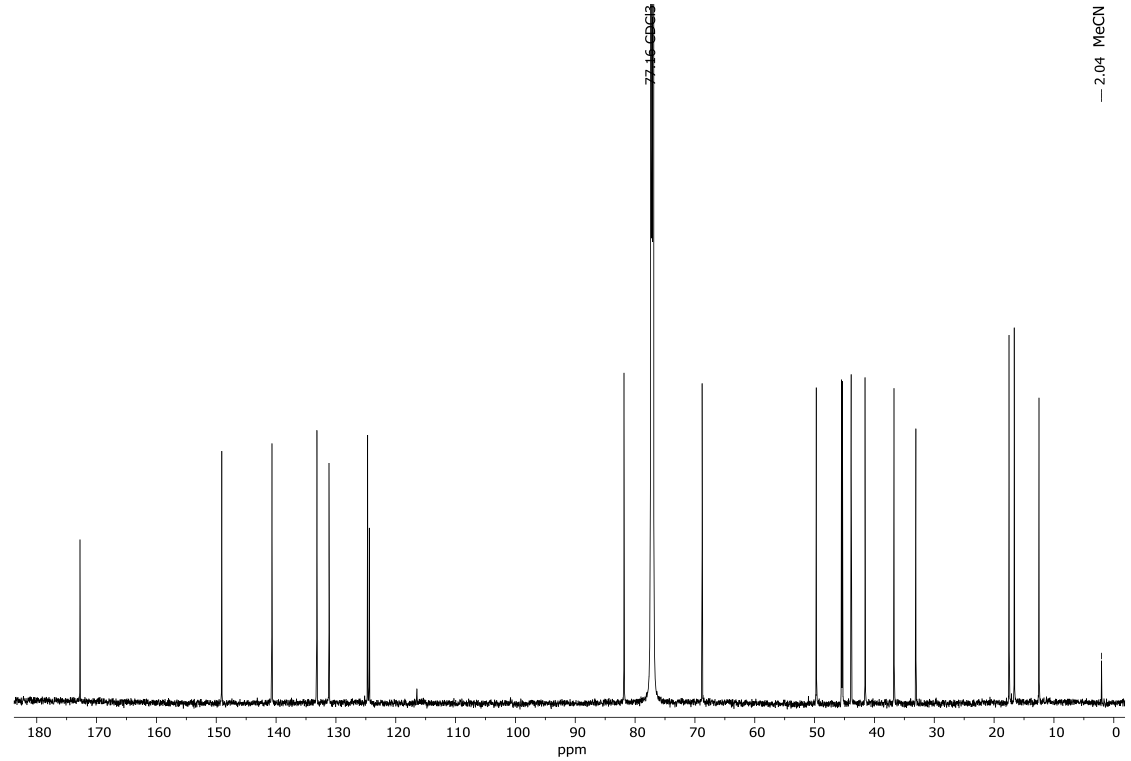

^{13}C NMR spectrum (175 MHz, $CDCl_3$) of **21**.

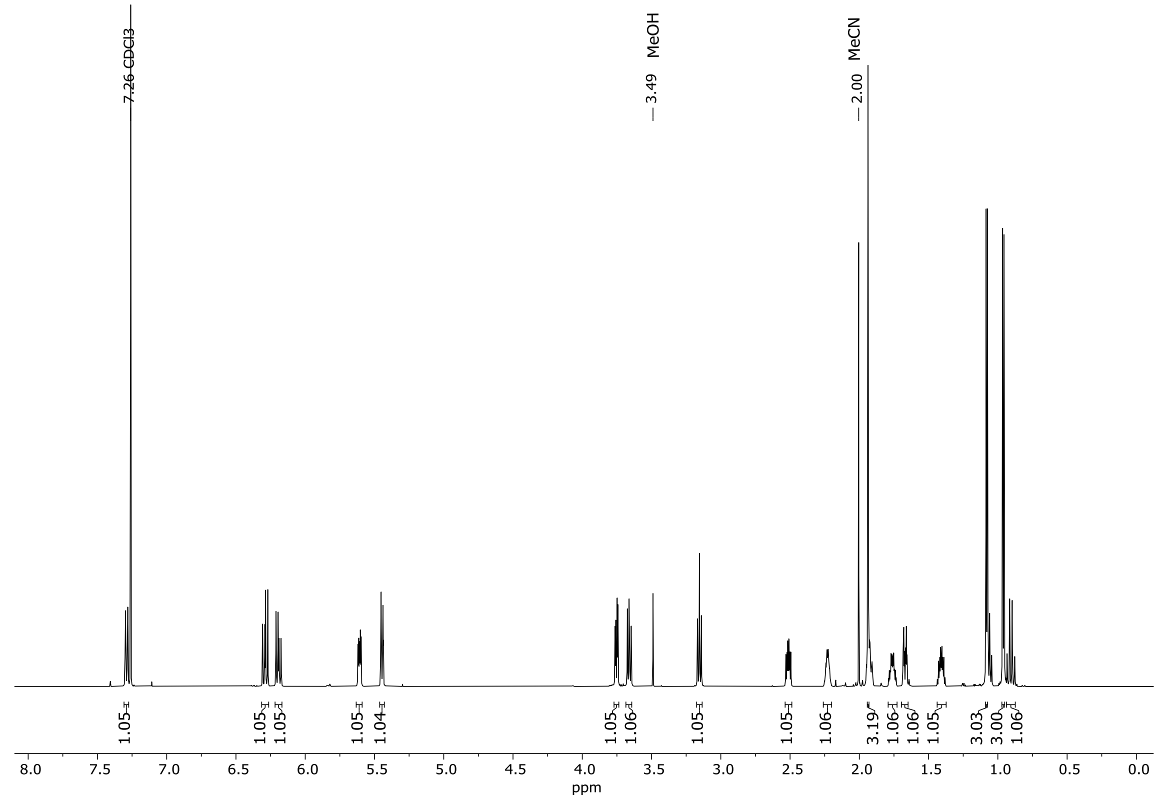

^{1}H NMR spectrum (700 MHz, $CDCl_3$) of **21**.

Trichodermic acid C (**22**)

Transparent needles; HR-DART, *m/z* 303.1949 $[M+H-H_2O]^+$ (calculated for $C_{19}H_{27}O_3$, 303.1960, Δ 3.6 ppm); 1H NMR (700 MHz, CD_3OD) and 13C NMR (175 MHz, CD_3OD) see below. Comparable data with Sofian *et al*. (2023).[7]

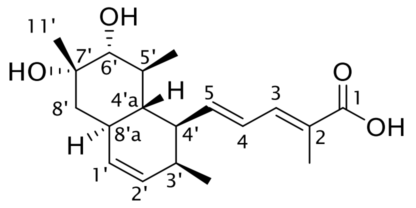

Spectroscopy data for **22** in CD_3OD

Position	Type	δ_C ppm	δ_H ppm	Multiplicity (*J* in Hz)
1	C	172.6		
2	C	126.8		
3	CH	139.8	7.18	d, *J* = 11.29.
4	CH	125.7	6.35	dd, *J* = 14.96, 11.27.
5	CH	149.3	6.18	dd, *J* = 14.95, 10.55.
6	CH3	12.8	1.91	d, *J* = 1.27.
1'	CH	132.7	5.43	dt, *J* = 6.54, 2.05.
2'	CH	133.9	5.62	ddd, *J* = 9.56, 4.36, 2.81.
3'	CH	37.63/37.99	2.22	m
4'	CH	51.1	2.54	ddd, *J* = 10.58, 9.07, 5.46.
4'a	CH	47.2	1.05	ovl
5'	CH	40.4	1.57	tq, *J* = 10.33, 6.33.
6'	CH	81.8	2.83	d, *J* = 10.01.
7'	C	72.4		
8'	CH2	45.0	1.26	t, J = 1.26.
			1.78	dd, *J* = 13.73, 3.25.
8'a	CH	37.63/37.98	2.28	ddt, *J* = 12.88, 10.33, 2.56
9'	CH3	17.0	0.98	d, *J* = 7.1.
10'	CH3	18.6	1.08	d, *J* = 6.35.
11'	CH3	28.0	1.24	s

Ovl: overlapped with other signals.

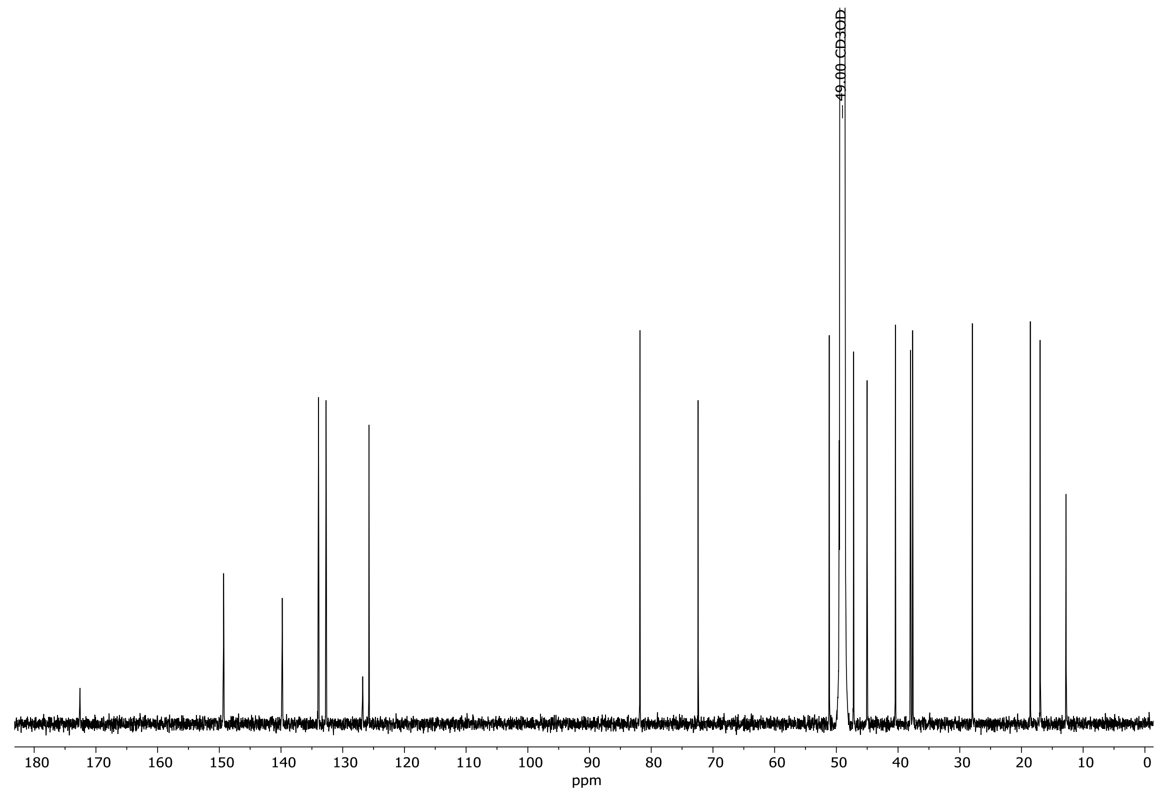

^{13}C NMR spectrum (175 MHz, CD_3OD) of **22**.

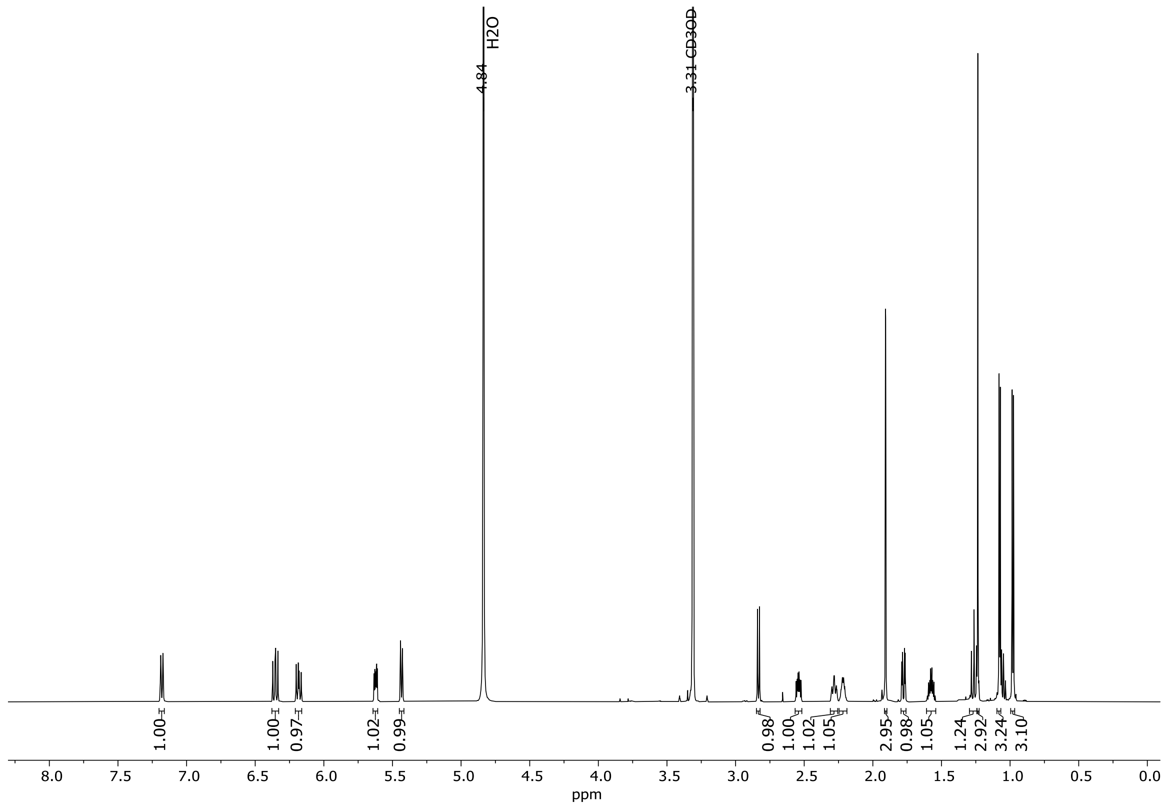

^{1}H NMR spectrum (700 MHz, CD_3OD) of **22**.

Trichodermamide A (**23**)

Transparent crystalline solid; HR-DART, *m/z* 433.1253 $[M+H]^+$ (calculated for $C_{20}H_{21}N_2O_9$, 433.1247, Δ 1.4 ppm); $[\alpha]^{25}_D$ +268.57 (*c* 0.0007, MeOH); ^1H NMR (700 MHz, $CD_3(CO)CD_3$) and ^{13}C NMR (175 MHz, $CD_3(CO)CD_3$) see below. Comparable data with Garo *et al*. (2003).[8]

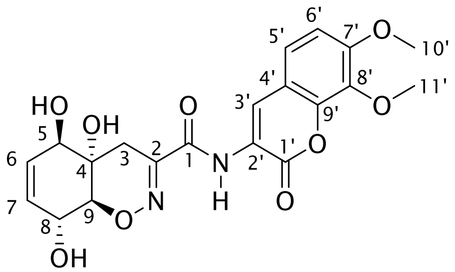

Spectroscopy data for **23** in $CD_3(CO)CD_3$

Position	Type	δ_C ppm	δ_H ppm	Multiplicity (*J* in Hz)
1	C	162.4		
2	C	151.3		
3	CH_2	24.5	2.73	dd, *J* = 19.28, 2.31.
			2.27	d, *J* = 19.30.
4	C	69.1		
5	CH	130.9	5.60	dt, *J* = 10.43, 2.10.
6	CH	129.0	5.55	dt, *J* = 10.43, 2.31.
7	CH	75.0	4.48	dq, *J* = 5.06, 2.47.
8	CH	68.1	4.23	ddt, *J* = 7.85, 5.38, 2.74.
9	CH	85.7	4.15	dd, *J* = 7.8, 2.31.
1'	C	159.1		
2'	C	122.6		
3'	CH	124.0	8.60	s
4'	C	115.3		
5'	CH	123.7	7.42	d, *J* = 8.71.
6'	CH	111.0	7.13	d, *J* = 8.71.
7'	C	155.4		
8'	C	137.2		
9'	C	145.1		
10'	CH_3	57.0	3.96	s
11'	CH_3	61.5	3.92	s
4	-OH		2.76	s
8	-OH		4.62	d, *J* = 5.70.
7	-OH		4.53	d, *J* = 4.90.
	-NH		9.40	s

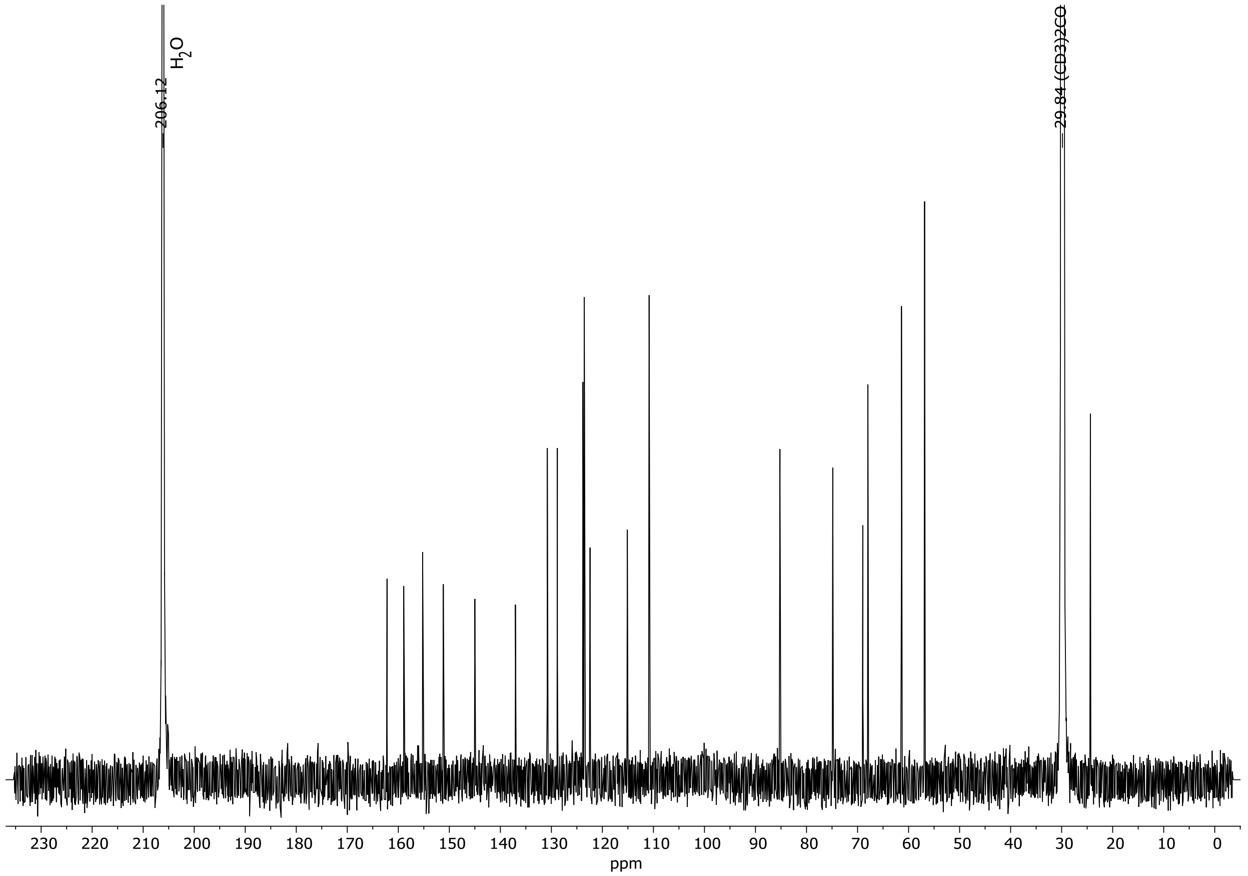

^{13}C NMR spectrum (175 MHz, $CD_3(CO)CD_3$) of **23**.

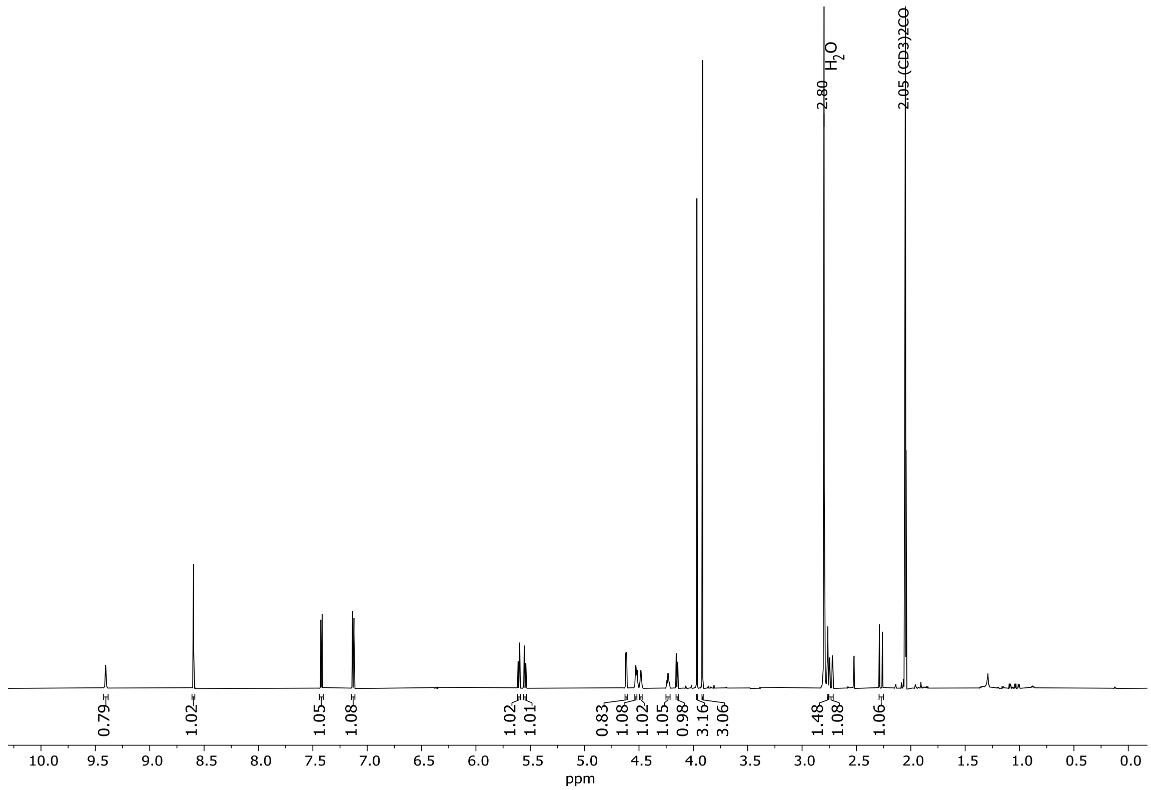

^{1}H NMR spectrum (700 MHz, $CD_3(CO)CD_3$) of **23**.

S19.Spectroscopic and spectrometric data of compounds isolated from IQ-814

(*E*)-tridec-7-ene-3,5,6,10-tetraol (**25**)

Liquid yellow; HR-ESI+-QTOF, *m/z* 229.1812 $[M+H-H_2O]^+$ (calculated for $C_{13}H_{25}O_3$, 229.1803, Δ 3.9 ppm); $[\alpha]^{25}_D$ +11.7 (*c* 0.006, MeOH); ^1H NMR (700 MHz, $CDCl_3$) and ^{13}C NMR (175 MHz, $CDCl_3$) see below.

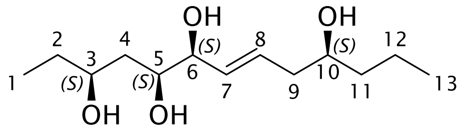

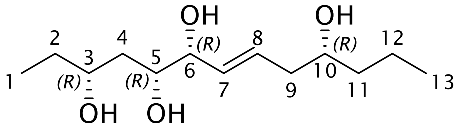

Spectroscopy data for **25** in $CDCl_3$

Position	Type	δ_C ppm	δ_H ppm	Multiplicity (*J* in Hz)
1	CH3	10.3	0.94	t, *J* = 7.42.
2	CH2	29.7	1.56	ddd, *J* = 13.66, 7.48, 6.4.
			1.71	ddd, *J* = 13.87, 7.48, 6.56.
3	CH	79.0	4.00	dq, *J* = 7.85, 6.67.
4	CH2	39.8	1.63	ddd, *J* = 12.69, 7.74, 6.45.
			2.35	dt, *J* = 12.58, 6.78.
5	CH	76.8	4.11	td, *J* = 6.56, 5.27.
6	CH	85.3	4.15	t, *J* = 6.13.
7	CH	131.9	5.55	ddt, *J* = 15.27, 6.96, 1.34.
8	CH	130.1	5.78	dddd, *J* = 15.49, 7.74, 6.82, 1.09.
9	CH2	40.5	2.28	m
			2.16	dt, *J* = 15.38 7.58.
10	CH	70.8	3.67	m
11	CH2	39.1	1.45	ovl
12	CH2	19.0	1.36	q, *J* = 7.53.
			1.45	ovl
13	CH3	14.2	0.93	t, *J* = 7.10.

Ovl: overlapped with other signals.

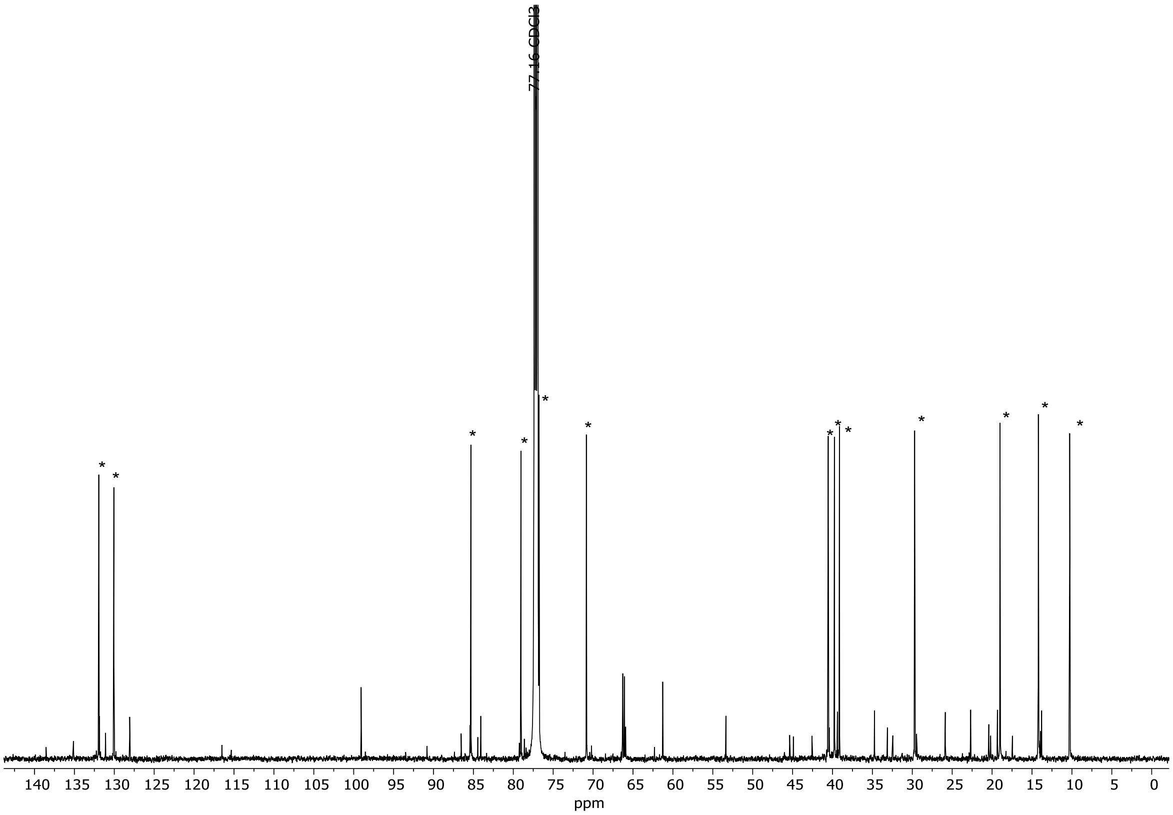

* Compound signals.

^{13}C NMR spectrum (175 MHz, $CDCl_3$) of **25**.

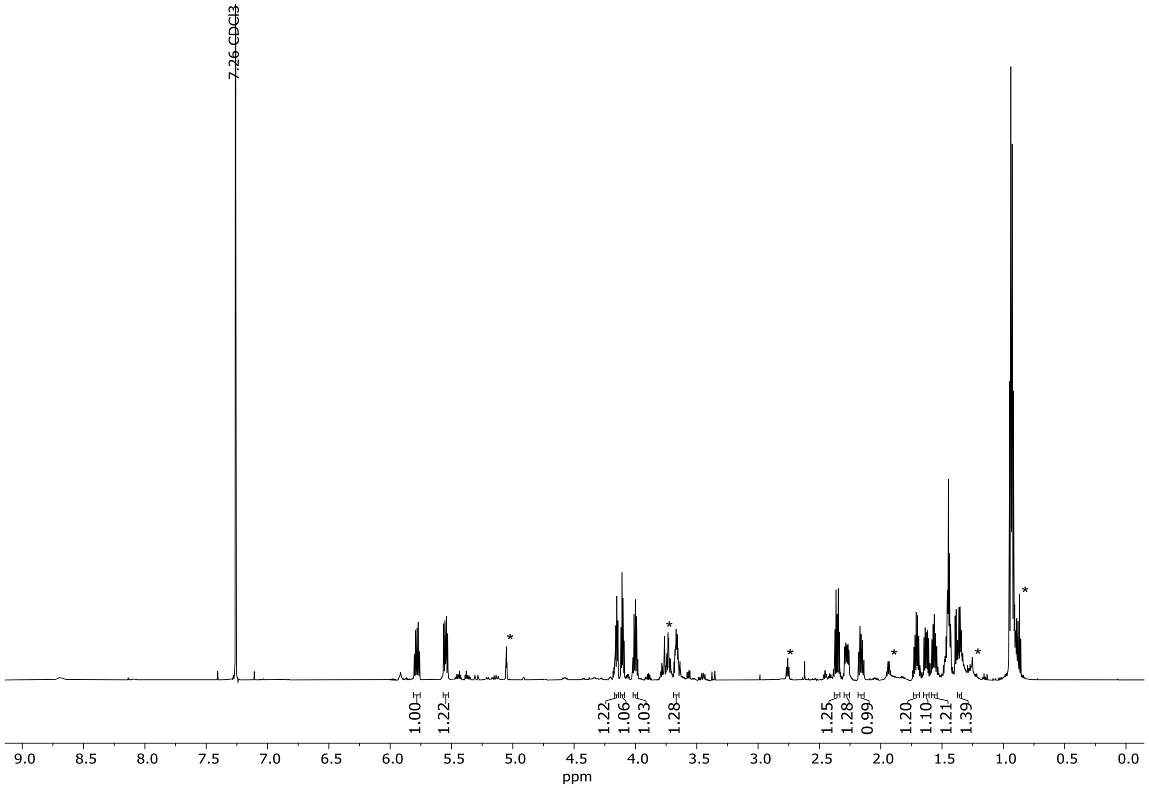

* Impurities.

^{1}H NMR spectrum (700 MHz, $CDCl_3$) of **25**.

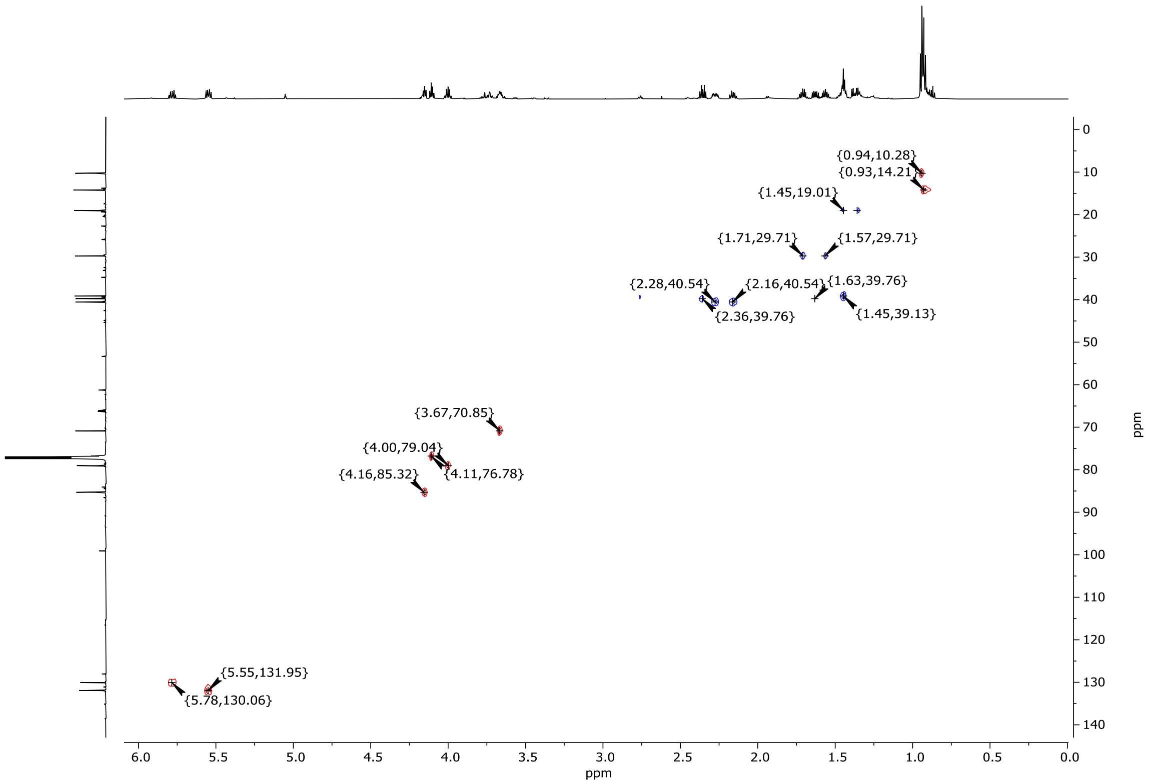

HSQC NMR spectrum (700 MHz, $CDCl_3$) of **25**.

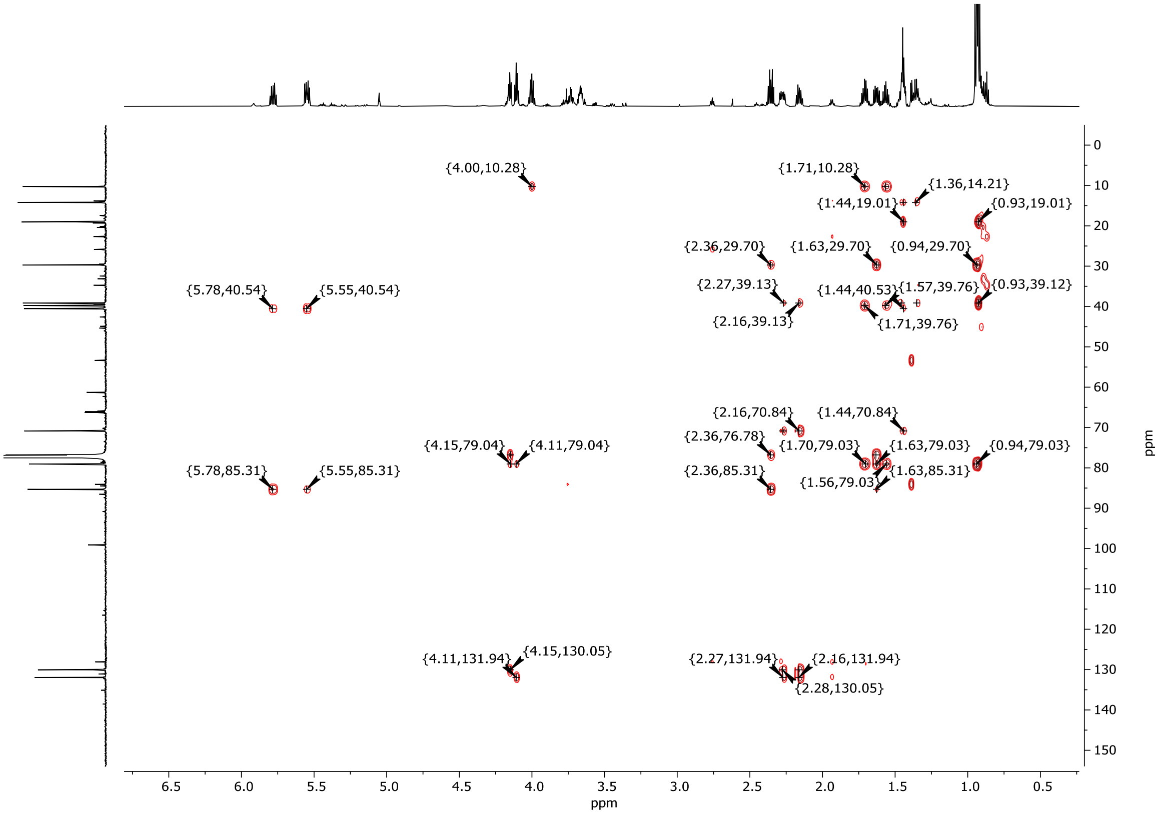

HMBC NMR spectrum (700 MHz, $CDCl_3$) of **25**.

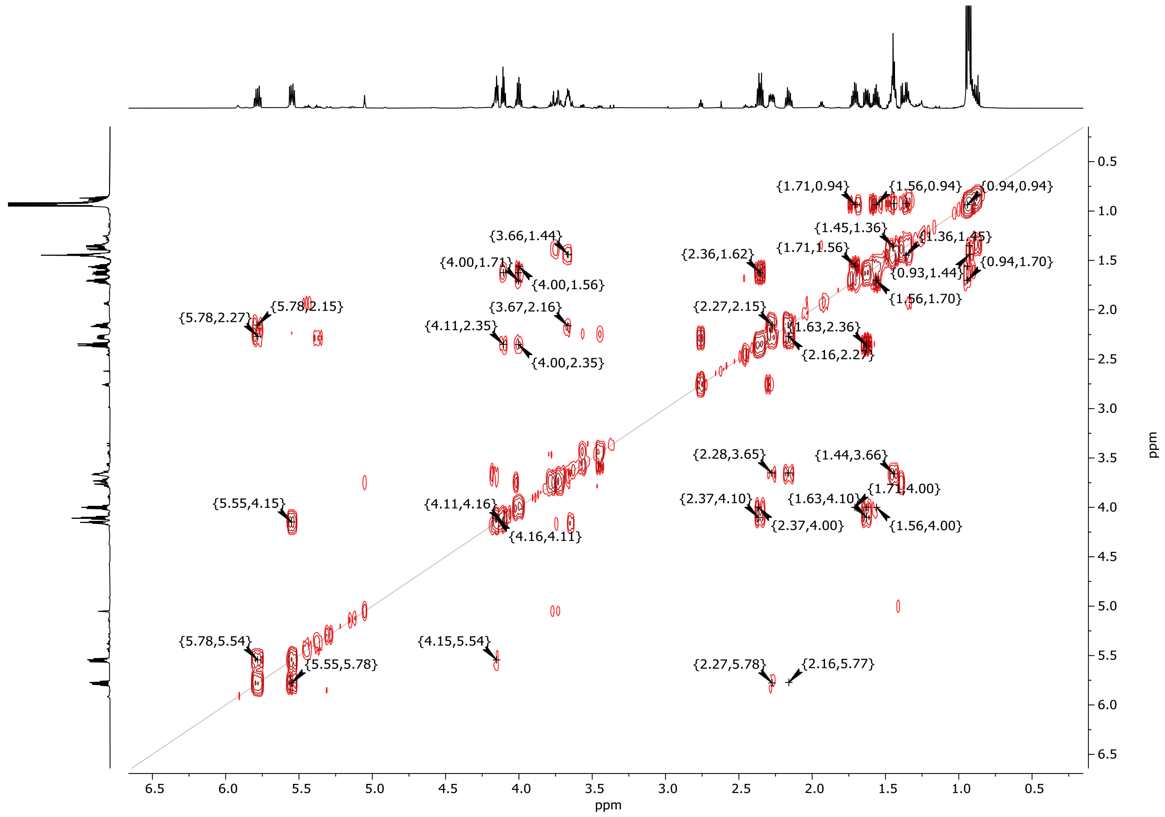

COSY NMR spectrum (700 MHz, $CDCl_3$) of **25**.

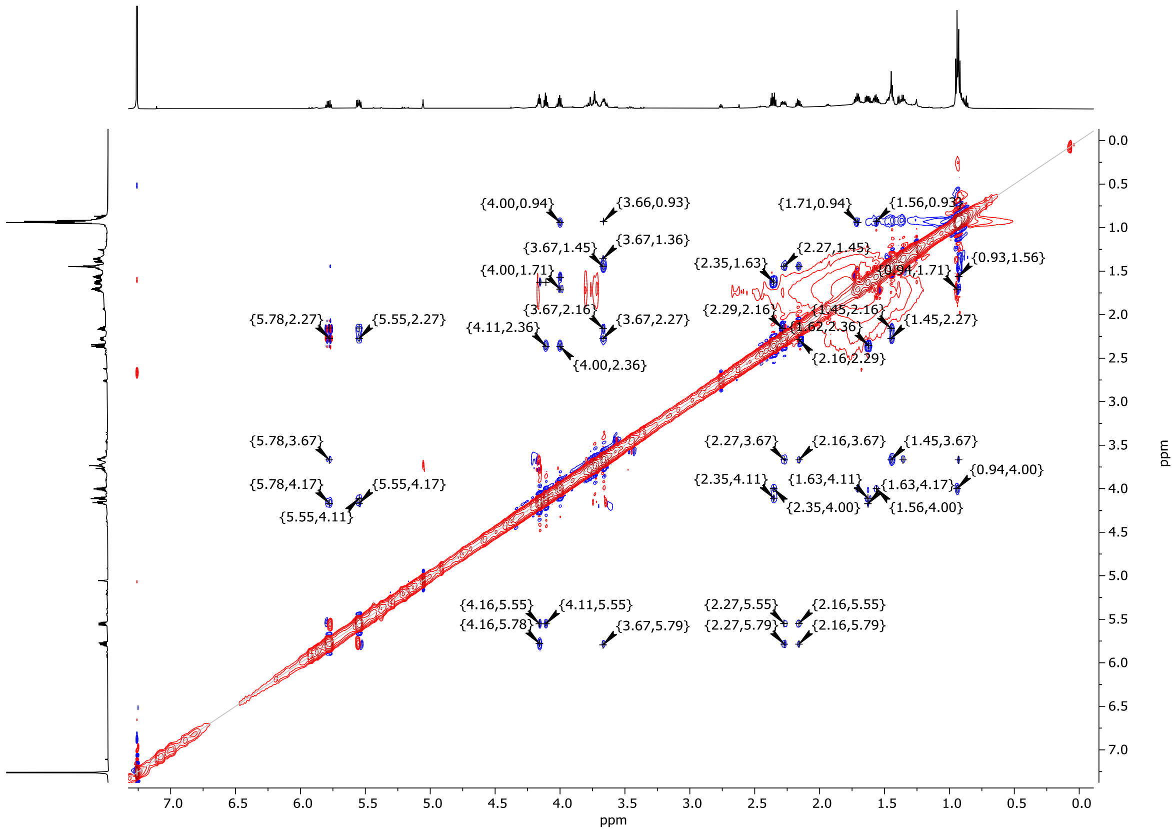

NOESY NMR spectrum (700 MHz, $CDCl_3$) of **25**.

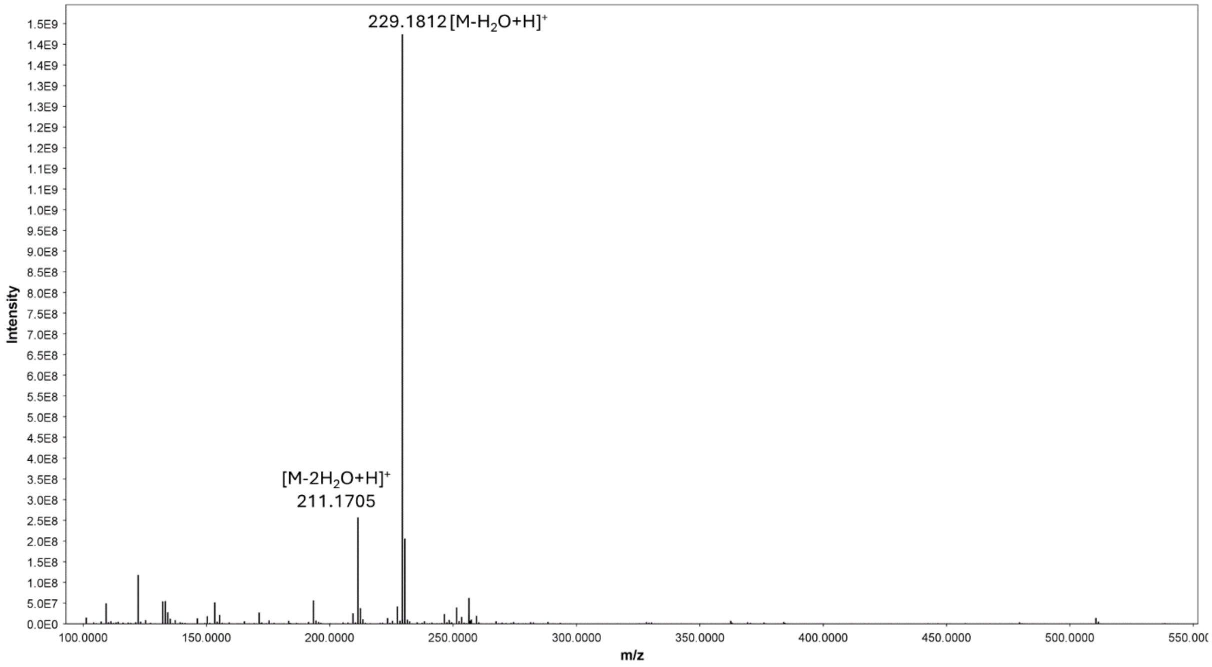

HRMS-ESI+-QTOF of **25**.

S20.Spectroscopic and spectrometric data of compounds isolated from IQ-807

iso-Cladospolide B (**24**)

Solid white; HR-ESI+-QTOF, *m/z* 229.1448 $[M+H]^+$ (calculated for $C_{12}H_{21}O_4$, 229.1439, Δ 3.9 ppm); ^{1}H NMR (700 MHz, $CDCl_3$) and ^{13}C NMR (175 MHz, $CDCl_3$) see below. Comparable data with Franck *et al*. (2001).[9]

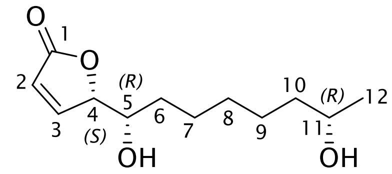

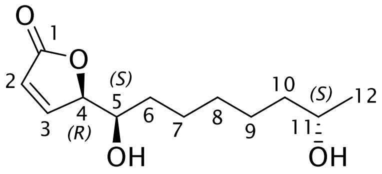

Spectroscopy data for **24** in $CDCl_3$

Position	Type	δ_C ppm	δ_H ppm	Multiplicity (*J* in Hz)
1	C	172.9		
2	CH	123.0	6.19	dd, *J* = 5.71, 2.05.
3	CH	153.7	7.45	dd, *J* = 5.81, 1.61.
4	CH	86.2	4.98	dt, *J* = 4.73, 1.83.
5	CH	72.0	3.75	dt, *J* = 6.45, 4.84.
6	CH2	33.3	1.6	m
7	CH2	29.5	1.33-1.39	m
8	CH2	25.55[a]	1.39-1.48[b]	m
9	CH2	25.72[a]	1.54[b]	m
10	CH2	39.3	1.46	m
11	CH	68.2	3.80	m
12	CH3	23.7	1.19	d, *J* = 6.13.

[a, b] Assignments may be interchanged.

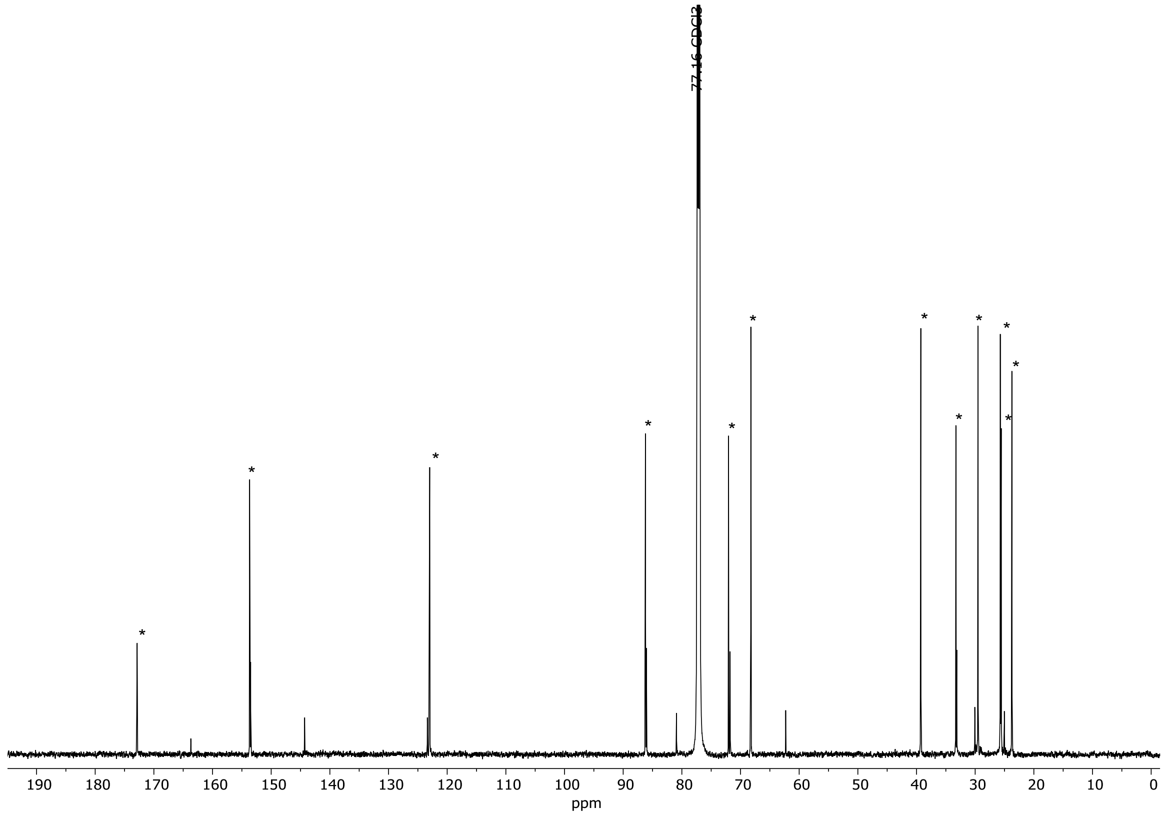

* Compound signals.

^{13}C NMR spectrum (175 MHz, $CDCl_3$) of **24**.

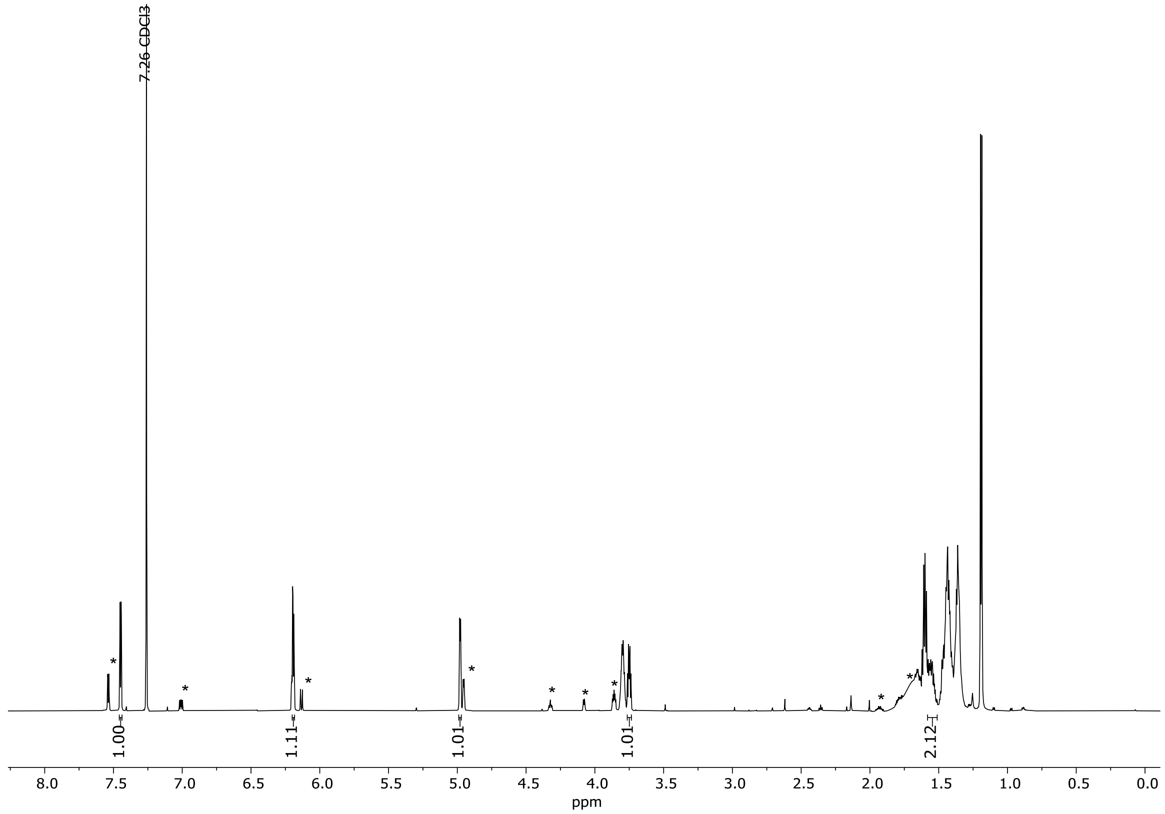

* Impurities.

^{1}H spectrum (700 MHz, $CDCl_3$) of **24**.

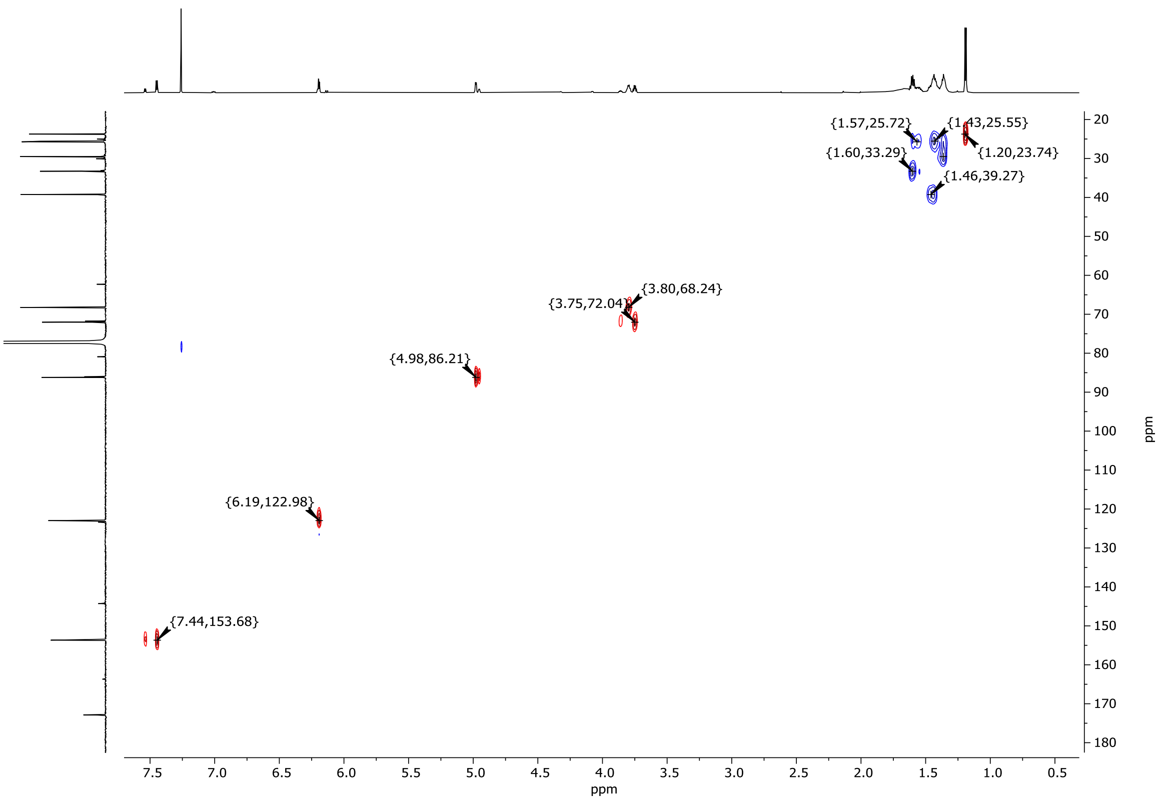

HSQC NMR spectrum (700 MHz, $CDCl_3$) of **24**.

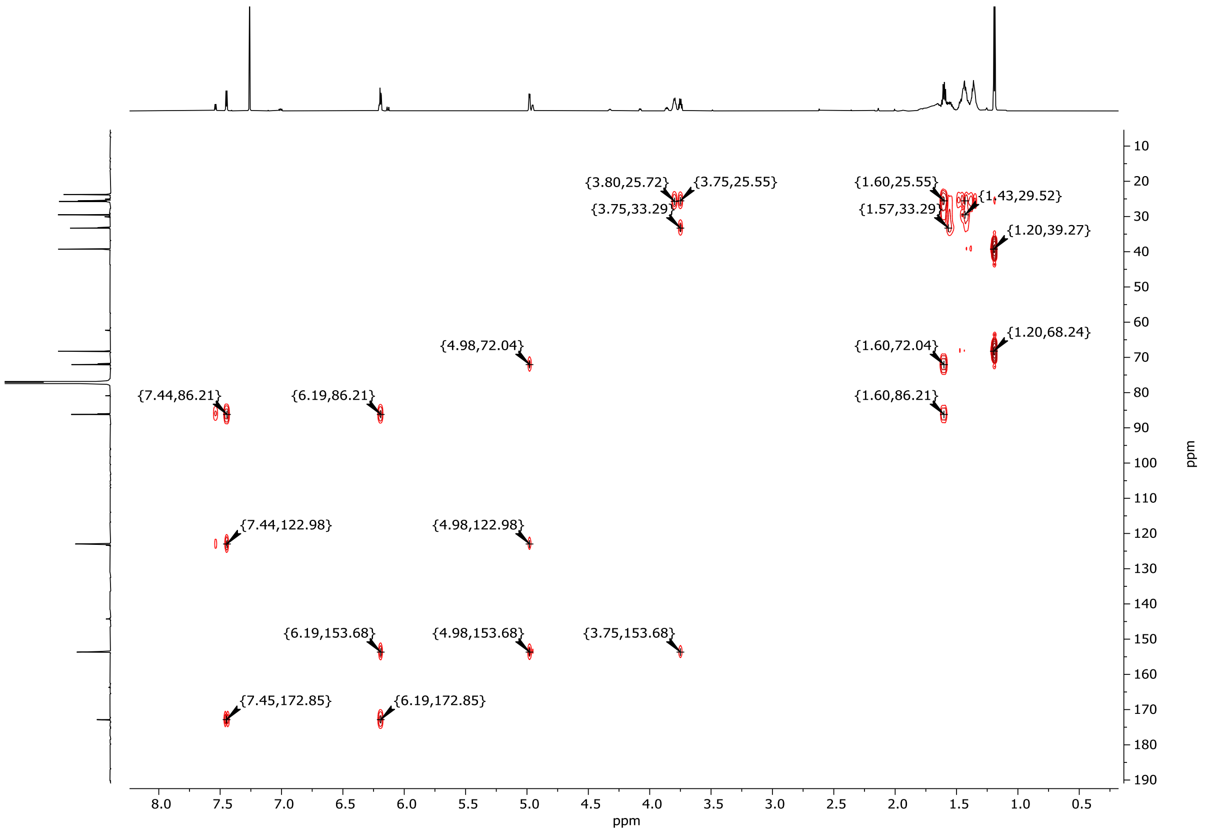

HMBC NMR spectrum (700 MHz, $CDCl_3$) of **24**.

S21.Scatter plot of the bioactive features in NPAnalyst analysis

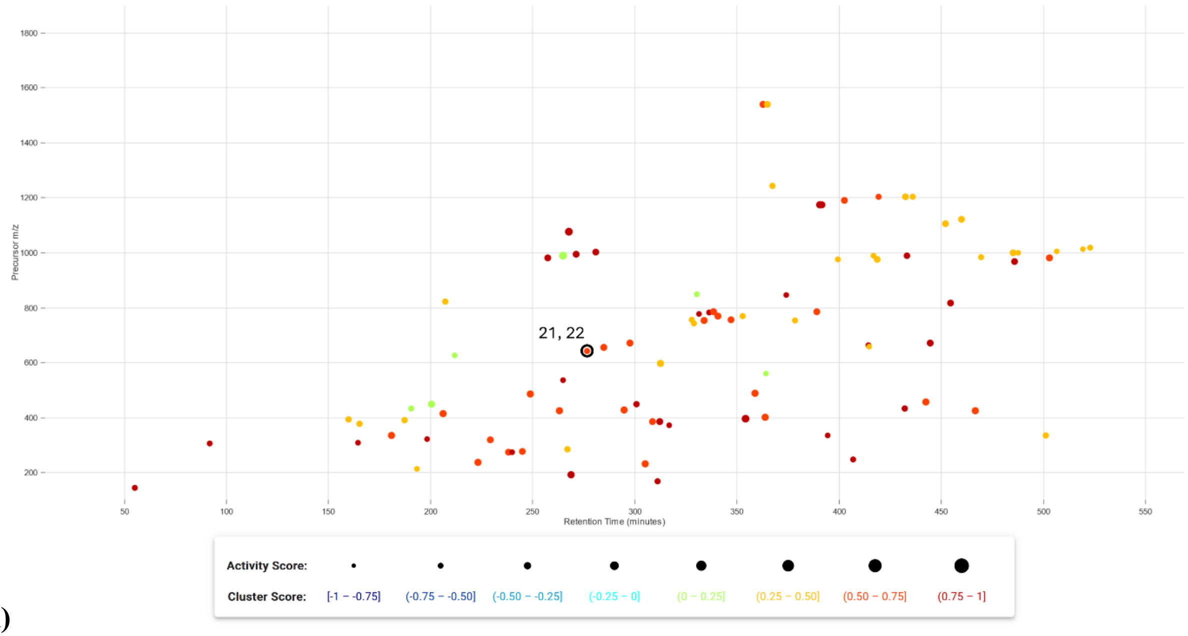

(A)

Scatter plot of 90 nodes presented in **Fig. 6**. The filtering parameters were *m/z* ratio from 121 to 1804, retention time from 11 to 569 s, frequency from 1 to 50 (number of morphotypes associated to the node), activity score from 0.25 to 2 (maximum value: 0.73) and cluster score from 0.1 to 1 (maximum value: 1). The enclosed nodes correspond to features of isolated compound.

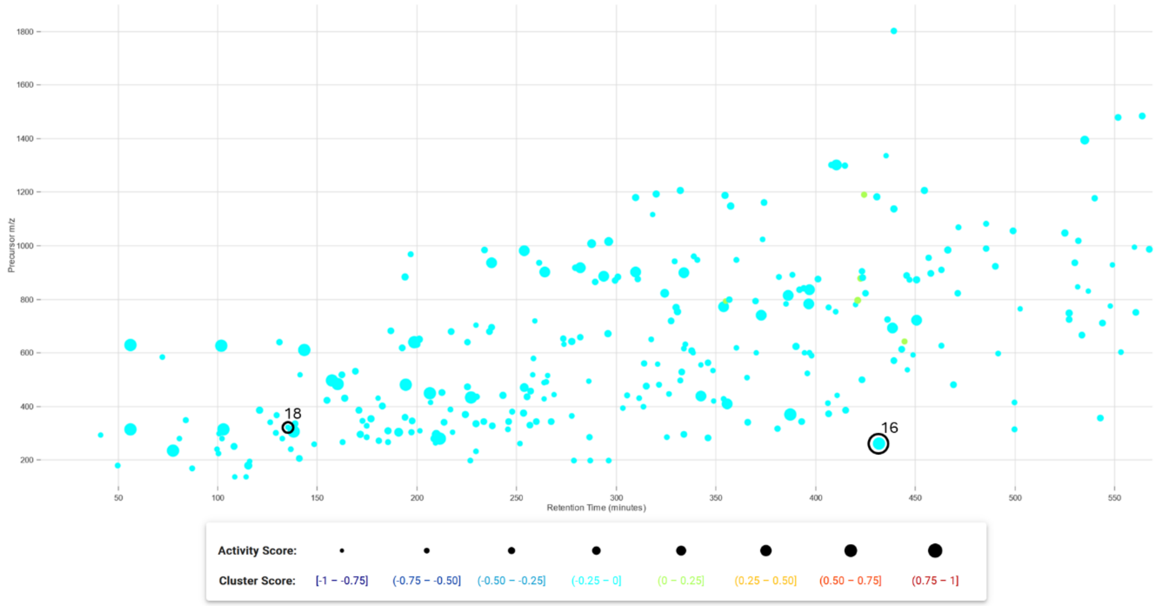

(B)

Scatter plot of 276 nodes not included in **Fig. 6**. The filtering parameters were *m/z* ratio from 121 to 1804, retention time from 11 to 569 s, frequency from 1 to 50 (number of morphotypes associated to the node), activity score greater from 0.25 to 2 (maximum value: 1.7) and cluster score from 0 to 0.099 (maximum value: 0.09). The enclosed nodes correspond to features of isolated compound.

S22.References

1. De Silva ED, Geiermann A-S, Mitova MI, et al. Isolation of 2-Pyridone Alkaloids from a New Zealand Marine-Derived *Penicillium* species. *J Nat Prod*. 2009;72(3):477–479. doi:10.1021/np800627f

2. Nonaka K, Chiba T, Suga T, et al. Coculnol, a new penicillic acid produced by a coculture of *Fusarium solani* FKI-6853 and *Talaromyces* sp. FKA-65. *J Antibiot (Tokyo)*. 2015;68(8):530–532. doi:10.1038/ja.2015.15

3. Glaser R, Shiftan D, Froimowitz M. NMR structure determination of brefeldin-A, a 13-membered ring fungal metabolite. *Magn Reson Chem*. 2000;38(4):274–280. doi:10.1002/(SICI)1097-458X(200004)38:4<274::AID-MRC630>3.0.CO;2-M

4. Vurro M, Evidente A, Andolfi A, et al. Brefeldin A and α,β-dehydrocurvularin, two phytotoxins from *Alternaria zinniae*, a biocontrol agent of *Xanthium occidentale*. *Plant Science*. 1998;138(1):67–79. doi:10.1016/S0168-9452(98)00131-9

5. Shiina I, Umezaki Y, Ohashi Y, et al. Total synthesis of AMF-26, an antitumor agent for inhibition of the golgi system, targeting adp-ribosylation factor 1. *J Med Chem*. 2013;56(1):150–159. doi:10.1021/jm301695c

6. Li D, Chen Y, Tao M, et al. Two New Octahydronaphthalene Derivatives from *Trichoderma spirale*, an Endophytic Fungus Derived from *Aquilaria sinensis*. *Helv Chim Acta*. 2012;95(5):805–809. doi:10.1002/hlca.201100417

7. Sofian FF, Warahapsari FA, Yoshida J, et al. Two new octahydronaphthalene derivatives, trichodermic acids C and D produced by *Trichoderma* sp. HN-1.1. *Nat Prod Res*. 2023;37(3):484–493. doi:10.1080/14786419.2021.1983811

8. Garo E, Starks CM, Jensen PR, et al. Trichodermamides A and B, Cytotoxic Modified Dipeptides from the Marine-Derived Fungus *Trichoderma virens*. *J Nat Prod*. 2003;66(3):423–426. doi:10.1021/np0204390

9. Franck X, Vaz Araujo ME, Jullian J-C, et al. Synthesis and structure determination of *iso*-cladospolide B. *Tetrahedron Lett*. 2001;42(15):2801–2803. doi:10.1016/S0040-4039(01)00323-9
